# Ling-gui-zhu-gan promotes adipocytes browning via targeting the miR-27b/PRDM16 pathway in 3T3-L1 cells

**DOI:** 10.3389/fphar.2024.1386794

**Published:** 2024-08-14

**Authors:** Zimengwei Ye, Yi Zhao, Yanjing Cui, Bingrui Xu, Fan Wang, Dandan Zhao, Guangtong Dong, Zhufeng Wang, Rui Wu

**Affiliations:** ^1^ School of Traditional Chinese Medicine, Beijing University of Chinese Medicine, Beijing, China; ^2^ Department of Endocrinology, Guang’anmen Hospital, China Academy of Chinese Medical Sciences, Beijing, China; ^3^ Department of Endocrinology, Guang’anmen Hospital South Campus, China Academy of Chinese Medical Sciences, Beijing, China

**Keywords:** obesity, Ling-gui-zhu-gan, browning, 3T3-L1 adipocytes, MiR-27b/PRDM16 pathway

## Abstract

**Introduction:** Obesity, a global epidemic, is caused by an imbalance between energy intake and expenditure. The induction of white adipose browning to increase heat production has emerged as a potential effective strategy to address obesity. Ling-gui-zhu-gan (LGZG), a traditional Chinese medicine formula, has been proved to achieve promising results to combat obesity and related metabolic diseases, yet the mechanisms remain largely unexplored. This study aimed to elucidate the anti-obesity properties and the mechanisms of LGZG by investigating its browning effect on 3T3-L1 adipocytes.

**Methods:** LGZG-containing serum obtained by oral administration of LGZG to animals was added to 3T3-L1 adipocytes to simulate *in vivo* conditions.

**Results:** The results showed that 49 compounds were identified in LGZG-containing serum by UHPLC-Q-Orbitrap HRMS, including compounds such as atractylenolides and polyporenic acid C, etc. LGZG-containing serum alleviated the lipid accumulation and decreased both intracellular and extracellular triglyceride contents in a dose-dependent manner. This reduction is accompanied by enhanced mitochondrial respiratory and heat production function. Mechanistically, LGZG-containing serum led to a decrease in miR-27b expression and an increase in the mRNA and protein levels of browning-related markers, including UCP1, PRDM16, PGC-1α, PPARγ, CTBP1, and CTBP2. Further investigation using miR-27b mimic transfection confirmed that miR-27b/PRDM16 pathway might be a potential mechanism by which LGZG-containing serum promotes browning of 3T3-L1 adipocytes.

**Discussion:** These results underscore the therapeutic potential of LGZG in addressing obesity and its associated metabolic disorders through the promotion of adipose browning.

## 1 Introduction

Obesity is a critical social problem worldwide, contributing significantly to various chronic diseases such as cardiovascular disease, type 2 diabetes, nonalcoholic fatty liver disorder, cancer, and hypertension (Polyzos et al., 2019; Ruze et al., 2023; Irfan, 2024). It’s the result of excessive fat accumulation caused by imbalance between caloric intake and expenditure. While calorie restriction remains an essential approach for counteracting obesity, increasing energy expenditure has recently gained wide attention. Recent research has spotlighted the conversion of white adipocytes into energy-expending beige adipocytes as a promising approach to counteract obesity (Sakers et al., 2022; Schirinzi et al., 2023). This process, known as white adipose browning, is influenced by several stimuli, including cold exposure, exercise, hormones, medications, etc., highlighting the metabolic flexibility of adipose tissues (Ikeda et al., 2018). Accumulated evidence supports that promoting white fat browning can facilitate weight loss, enhance insulin sensitivity, and improve metabolic health (Fang et al., 2015; Li et al., 2022).

Uncoupling protein 1 (UCP1), a mitochondrial protein specific to brown adipose tissues (BAT), plays a key role in converting chemical energy into heat through uncoupling mitochondrial oxidative phosphorylation from ATP synthesis, thereby serving as a major biomarker of adipose browning (Ikeda and Yamada, 2020). The browning process, regulated by factors, including PR domain containing 16 (PRDM16), peroxisome proliferator activated receptor γ (PPARγ), and PPARγ coactivator-1α (PGC-1α) enhances non-shivering thermogenesis. PRDM16 is an essential regulator to turn on program of adipocytes browning. PRDM16’s absence leads to reduced cold tolerance and diminished browning feature in white adipose tissues (WAT) (Peng et al., 2021). Concurrently, knockdown of PRDM16 in white adipocytes severely downwards the expressions of thermogenesis-related factors, such as PGC-1α, PPARγ, and UCP1(Peng et al., 2021). Simultaneously, PRDM16 also recruits repressive complexes, C-terminal binding proteins 1 and 2, to inhibit the activation of the white fat gene program (Kajimura et al., 2008).

Additionally, microRNA (miRNA), particularly miR-27b, regulates fat browning by influencing key genes involved in the process (Price and Fernández-Hernando, 2016). The miR-27b functions as a core upstream negative regulator in beige adipocytes differentiation by targeting PRDM16. Research indicated that miR-27b deficiency in white adipocytes initiated the thermogenic program and showed marked increase in the expression of thermogenesis-related factors, such as PRDM16, PGC-1α, and UCP1 (Sun and Trajkovski, 2014). Most notably, miR-27b could suppress WAT browning by directly targeting PRDM16, thereby promoting abnormal fat accumulation (Kong et al., 2015).

Exploitation on browning agents aimed at fat browning induction is increasing recently. Ling-gui-zhu-gan (LGZG), a classical traditional Chinese medicine (TCM) prescription which was recorded in the Shang han za bing lun, has been traditionally utilized to treating syndrome of phlegm retention due to deficiency of Yang (Li et al., 2018). In modern medicine, LGZG has gained attention for its potential benefits in metabolic disorders, particularly obesity. Clinical trials have confirmed LGZG’s satisfactory effects on promoting weight loss and improving glycolipid metabolism disorders in obese patients with syndrome of phlegm-dampness due to spleen deficiency (Luo et al., 2021; Li Q. et al., 2023), potently reversing hyperglycemia and dyslipidemia in patients with metabolic syndrome (Yang et al., 2014), and significantly alleviating liver steatosis in patients with non-alcoholic fatty liver disease (Xu et al., 2020). Meanwhile, basic studies have validated LGZG’s significant anti-inflammatory and anti-oxidative stress effects (Han et al., 2022), and its ability to modulate the composition of the fecal microbiota and its metabolites to attenuate high-fat diet-induced insulin resistance (IR) (Wu et al., 2019; Ning et al., 2022).

LGZG consists of four botanical drugs: *Smilax glabra* Roxb. [Smilacaceae; Poria], *Neolitsea cassia* (L.). Kosterm. [Lauraceae; Cinnamomi Ramulus], *Atractylodes macrocephala* Koidz. [Asteraceae; Atractylodis macrocephalae Rhizoma], and *Glycyrrhiza glabra* L. [Fabaceae; Glycyrrhizae Radix et Rhizoma]. Traditionally, the components in LGZG have been individually utilized for their various medicinal properties, including promoting digestion, reducing phlegm, and tonifying the spleen. Recent studies have identified various phytochemicals within LGZG that contribute to its therapeutic effects. These phytochemicals include flavonoids, triterpenes, phenylpropanoids, organic acids, lactones, alkaloids, anthraquinones, quinones, and other active ingredients (Li et al., 2021), which have been shown to have anti-inflammatory, antioxidant, and lipid-lowering properties. In order to simulate the metabolic process of LGZG *in vivo*, LGZG-containing serum was added into cells to evaluate its pharmacological effects on adipocytes and further explore the undying mechanisms of LGZG. In addition, metformin is widely recognized for its beneficial effects on lipid metabolism, probably through enhancing adipose browning (Ziqubu, et al., 2023). Therefore, we included metformin as a positive control in our study to benchmark the effects of LGZG-containing serum on 3T3-L1 adipocytes. Hence, this study delves into LGZG’s role in fat browning by examining its effect on lipid accumulation and mitochondrial function in 3T3-L1 adipocytes, as well as its capacity to trigger the browning program through the miR-27b/PRDM16 pathway, aiming to elucidate its role in promoting metabolic health and combating obesity.

## 2 Materials and methods

### 2.1 Preparation of LGZG-containing serum

LGZG granules were composed of *S. glabra* Roxb. [Smilacaceae; Poria], *Neolitsea cassia* (L.). Kosterm. [Lauraceae; Cinnamomi Ramulus], *Atractylodes macrocephala* Koidz. [Asteraceae; Atractylodis macrocephalae Rhizoma], and *Glycyrrhiza glabra* L. [Fabaceae; Glycyrrhizae Radix et Rhizoma] at a ratio of 4:3:3:2. The granules were obtained from Jiangyin Tianjiang Pharmaceutical Co., Ltd. (Jiangyin, China), and were prepared in accordance with the established processing guidelines, including extraction, separation, concentration, drying, granulation, screening, mixing, and packaging in the “Technical Requirements for Quality Control and Standard Formulation of Chinese Medicine Formula Granules” issued by the China National Medical Products Administration. In this experiment, the LGZG granules were suspended in sterilized water to the appropriate concentration for further intragastric administration.

Twenty male Sprague-Dawley (SD) rats (aged 8 weeks, mean weight 280 ± 20 g), license number SCXK (Beijing) 2019–0010, were purchased from Beijing Sibeifu Animal Technology Co., Ltd. (Beijing, China). The entire protocol was approved by the Animal Care Committee of Beijing University of Chinese Medicine (Certificate No: BUCM-1-2023012001-0025). The rats were housed in a pathogen-free environment with unlimited access to water and food under controlled conditions (a room temperature at 23°C ± 2°C, a relative humidity of 55% ± 10%, a 12 h light/dark cycle). One week after the acclimation, all rats were randomly divided into two groups (*n* = 10 per group): the LGZG-containing serum group (rats received 7.608 g/kg/d LGZG granules) and the blank control group (rats received the same volume of distilled water). The intragastric gavage was performed to rat in each group twice a day for 3 consecutive days. Two hours post-gavage, rats were ether-anesthetized, and blood was taken from abdominal aorta. The blood was then centrifuged to obtain serum, which was heat-inactivated, filtered through 0.22 μm membrane (SLGPR33RB, Millipore, Burlington, MA, United States), and stored at −80°C for further use.

The chemical characterization of LGZG-containing serum was performed by ultra-performance liquid chromatograph-hybrid quadrupole orbitrap high resolution mass spectrometer (UHPLC-Q-Orbitrap HRMS) analysis. The main constituents was detected and presented in Supplementary Figure S1; Supplementary Table S1. A total of 49 constituents were identified from the LGZG-containing serum through data matching, consisting of 11 flavonoids, 9 isoflavonoids, 2 neoflavonoids, 17 triterpenoids, 2 bile acids, alcohols and derivatives, 1 chalcones and dihydrochalcones, 4 terpene glycosides, 2 terpene lactones, 1 naphthofurans.

### 2.2 Cell culture and differentiation

Murine 3T3-L1 preadipocytes were obtained from the Cell Resource Center, Peking Union Medical College (Beijing, China, #1101MOU-PUMC000155) and cultured in Dulbecco’s Modified Eagle’s Medium (DMEM; 6123153, Gibco, Grand Island, NY, United States) supplemented with 10% (v/v) fetal bovine serum (FBS, 10099141) and 1% (v/v) antibiotics (100× penicillin-streptomycin, 15240062) in a 37°C humidified incubator with 5% CO_2_. For differentiation, after reaching 80% confluence (designated as day 0), the culture medium was replaced with DMEM containing 10% FBS, 0.5 mM 3-isobutyl-1-methylxanthine (IBMX; B7206, APExBIO, Houston, TX, United States), 1 µM dexamethasone (DEX, A2324), and 1 μg/mL insulin (B7407) for the initial 2 days (day 2), followed by DMEM containing 10% FBS and 1 μg/mL insulin for another 2 days (day 4). The medium was renewed every 2 days with 10%FBS-DMEM until fully differentiation into mature adipocytes were observed (day 8) (Tu et al., 2020; Kim et al., 2022). From day 0 to day 8, the cells were incubated with or without LGZG-containing serum or metformin (B1970; APExBIO, Houston, TX, United States).

### 2.3 Cell viability assay

3T3-L1 cells were plated at a density of 2 × 10^4^/well in 96-well plates, and were differentiated and treated as described previously. Then cell viability was assessed using the Cell Counting Kit-8 (CCK-8; AQ308, Aoqing Biotechnology Ltd., Beijing, China) according to the operating instructions.The absorbance at 450 nm of each well was detected after incubated with CCK-8 agent using a microplate reader (Thermo Fisher Scientific, Waltham, MA, United States). Cell viability (%) was calculated as (OD _Sample_–OD _Blank_) / (OD _Control_–OD _Blank_) × 100%.

### 2.4 Oil red O staining

Oil red O staining (G1262, Solarbio, Beijing, China) was conducted to investigate intracellular lipid accumulation. In short, 3T3-L1 adipocytes were subjected to established processes, including washing twice with phosphate-buffered saline (PBS, AQ10010), fixing with the oil red O fixative solution for 30 min, rinsing in 60% isopropyl alcohol, and staining with freshly prepared oil red O solution for 30 min. Post-staining, the cells were rinsed with distilled water for observation under an inverted microscope at 400 × magnification (Olympus Corporation, Tokyo, Japan). Then the images with stained lipid droplets were captured (Li J. et al., 2023). Lipid accumulation was quantified was calculated using image J software based on the relative area of lipid droplets evaluate (Mehlem et al., 2013).

### 2.5 Triglyceride (TG) measurement

After differentiation and treatment as described, the cells lysates and media were harvested to detect the intracellular and extracellular TG content using a commercial kit (A110-1-1, Nanjing Jiancheng Bioengineering Institute, Nanjing, China). TG content was normalized against the protein concentration in each well (mmol/μg protein).

### 2.6 Measurement of mitochondrial oxygen consumption rate (OCR)

The OCR in 3T3-L1 cells was measured using a Seahorse XFe24 Extracellular Flux Analyzer (Seahorse Bioscience, Billerica, MA, United States) following the manufacturer’s protocols for the mitochondrial stress test kit (103015-100, Agilent Technologies, Inc., Cedar Creek, TX, United States). Briefly, cells were rinsed and incubated with Seahorse assay medium and subjected to a CO_2_-free environment for an hour before analysis. Thereafter, real-time OCR was successively measured following sequential injection of test agents at the indicated time points: 1.5 µM Oligomycin, 1.0 µM carbonyl cyanide-p-trifluoromethoxy-phenylhydrazone (FCCP), and 0.5 µM rotenone/antimycin A. Parameters were collected to evaluate the mitochondrial function in 3T3-L1 adipocytes. The basal respiration, proton leak-related respiration, maximal respiration, and spare respiratory capacity were calculated based on the following formula and normalized to the protein concentration (pmol/min/μg protein) referred to the previous description (Nasci et al., 2019; Sun et al., 2020): basal respiration = the basal OCR–non-mitochondrial OCR, respiration related to proton leak = the OCR after Oligomycin–non-mitochondrial OCR, maximal respiration = the OCR after FCCP injection–non-mitochondrial OCR, spare respiratory capacity = the OCR after FCCP injection–the basal OCR.

### 2.7 MiR-27b transfection

3T3-L1 cells were seeded into 12- or 6-well plates at a density of 2 × 10^5^/mL. After reaching 70%–80% confluence, the cells were transfected with miR-27b mimics (R307215466 and R307215467, KeyGen Biotech, Nanjing, China) or a negative control (miR-NC mimics) using a mixture of Opti-MEM^®^ (31985062, Gibco, Grand Island, NY, United States) and Lipo8000™ (C0533, Beyotime Institute of Biotechnology, Shanghai, China). Thereafter, the cells were incubated with the transfection mixture for 48 h to allow for miRNA uptake (Gan et al., 2017; Li and Xiao, 2021).

### 2.8 Reverse transcription quantitative real-time polymerase chain reaction (RT-qPCR) analysis

RT-qPCR analysis was conducted as originally reported (Xu et al., 2019; Pacifici et al., 2023). Total RNA was isolated using Trizol reagent (A4A0615, Accurate Biology Co., Ltd., Changsha, China), then converted to cDNA by reverse transcription kit (AG11706, Accurate Biology Co., Ltd., Changsha, China). Gene amplification was conducted with SYBR Green PCR reagent (AG11718) on a RT-qPCR detection system (Bio-Rad, Hercules, CA, United States). The primer sequences (100194855, Sangon Biotech Co., Ltd., Shanghai, China) used for the mRNAs were as follows: UCP1-F: 5’-GAA​ACA​CCT​GCC​TCT​CTC​GGA​AAC-3’, UCP1-R: 5’-GCA​TTC​TGA​CCT​TCA​CGA​CCT​CTG-3’; PRDM16-F: 5’-CAG​CAA​CCT​CCA​GCG​TCA​CAT​C-3’, PRDM16-R: GCG​AAG​GTC​TTG​CCA​CAG​TCA​G-3’; PGC-1α-F: 5’-CGA​TGA​CCC​TCC​TCA​CAC​CAA​AC-3’, PGC-1α-R: 5’-TTG​CGA​CTG​CGG​TTG​TGT​ATG​G-3’; CTBP1-F: 5’-CAG​TGA​GCA​GGC​GTC​CAT​TGA​G-3’, CTBP1-R: 5’-GGC​TGT​CAG​GTG​GTC​CTT​GTT​G-3’; CTBP2-F: 5’-TGA​GAA​GGT​GTT​GAA​TGA​GGC​TGT​G-3’, CTBP2-R: 5’-CAC​TAC​CGA​TTC​GCA​CGA​TCA​CTC-3’; β-actin-F: 5’-GTG​CTA​TGT​TGC​TCT​AGA​CTT​CG-3’, β-actin-R: 5’-ATG​CCA​CAG​GAT​TCC​ATA​CC-3’, while the primers for miR-27b and U6: miR-27b-F: 5’-UUC​ACA​GUG​GCU​AAG​UUC​UGC-3’, miR-27b-R: 5’-GCA​GAA​CUU​AGC​CAC​UGU​GAA​A-3’; U6-F: 5’-ACC​CTG​AGA​AAT​ACC​CTC​ACA​T-3’, U6-R: 5’-GAC​GAC​TGA​GCC​CCT​GAT​G-3’. The relative expressions of the mRNAs to the endogenous control β-actin, and miR-27b to U6 were calculated using 2^−ΔΔCt^ method.

### 2.9 Western blot analysis

Western blot experiments for 3T3-L1 cells followed standard protocols, including protein extraction, concentration determination, sample loading, electrophoresis, membrane transfer, and blocking with 5% skim milk (Choi et al., 2023). Immediately after, the membrane was incubated with corresponding primary antibodies against UCP1 (23673-1-AP), PRDM16 (PA5-20872), PGC-1α (66369-1-Ig), PPARγ (16643-1-AP), CTBP1 (10972-1-AP), and CTBP2 (10346-1-AP) at 1:1,000 dilution, and a secondary antibody against β-Tubulin (66240-1-Ig) at 1:10,000 (all from Proteintech Group, Chicago, IL, United States). The objective bands were developed using a hypersensitive luminescent reagent (P10100, NCM Biotech, Suzhou, China), visualized with a gel system (Clinx, Shanghai, China) and imaged. Quantitative analysis of the target protein levels relative to the internal control β-Tubulin were performed using image J software.

### 2.10 Statistical analysis

All data were expressed as the mean ± standard error of mean (SEM) and were statistically analyzed by one-way analysis of variance (ANOVA) followed with Dunnett’s test for multiple comparison test using GraphPad prism 7 software. Statistical significance among groups was determined at *p* < 0.05.

## 3 Results

### 3.1 LGZG-containing serum decreased lipid accumulation in 3T3-L1 adipocytes

**FIGURE 1:**
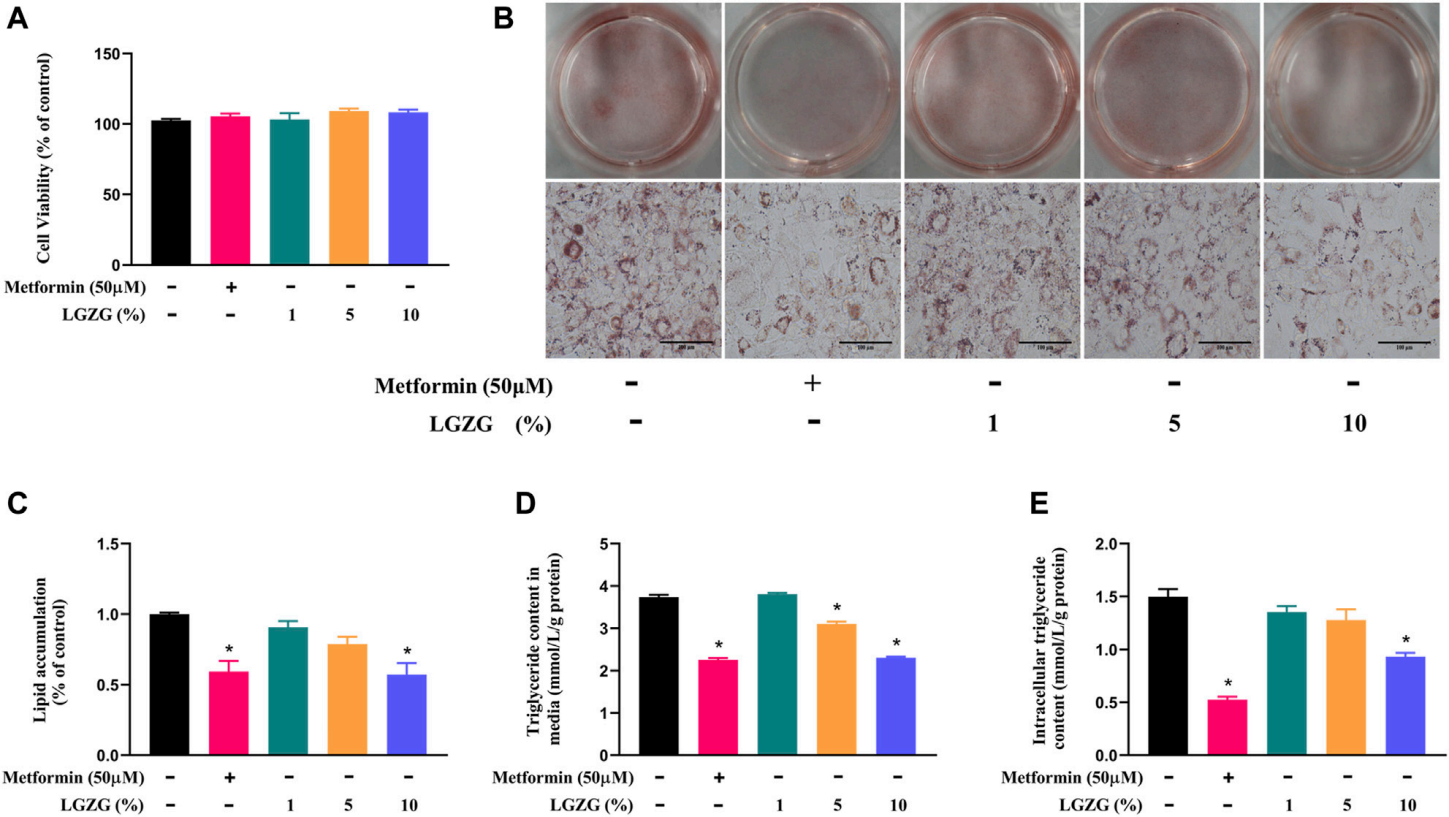
The effect of LGZG-containing serum on lipid accumulation in 3T3-L1 adipocytes. **(A)** Cell viability of 3T3-L1 adipocytes exposed to the absence or presence of various concentrations of LGZG-containing serum (1%, 5%, and 10%). **(B)** Oil red O staining images of lipid droplets in 3T3-L1 adipocytes (magnification ×400) and **(C)** its gray value analysis using image J. **(D)** Triglyceride content in culture media. **(E)** Intracellular triglyceride content. All data were presented as mean ± SEM, with *n* = 4–6 for each group in **(A–E)**. *p < 0.05 versus the non-treated 3T3-L1 adipocytes.

The cytotoxicity of LGZG-containing serum on 3T3-L1 adipocytes were evaluated by CCK-8 after being treated with or without a serious of concentrations of LGZG-containing serum (1%, 5%, and 10%). The results showed that compared with the non-treated adipocytes, 3T3-L1 adipocytes exposed to LGZG-containing serum did not evoke significant changes in cell viability (Figure 1A, *p* > 0.05). Previous studies have shown that the transformation of preadipocytes into mature white adipocytes is highly associated with the onset and development of obesity due primarily to lipid accumulation (Li et al., 2020; Oh et al., 2023). We then tested the effect of LGZG-containing serum on lipid accumulation using oil red O staining. It revealed that LGZG-containing serum reduced lipid accumulation in a concentration-dependent manner, notably at concentration of 10% (*p* < 0.05), mirroring the efficacy of metformin (Figures 1B, C). Further detection of intracellular and extracellular TG levels displayed a similar tendency to that of the oil red O staining (Figures 1D, E), indicating LGZG-containing serum has the potential to disrupt preadipocytes conversion into mature adipocytes through inhibiting lipid droplets accumulation.

### 3.2 LGZG-containing serum enhanced the mitochondrial respiratory function in 3T3-L1 adipocytes

**FIGURE 2:**
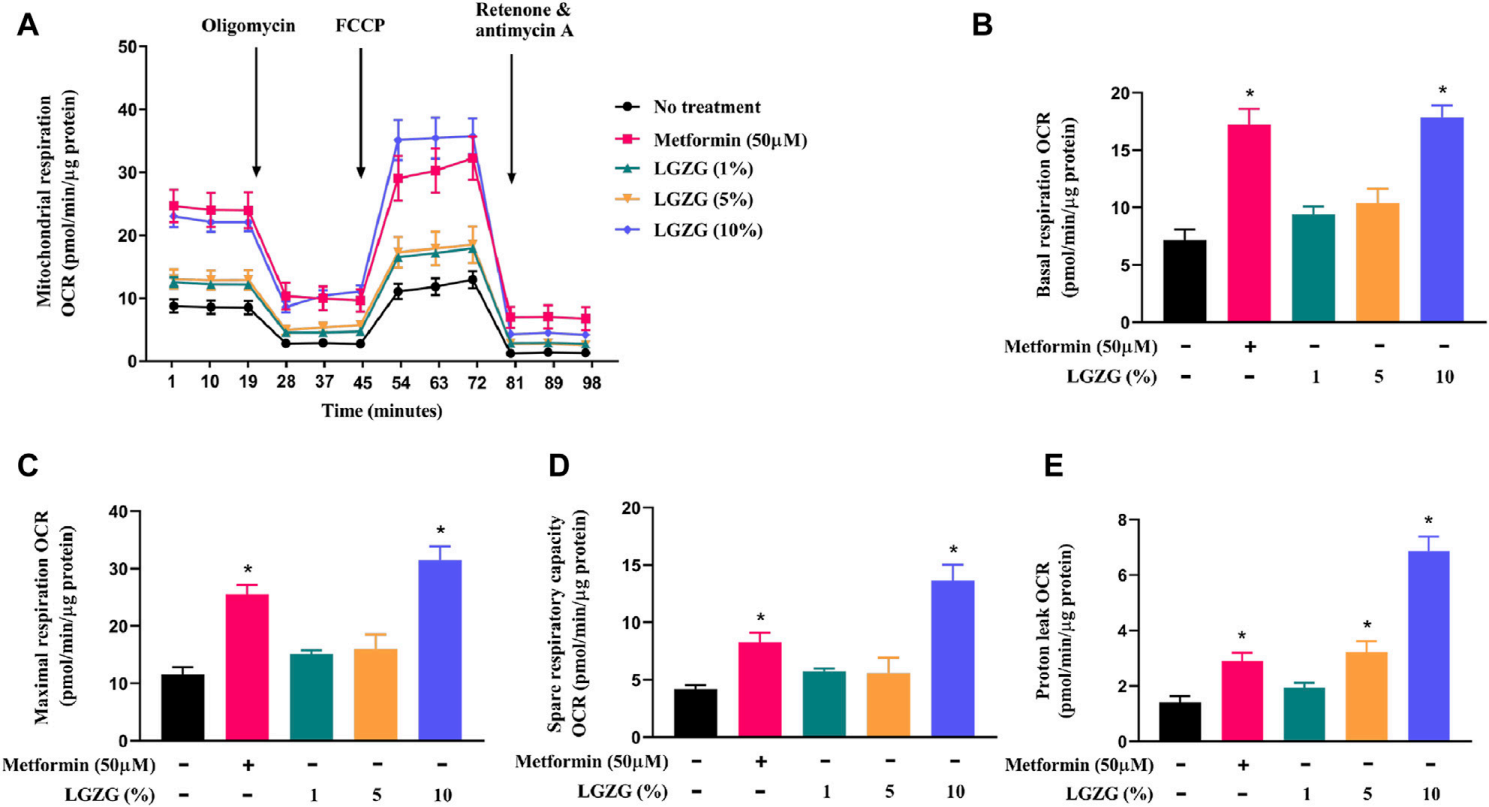
The effect of LGZG-containing serum on mitochondrial respiration in 3T3-L1 adipocytes. **(A)** The line chart for mitochondrial oxygen consumption rate (OCR). **(B)** Basal respiration, **(C)** maximal respiration, **(D)** spare respiratory capacity, and **(E)** proton leak of 3T3-L1 adipocytes. All data were presented as mean ± SEM, with *n* = 3 for each group in **(A–E)**. *p < 0.05 versus the non-treated 3T3-L1 adipocytes.

Research points out that mitochondria are the core organelles for energy dissipation in thermogenic adipocytes, where respiratory rates crucially determines heat production (Chouchani et al., 2019). To characterize the effect of LGZG-containing serum on mitochondrial function, the mitochondrial stress test was conducted on Seahorse energy metabolizer. As depicted in Figure 2A, treatment with various concentrations of LGZG-containing serum achieved a higher mitochondrial OCR than the non-treated 3T3-L1 adipocytes. Specifically, LGZG-containing serum at 1%, 5%, and 10% significantly enhanced the basal, maximal, and spare respiratory capacities, which are pivotal indicators in reflecting mitochondrial function (Figures 2B–D) (Smolina et al., 2017; Sciandra et al., 2023). Simultaneously, LGZG-containing serum increased proton leak (Figure 2E), promoting thermogenesis through mitochondrial uncoupling (Jastroch and Seebacher, 2020). The optimum promotion effect was induced by 10% LGZG-containing serum, which increased the basal respiration, leak proton, maximal respiration, and spare respiratory capacity by about 1.48-, 4.84-, 1.7-, and 2.26-fold (*p* < 0.05), respectively. This concentration also paralleled the effects seen with metformin. Consequently, a 10% LGZG-containing serum concentration was chosen to explore follow-up mechanism.

### 3.3 LGZG-containing serum downregulated miR-27b and activated browning process in 3T3-L1 adipocytes

**FIGURE 3:**
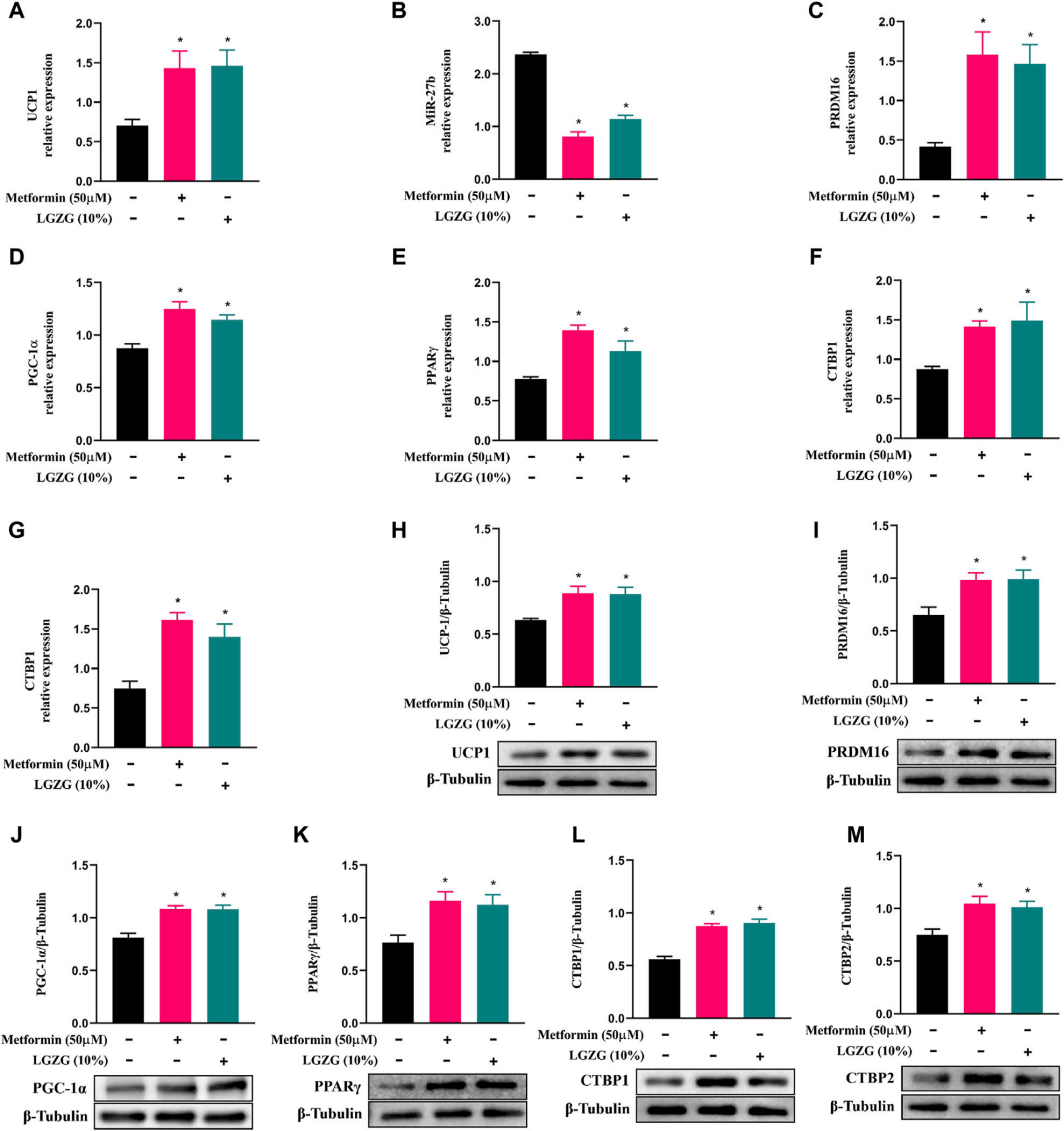
The effect of LGZG-containing serum on the expression of miR-27b and browning-related markers in 3T3-L1 adipocytes. **(A–G)** Relative mRNA levels of UCP1, miR-27b, PRDM16, PGC-1α, PPARγ, CTBP1, and CTBP2 using RT-qPCR analysis. **(H–M)** The immunoblotting images and relative protein levels of UCP1, PRDM16, PGC-1α, PPARγ, CTBP1, and CTBP2 using Western blot analysis. All data were presented as mean ± SEM, with *n* = 6 for each group in **(A–G)**, *n* = 3 for each group in **(H–M)**. *p < 0.05 versus the non-treated 3T3-L1 adipocytes.

White fat browning tends to be a major determinant of increased energy expenditure. As described above, LGZG-containing serum enhanced oxygen consumption in 3T3-L1 adipocytes, and raised mitochondrial uncoupling to increase thermogenesis, which may indicate that the browning marker UCP1 can be activated. As expected, treatment with either metformin or LGZG-containing serum resulted in a remarkable upregulation of UCP1 at both mRNA and protein levels (Figures 3A, H, *p* < 0.05), compared with the non-treated adipocytes. Investigating further, we found that metformin or LGZG-containing serum significantly downregulated miR-27b (Figure 3B), an inhibitor of white adipocytes browning that targets PRDM16 (Sun and Trajkovski, 2014). Moreover, the relative mRNA and protein levels of the PRDM16, as well as PGC-1α and PPARγ, which are master regulators in culminating UCP1 overexpression, were dramatically elevated when compared to the non-treated adipocytes. And the transcriptional repressors CTBP1 and CTBP2, which negatively interact with PRDM16 to affect white fat development, were also upregulated (Figures 3C–G, I–M, *p* < 0.05). The above results suggest a comprehensive mechanism by which LGZG facilitates adipocytes browning and energy dissipation.

### 3.4 LGZG-containing serum counteracts miR-27b’s inhibition on PRDM16-mediated browning program

**FIGURE 4:**
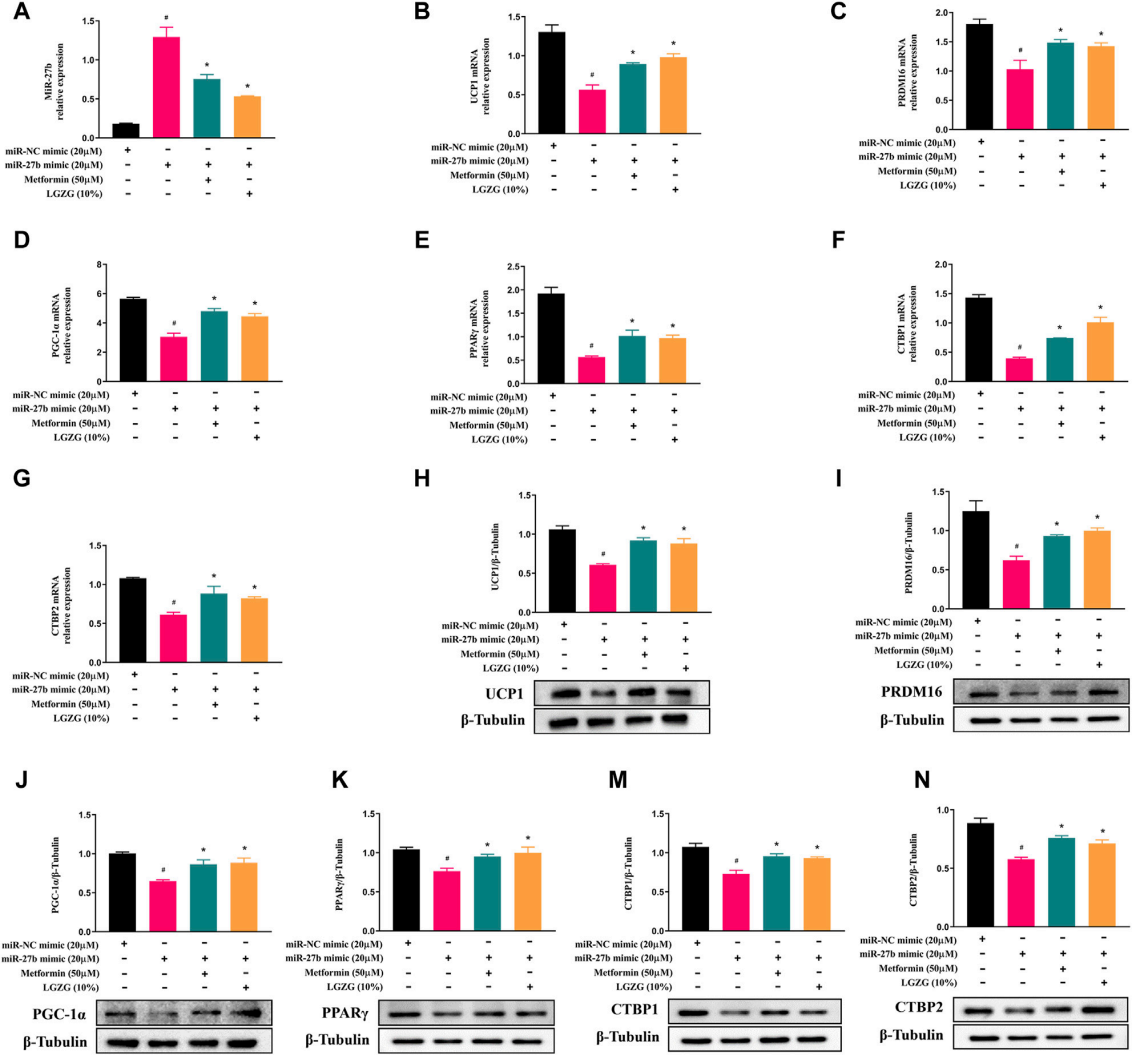
The effect of LGZG-containing serum on miR-27b/PRDM16 signaling pathway in 3T3-L1 adipocytes. **(A–G)** Relative mRNA levels of miR-27b, UCP1, PRDM16, PGC-1α, PPARγ, CTBP1, and CTBP2 after transfection with miR-27b mimic or miR-NC mimic for 48 h using RT-qPCR analysis. **(H–N)** The immunoblotting images and relative protein levels of UCP1, PRDM16, PGC-1α, PPARγ, CTBP1, and CTBP2 after transfection with miR-27b mimic or miR-NC mimic for 48 h using Western blot analysis. All data were presented as mean ± SEM, with *n* = 3 for each group in **(A–N)**. #*p* < 0.05 versus the negative control adipocytes; *p < 0.05 versus the miR-27b overexpressed adipocytes.

To test whether LGZG-containing serum activated PRDM16-mediated browning program via inhibiting miR-27b, 3T3-L1 adipocytes were transfected with a miR-27b mimic or a control. Using RT-qPCR analysis, we found that compared to the negative control adipocytes, transfection with the miR-27b mimic significantly increased the relative miR-27b levels of 3T3-L1 adipocytes (Figure 4A, *p* < 0.05). Simultaneously, the upregulated miR-27b markedly decreased the expressions of factors involved in browning induction and white fat development suppression, including PRDM16, PGC-1α, PPARγ, CTBP1, and CTBP2, both at RNA and protein levels (Figures 4B–N, *p* < 0.05). Whereas, metformin or LGZG-containing serum treatment reversed these changes (*p* < 0.05). These results indicate that miR-27b/PRDM16 signaling axis could be responsible for LGZG-containing serum’s ability to stimulate the browning program.

### 3.5 LGZG-containing serum alleviated miR-27b-induced lipid accumulation

**FIGURE 5:**
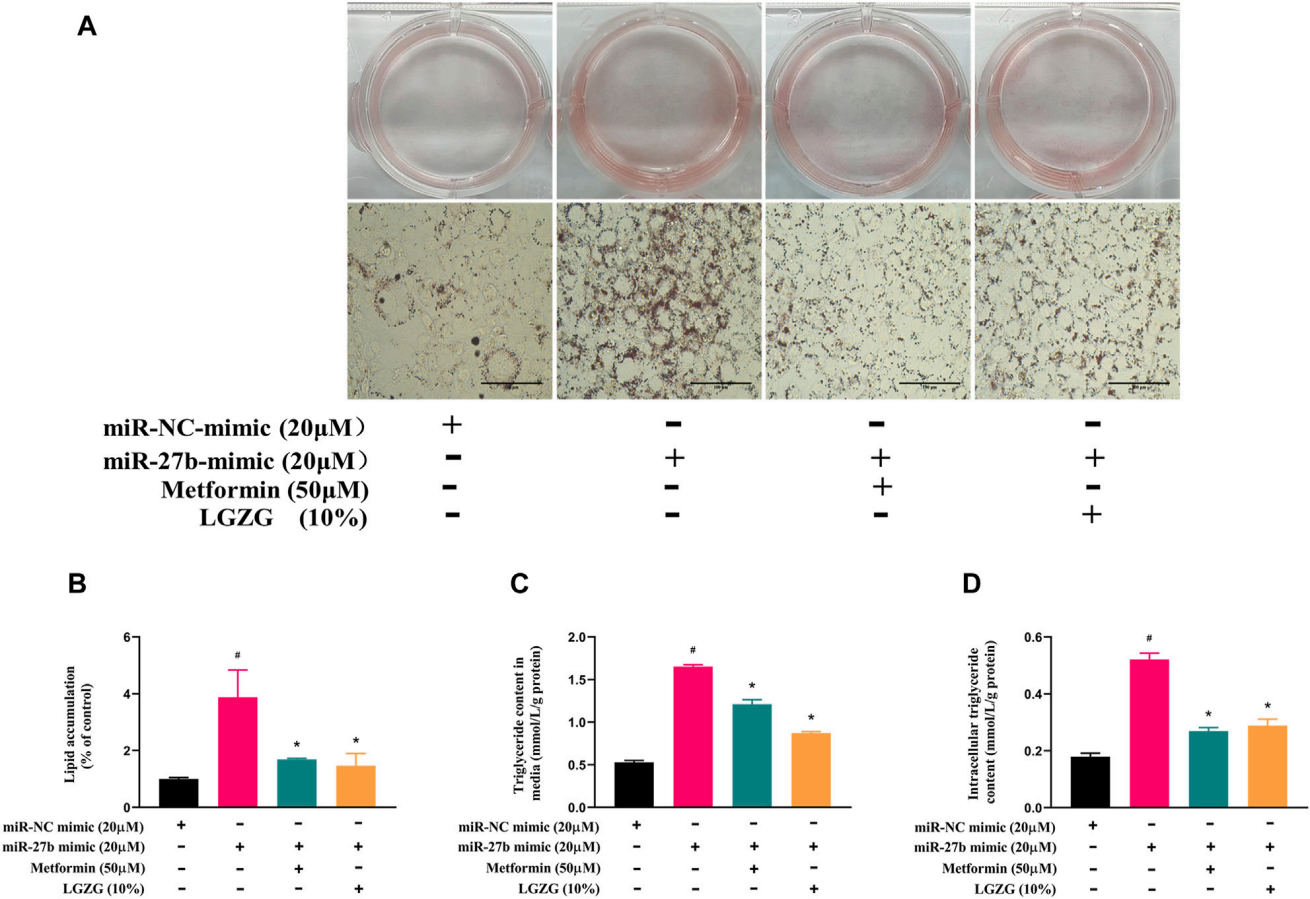
The effect of LGZG-containing serum on lipid accumulation in response to overexpressed miR-27b. **(A)** Oil red O staining images of lipid droplets in 3T3-L1 adipocytes (magnification ×400) after transfection with miR-27b mimic or miR-NC mimic for 48 h and **(B)** the gray value analysis using image J. **(C)** Extracelluar and **(D)** intracelluar triglyceride contents after transfection with miR-27b mimic or miR-NC mimic for 48 h. All data were presented as mean ± SEM, with *n* = 3–6 for each group in **(A–D)**. #*p* < 0.05 versus the negative control adipocytes; *p < 0.05 versus the miR-27b overexpressed adipocytes.

Our study previously established miR-27b as a negative regulator of adipocytes browning. Given that browning process is often accompanied by the inhibition of lipid accumulation, the changes in lipid accumulation under miR-27b overexpression were further explored. Oil red O staining showed that more and larger lipid droplets were formed in miR-27b overexpressed adipocytes compared with the negative control adipocytes (Figures 5A, B, *p* < 0.05). Both intracellular and extracellular TG contents were robustly increased with miR-27b upregulation (Figures 5C, D, *p* < 0.05). However, incubation with metformin or LGZG-containing serum abrogated the miR-27b’s effects on lipid accumulation (*p* < 0.05).

### 3.6 LGZG-containing serum restored mitochondrial function in miR-27b overexpressed adipocytes

**FIGURE 6:**
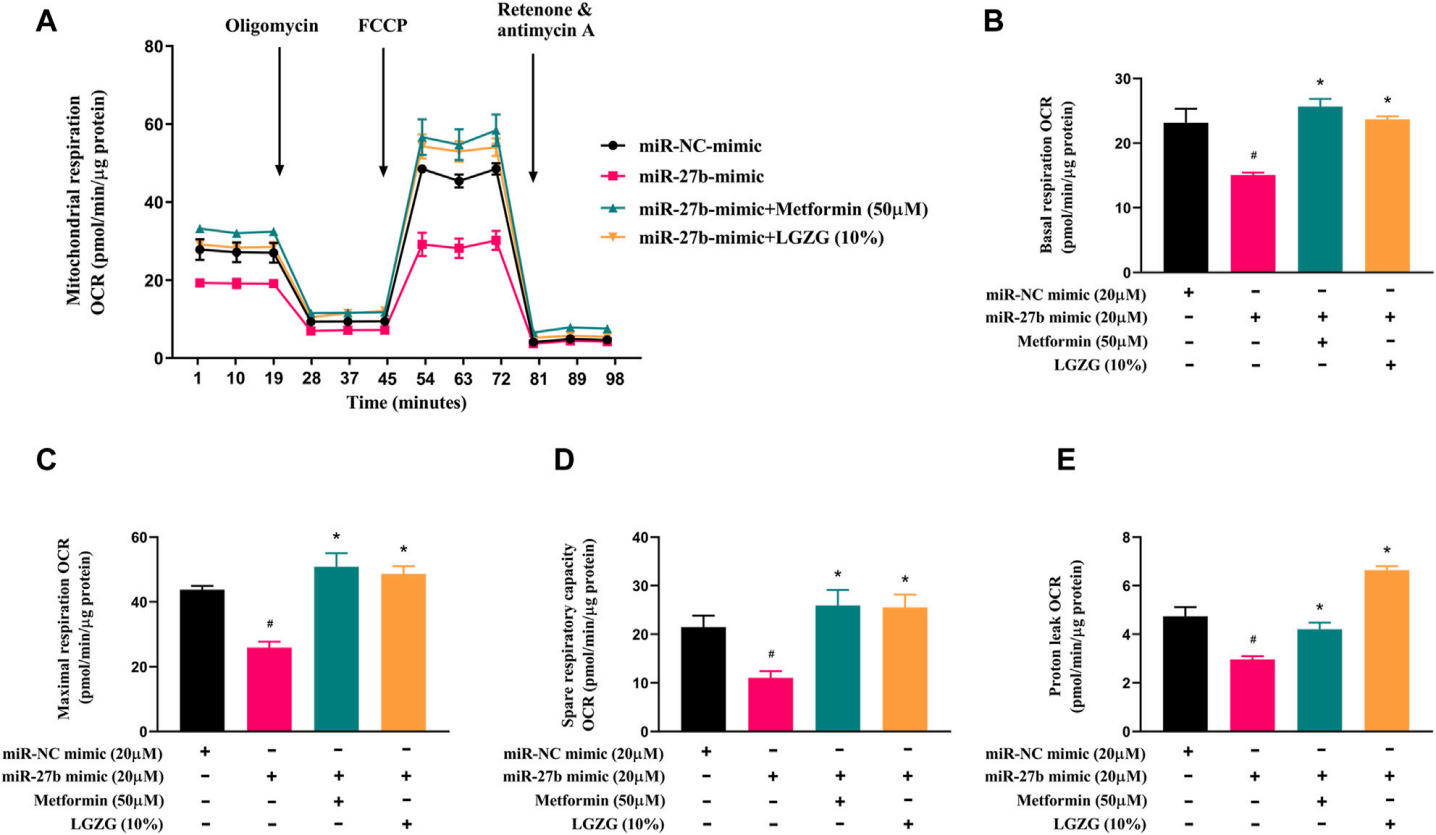
The effect of LGZG-containing serum on mitochondrial respiratory rate in response to overexpressed miR-27b. **(A)** The line chart for mitochondrial oxygen consumption rate (OCR) plotted under different substrates at the indicated time points. **(B)** Basal respiration OCR, **(C)** maximal respiration OCR, **(D)** spare respiratory capacity, and **(E)** proton leak OCR of 3T3-L1 adipocytes after transfection with miR-27b mimic or miR-NC mimic for 48 h. All data were presented as mean ± SEM, with *n* = 3 for each group in **(A–E)**. #*p* < 0.05 versus the negative control adipocytes; *p < 0.05 versus the miR-27b overexpressed adipocytes.

Subsequently, we explored the effect of LGZG-containing serum on mitochondrial respiration in miR-27b overexpressed adipocytes. Mitochondrial stress assay indicated miR-27b overexpression reduced basal respiration, maximal respiration, and spare respiratory capacity, along with oxygen consumption for proton leak than that of controls (Figures 6A–E, *p* < 0.05). However, LGZG-containing serum treatment not only counteracted the suppressive effect of miR-27b mitochondrial respiratory and thermogenic function, but also restored mitochondrial activity to levels observed in the negative control adipocytes, effectively reversing miR-27b’s inhibition on mitochondrial respiratory and thermogenic function (*p* < 0.05).

## 4 Discussion

Manipulating white adipocytes to convert into brown-like adipocytes offers a promising solution for obesity, due to increased energy expenditure and heat production (Altınova, 2022). Numerous studies have been conducted to investigate TCM resources for white fat browning induction (Chen et al., 2021; Ma et al., 2022). Our study successfully verified that LGZG, a traditional Chinese medicine formula renowned for its beneficial effects in obesity and other metabolic diseases, could stimulate 3T3-L1 adipocytes browning, and the regulation of miR-27b/PRDM16 signaling pathway may be linked to this browning effect.

Lipid accumulation is closely related to the occurrence and development of obesity (Oh et al., 2023). In this study, we found that LGZG-containing serum significantly reduced lipid accumulation in 3T3-L1 adipocytes, resulting in a decrease in lipid droplet size in a dose-dependent manner without inhibitory effect on cell viability, along with a possible increase in smaller lipid droplet count. This aligns with the changes in morphological characteristics of adipocytes browning (Wang and Seale, 2016). Normally, the majority of excess energy is stored as TGs in lipid droplets of white fat. High TGs levels are an important marker of obesity and its associated metabolic disorders, contributing to decreased high-density lipoprotein and increased blood glucose and blood pressure (Quispe et al., 2016; Kim et al., 2021). Our results showed a decreased TG content both in and out of the cells with LGZG-containing serum administration, consistent with the results of oil red o staining.

Mitochondrial respiratory function acts a pivotal part in modulating energy metabolism and expenditure in thermogenic adipocytes. The OCR parameters are commonly used to assess mitochondrial activity of cells in real time (Vásquez-Reyes et al., 2021). Here we showed that LGZG-containing serum boosted basal respiration, maximal respiration, and the spare respiratory in 3T3-L1 adipocytes, denoting that LGZG-containing serum enhanced basal metabolism of adipocytes, along with metabolic flexibility responding to stress conditions. Moreover, LGZG-containing serum increased mitochondrial proton leak, which is an important sign of adipocytes thermogenesis and mediated by UCP1 (Bertholet and Kirichok, 2022). This protein, residing in the inner membrane of mitochondria, facilitates proton leakage to uncouple the oxidative phosphorylation pathway from ATP synthesis, thus dissipating energy in the form of heat instead of ATP production (Nicholls, 2021). Our results clearly indicated that the enhanced mitochondrial uncoupling exhibited in LGZG-containing serum exposure was in line with higher UCP1 expression.

The anti-obesity effect associated with fat browning hingers on activating of thermogenic programming. In this study, 3T3-L1 adipocytes exposed to LGZG-containing serum exhibited significantly reduced miR-27b expression, accompanied by an elevation of browning markers, such as UCP1, PRDM16, PGC-1α, PPARγ, CTBP1, and CTBP2 at mRNA and protein levels. MiR-27b, an important upstream regulator of signal transduction cascade involved in adipocytes browning (Lin et al., 2009). Further research found that miR-27b prevented WAT browning by directly targeting PRDM16 (Kong et al., 2015). Notably, PRDM16, a molecular switch for the development of brown and white adipocytes, enhanced energy dissipation through forming a functional complex with PGC-1α and PPARγ to increase the expression of UCP1. PRDM16 also complexes with CTBP1 and CTBP2, pivotal regulators of brown-like phenotype, to inhibit white fat development (Kajimura et al., 2008; Peng et al., 2021; Wang et al., 2022). PGC-1α has been described as an essential thermogenic factor for fat browning to its activation of UCP1, and beyond, it also interacts with PPARγ to regulate mitochondrial biogenesis and enhance metabolic capacity (Kong et al., 2022). Integrating the mitochondrial OCR data, we concluded that the enhancement of mitochondrial activity by LGZG-containing serum may act through the PRDM16/PGC-1α/PPARγ pathway.

To further confirm that miR-27b is an underlying target for the browning effect by LGZG-containing serum, we overexpressed miR-27b in 3T3-L1 adipocytes. MiR-27b overexpression was negatively correlated with the expression of UCP1, PRDM16, PGC-1α, PPARγ, CTBP1, and CTBP2. In contrast, LGZG-containing serum completely reversed the miR-27b’s inhibitory effect on PRDM16-mediated browning program. Furthermore, miR-27b was confirmed to cause unfavorable effects on lipid accumulation and energy consumption (Kong et al., 2015), manifesting as decreased oxygen consumption and heat release, which was essentially in line with our findings. However, LGZG-containing serum reversed these effects of overexpressed miR-27b. These reversals highlight that miR-27b/PRDM16 axis may involve in the LGZG-containing serum-induced white fat browning, at least *in vitro*. However, further *in vivo* studies are necessary to fully understand the effect of LGZG, which we plan to explore in our future research.

Furthermore, using LGZG-containing serum obtained by oral administration of LGZG to animals to treat adipocytes *in vitro* can better reflect the actual pharmacological effects of LGZG on local tissues. This is because LGZG-containing serum not only contains the original components of LGZG but also includes certain metabolites produced *in vivo*. In fact, 49 chemical compounds were identified in LGZG-containing serum using UHPLC-Q-Orbitrap HRMS, including 11 flavonoids, 9 isoflavonoids, 2 neoflavonoids, 17 triterpenoids, 2 bile acids, alcohols and derivatives, 1 chalcones and dihydrochalcones, 4 terpene glycosides, 2 terpene lactones, 1 naphthofurans. Some of these compounds have functions of regulating lipid metabolism, reducing fat accumulation, and improving metabolic health. For example, polyporenic acid C significantly inhibited lipid droplet deposition in hepatocytes and decreased levels of total cholesterol and TG (He et al., 2022). Atractylenolides are promising compounds for reducing the blood glucose and blood lipid levels. Specifically, atractylenolide II has been validated to improve obesity-induced hyperlipidemia, hepatosteatosis, and IR while simultaneously enhancing weight loss through activating adenosine 5’-monophosphate-activated protein kinase (AMPK)/PGC-1α/UCP1 axis (Liu et al., 2022). Atractylenolide III could boost the thermogenic function of BAT, promote the WAT browning, and increase energy expenditure via activation of silence information regulator T1 (SIRT1)/PGC-1α signaling axis (Zheng et al., 2023). The advantages of these compounds in metabolism greatly enhance LGZG’s application value in combating obesity and improving metabolic health.

**FIGURE 7:**
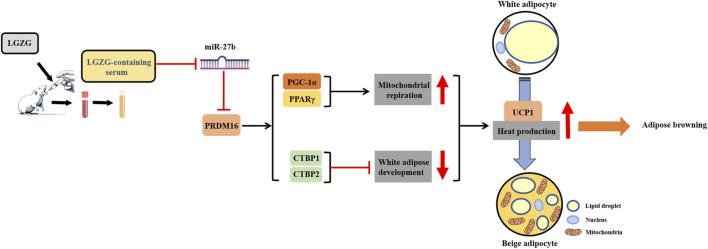
Schematic diagram of LGZG’s promoting 3T3-L1 adipocytes browning via stimulating miR-27b/PRDM16 signaling pathway.

In conclusion, this study exhibited that LGZG-containing serum promoted adipocytes browning in 3T3-L1 cells, primarily manifesting as decreased lipid droplet accumulation and enhanced mitochondrial function, including mitochondrial basal, maximal, and spare respiratory capacity, and mitochondrial uncoupling. And it firstly unveils that this effect may rely on the activation of the miR-27b/PRDM16 signaling pathway (Figure 7). The experimental evidence provided in the current study not only establishes a pivotal role of LGZG in regulating thermogenesis and browning, but also lays groundwork for future *in vivo* and clinical research on its obesity-treating properties.

## Data Availability

The original contributions presented in the study are included in the article/Supplementary Material, further inquiries can be directed to the corresponding author/s.

## References

[B1] Altınova A. E. (2022). Beige adipocyte as the flame of white adipose tissue: regulation of browning and impact of obesity. J. Clin. Endocrinol. Metab. 107 (5), e1778–e1788. 10.1210/clinem/dgab921 34967396

[B2] Bertholet A. M. Kirichok Y. (2022). Mitochondrial H(+) leak and thermogenesis. Annu. Rev. Physiol. 84, 381–407. 10.1146/annurev-physiol-021119-034405 34758268 PMC8976115

[B3] Chen C. C. Kuo C. H. Leu Y. L. Wang S. H. (2021). Corylin reduces obesity and insulin resistance and promotes adipose tissue browning through SIRT-1 and β3-AR activation. Pharmacol. Res. 164, 105291. 10.1016/j.phrs.2020.105291 33253817

[B4] Choi J. W. Choi H. J. Ryu G. H. Lee J. W. Beak J. K. Koh E. J. (2023). Paeonia lactiflora root decreases lipid accumulation through the induction of lipolysis and thermogenesis via AMPK activation in 3T3-L1 cells. Int. J. Mol. Med. 52 (2), 65. 10.3892/ijmm.2023.5268 37326061 PMC10555475

[B5] Chouchani E. T. Kazak L. Spiegelman B. M. (2019). New advances in adaptive thermogenesis: UCP1 and beyond. Cell Metab. 29 (1), 27–37. 10.1016/j.cmet.2018.11.002 30503034

[B6] Fang S. Suh J. M. Reilly S. M. Yu E. Osborn O. Lackey D. (2015). Intestinal FXR agonism promotes adipose tissue browning and reduces obesity and insulin resistance. Nat. Med. 21 (2), 159–165. 10.1038/nm.3760 25559344 PMC4320010

[B7] Gan C. C. Ni T. W. Yu Y. Qin N. Chen Y. Jin M. N. (2017). Flavonoid derivative (Fla-CN) inhibited adipocyte differentiation via activating AMPK and up-regulating microRNA-27 in 3T3-L1 cells. Eur. J. Pharmacol. 797, 45–52. 10.1016/j.ejphar.2017.01.009 28088385

[B8] Han J. Zhang H. Zhang Y. Zhang Z. Yu M. Wang S. (2022). Lingguizhugan decoction protects PC12 cells against Aβ(25-35)-induced oxidative stress and neuroinflammation by modulating NF-κB/MAPK signaling pathways. J. Ethnopharmacol. 292, 115194. 10.1016/j.jep.2022.115194 35304276

[B9] He J. Yang Y. Zhang F. Li Y. Li X. Pu X. (2022). Effects of Poria cocos extract on metabolic dysfunction-associated fatty liver disease via the FXR/PPARα-SREBPs pathway. Front. Pharmacol. 13, 1007274. 10.3389/fphar.2022.1007274 36278226 PMC9581278

[B10] Ikeda K. Maretich P. Kajimura S. (2018). The common and distinct features of Brown and beige adipocytes. Trends Endocrinol. Metab. 29 (3), 191–200. 10.1016/j.tem.2018.01.001 29366777 PMC5826798

[B11] Ikeda K. Yamada T. (2020). UCP1 dependent and independent thermogenesis in Brown and beige adipocytes. Front. Endocrinol. (Lausanne) 11, 498. 10.3389/fendo.2020.00498 32849287 PMC7399049

[B12] Irfan H. (2024). Obesity, cardiovascular disease, and the promising role of semaglutide: insights from the SELECT trial. Curr. Probl. Cardiol. 49 (1 Pt A), 102060. 10.1016/j.cpcardiol.2023.102060 37640171

[B13] Jastroch M. Seebacher F. (2020). Importance of adipocyte browning in the evolution of endothermy. Philos. Trans. R. Soc. Lond B Biol. Sci. 375 (1793), 20190134. 10.1098/rstb.2019.0134 31928187 PMC7017435

[B14] Kajimura S. Seale P. Tomaru T. Erdjument-Bromage H. Cooper M. P. Ruas J. L. (2008). Regulation of the brown and white fat gene programs through a PRDM16/CtBP transcriptional complex. Genes Dev. 22 (10), 1397–1409. 10.1101/gad.1666108 18483224 PMC2377193

[B15] Kim H. S. Lee J. Cho Y. K. Kim E. H. Lee M. J. Kim H. K. (2021). Prognostic value of triglyceride and glucose index for incident type 2 diabetes beyond metabolic health and obesity. Endocrinol. Metab. Seoul. 36 (5), 1042–1054. 10.3803/EnM.2021.1184 34674505 PMC8566137

[B16] Kim N. Y. Lim C. M. Park H. M. Kim J. Pham T. H. Yang Y. (2022). MMPP promotes adipogenesis and glucose uptake via binding to the PPARγ ligand binding domain in 3T3-L1 MBX cells. Front. Pharmacol. 13, 994584. 10.3389/fphar.2022.994584 36339572 PMC9634037

[B17] Kong S. Cai B. Nie Q. (2022). PGC-1α affects skeletal muscle and adipose tissue development by regulating mitochondrial biogenesis. Mol. Genet. Genomics 297 (3), 621–633. 10.1007/s00438-022-01878-2 35290519

[B18] Kong X. Yu J. Bi J. Qi H. Di W. Wu L. (2015). Glucocorticoids transcriptionally regulate miR-27b expression promoting body fat accumulation via suppressing the browning of white adipose tissue. Diabetes 64 (2), 393–404. 10.2337/db14-0395 25187367 PMC4876791

[B19] Li B. Fan S. Hu J. Ma Y. Feng Y. Wang F. (2021). Phytochemical analysis using UPLC-MS/MS combined with network pharmacology methods to explore the biomarkers for the quality control of lingguizhugan decoction. Evid. Based Complement. Altern. Med. 2021, 7849032. 10.1155/2021/7849032 PMC8716202 34976099

[B20] Li J. Wang X. Meng X. Zhou X. Huang H. Feng Y. (2023a). Geraniin targeting CaMKK2 inhibits lipid accumulation in 3T3-L1 adipocytes by suppressing lipogenesis. Chem. Biol. Interact. 372, 110364. 10.1016/j.cbi.2023.110364 36706894

[B21] Li Q. Wu R. Guo F. An R. Shi B. (2023b). Clinical effect of modified Linggui Zhugan decoction in the treatment of obese type 2 diabetes mellitus. Jilin J. Chin. Med. 43 (07), 784–788. 10.13463/j.cnki.jlzyy.2023.07.010

[B22] Li S. S. Li B. Liu S. H. Li B. Dong Y. Gao J. (2018). Research on ancient literature of Lingguizhugan Decoction, a classical prescription. Zhonghua Yi Shi Za Zhi 48 (1), 17–20. 10.3760/cma.j.issn.0255-7053.2018.01.004 29886697

[B23] Li W. Xiao H. (2021). Dihydromyricetin alleviates high glucose-induced oxidative stress and apoptosis in human retinal pigment epithelial cells by downregulating miR-34a expression. Diabetes Metab. Syndr. Obes. 14, 387–397. 10.2147/dmso.S290633 33536772 PMC7850407

[B24] Li Y. Li C. Wu J. Liu W. Li D. Xu J. (2020). Harmane ameliorates obesity though inhibiting lipid accumulation and inducing adipocyte browning. RSC Adv. 10 (8), 4397–4403. 10.1039/c9ra09383d 35495252 PMC9049078

[B25] Li Y. Wang D. Ping X. Zhang Y. Zhang T. Wang L. (2022). Local hyperthermia therapy induces browning of white fat and treats obesity. Cell 185 (6), 949–966.e19. 10.1016/j.cell.2022.02.004 35247329

[B26] Lin Q. Gao Z. Alarcon R. M. Ye J. Yun Z. (2009). A role of miR-27 in the regulation of adipogenesis. Febs J. 276 (8), 2348–2358. 10.1111/j.1742-4658.2009.06967.x 19348006 PMC5330386

[B27] Liu X. Huang Y. Liang X. Wu Q. Wang N. Zhou L. J. (2022). Atractylenolide III from Atractylodes macrocephala Koidz promotes the activation of brown and white adipose tissue through SIRT1/PGC-1α signaling pathway. Phytomedicine 104, 154289. 10.1016/j.phymed.2022.154289 35785561

[B28] Luo L. Liu C. Huang Y. Zhang Z. Yu Y. Long S. (2021). Clinical effect of modified Linggui Zhugan Decoction combined with Metformin in the treatment of obesity type 2 diabetes mellitus with phlegmdampness syndrome. China Med. Her. 18 (33), 127–130.

[B29] Ma P. Y. Li X. Y. Wang Y. L. Lang D. Q. Liu L. Yi Y. K. (2022). Natural bioactive constituents from herbs and nutraceuticals promote browning of white adipose tissue. Pharmacol. Res. 178, 106175. 10.1016/j.phrs.2022.106175 35283301

[B30] Mehlem A. Hagberg C. E. Muhl L. Eriksson U. Falkevall A. (2013). Imaging of neutral lipids by oil red O for analyzing the metabolic status in health and disease. Nat. Protoc. 8 (6), 1149–1154. 10.1038/nprot.2013.055 23702831

[B31] Nasci V. L. Chuppa S. Griswold L. Goodreau K. A. Dash R. K. Kriegel A. J. (2019). miR-21-5p regulates mitochondrial respiration and lipid content in H9C2 cells. Am. J. Physiol. Heart Circ. Physiol. 316 (3), H710–h721. 10.1152/ajpheart.00538.2017 30657727 PMC6459316

[B32] Nicholls D. G. (2021). Mitochondrial proton leaks and uncoupling proteins. Biochim. Biophys. Acta Bioenerg. 1862 (7), 148428. 10.1016/j.bbabio.2021.148428 33798544

[B33] Ning Y. Gong Y. Zheng T. Xie Y. Yuan S. Ding W. (2022). Lingguizhugan decoction targets intestinal microbiota and metabolites to reduce insulin resistance in high-fat diet rats. Diabetes Metab. Syndr. Obes. 15, 2427–2442. 10.2147/dmso.S370492 35971521 PMC9375570

[B34] Oh J. Ahn S. Zhou X. Lim Y. J. Hong S. Kim H. S. (2023). Effects of cinnamon (cinnamomum zeylanicum) extract on adipocyte differentiation in 3T3-L1 cells and lipid accumulation in mice fed a high-fat diet. Nutrients 15 (24), 5110. 10.3390/nu15245110 38140369 PMC10745629

[B35] Pacifici F. Malatesta G. Mammi C. Pastore D. Marzolla V. Ricordi C. (2023). A novel mix of polyphenols and micronutrients reduces adipogenesis and promotes white adipose tissue browning via UCP1 expression and AMPK activation. Cells 12 (5), 714. 10.3390/cells12050714 36899850 PMC10001138

[B36] Peng W. Q. Xiao G. Li B. Y. Guo Y. Y. Guo L. Tang Q. Q. (2021). l-Theanine activates the browning of white adipose tissue through the AMPK/α-Ketoglutarate/Prdm16 Axis and ameliorates diet-induced obesity in mice. Diabetes 70 (7), 1458–1472. 10.2337/db20-1210 33863801

[B37] Polyzos S. A. Kountouras J. Mantzoros C. S. (2019). Obesity and nonalcoholic fatty liver disease: from pathophysiology to therapeutics. Metabolism 92, 82–97. 10.1016/j.metabol.2018.11.014 30502373

[B38] Price N. L. Fernández-Hernando C. (2016). miRNA regulation of white and brown adipose tissue differentiation and function. Biochim. Biophys. Acta 1861 (12 Pt B), 2104–2110. 10.1016/j.bbalip.2016.02.010 26898181 PMC4987264

[B39] Quispe R. Martin S. S. Jones S. R. (2016). Triglycerides to high-density lipoprotein-cholesterol ratio, glycemic control and cardiovascular risk in obese patients with type 2 diabetes. Curr. Opin. Endocrinol. Diabetes Obes. 23 (2), 150–156. 10.1097/med.0000000000000241 26863278

[B40] Ruze R. Liu T. Zou X. Song J. Chen Y. Xu R. (2023). Obesity and type 2 diabetes mellitus: connections in epidemiology, pathogenesis, and treatments. Front. Endocrinol. (Lausanne) 14, 1161521. 10.3389/fendo.2023.1161521 37152942 PMC10161731

[B41] Sakers A. De Siqueira M. K. Seale P. Villanueva C. J. (2022). Adipose-tissue plasticity in health and disease. Cell 185 (3), 419–446. 10.1016/j.cell.2021.12.016 35120662 PMC11152570

[B42] Schirinzi V. Poli C. Berteotti C. Leone A. (2023). Browning of adipocytes: a potential therapeutic approach to obesity. Nutrients 15 (9), 2229. 10.3390/nu15092229 37432449 PMC10181235

[B43] Sciandra F. Bottoni P. De Leo M. Braca A. Brancaccio A. Bozzi M. (2023). Verbascoside elicits its beneficial effects by enhancing mitochondrial spare respiratory capacity and the Nrf2/HO-1 mediated antioxidant system in a murine skeletal muscle cell line. Int. J. Mol. Sci. 24 (20), 15276. 10.3390/ijms242015276 37894956 PMC10607197

[B44] Smolina N. Bruton J. Kostareva A. Sejersen T. (2017). Assaying mitochondrial respiration as an indicator of cellular metabolism and fitness. Methods Mol. Biol. 1601, 79–87. 10.1007/978-1-4939-6960-9_7 28470519

[B45] Sun L. Trajkovski M. (2014). MiR-27 orchestrates the transcriptional regulation of brown adipogenesis. Metabolism 63 (2), 272–282. 10.1016/j.metabol.2013.10.004 24238035

[B46] Sun Y. N. Yang Z. X. Ren F. Z. Fang B. (2020). FGF19 alleviates palmitate-induced atrophy in C2C12 cells by inhibiting mitochondrial overload and insulin resistance. Int. J. Biol. Macromol. 158, 401–407. 10.1016/j.ijbiomac.2020.04.186 32344084

[B47] Tu J. Zhu S. Li B. Xu G. Luo X. Jiang L. (2020). Gegen qinlian decoction coordinately regulates PPARγ and PPARα to improve glucose and lipid homeostasis in diabetic rats and insulin resistance 3T3-L1 adipocytes. Front. Pharmacol. 11, 811. 10.3389/fphar.2020.00811 32595495 PMC7300300

[B48] Vásquez-Reyes S. Velázquez-Villegas L. A. Vargas-Castillo A. Noriega L. G. Torres N. Tovar A. R. (2021). Dietary bioactive compounds as modulators of mitochondrial function. J. Nutr. Biochem. 96, 108768. 10.1016/j.jnutbio.2021.108768 34000412

[B49] Wang Q. Li H. Tajima K. Verkerke A. R. P. Taxin Z. H. Hou Z. (2022). Post-translational control of beige fat biogenesis by PRDM16 stabilization. Nature 609 (7925), 151–158. 10.1038/s41586-022-05067-4 35978186 PMC9433319

[B50] Wang W. Seale P. (2016). Control of brown and beige fat development. Nat. Rev. Mol. Cell Biol. 17 (11), 691–702. 10.1038/nrm.2016.96 27552974 PMC5627770

[B51] Wu R. Zhao D. An R. Wang Z. Li Y. Shi B. (2019). Linggui zhugan formula improves glucose and lipid levels and alters gut microbiota in high-fat diet-induced diabetic mice. Front. Physiol. 10, 918. 10.3389/fphys.2019.00918 31396097 PMC6663968

[B52] Xu J. Wang R. You S. Zhang L. Zheng P. Ji G. (2020). Traditional Chinese medicine Lingguizhugan decoction treating non-alcoholic fatty liver disease with spleen-yang deficiency pattern: study protocol for a multicenter randomized controlled trial. Trials 21 (1), 512. 10.1186/s13063-020-04362-7 32522273 PMC7288405

[B53] Xu J. Zhang L. Shu G. Wang B. (2019). microRNA-16-5p promotes 3T3-L1 adipocyte differentiation through regulating EPT1. Biochem. Biophys. Res. Commun. 514 (4), 1251–1256. 10.1016/j.bbrc.2019.04.179 31109647

[B54] Yang Y. Li Q. Chen S. Ke B. Huang Y. Qin J. (2014). Effects of modified lingguizhugan decoction combined with weekend fasting on metabolic syndrome. J. Tradit. Chin. Med. 34 (1), 48–51. 10.1016/s0254-6272(14)60053-4 25102690

[B55] Zheng Z. G. Xu Y. Y. Liu W. P. Zhang Y. Zhang C. Liu H. L. (2023). Discovery of a potent allosteric activator of DGKQ that ameliorates obesity-induced insulin resistance via the sn-1,2-DAG-PKCε signaling axis. Cell Metab. 35 (1), 101–117.e11. 10.1016/j.cmet.2022.11.012 36525963

[B56] Ziqubu K. Mazibuko-Mbeje S. E. Mthembu S. X. H. Mabhida S. E. Jack B. U. Nyambuya T. M. (2023). Anti-obesity effects of metformin: a scoping review evaluating the feasibility of Brown adipose tissue as a therapeutic target. Int. J. Mol. Sci. 24 (3), 2227. 10.3390/ijms24032227 36768561 PMC9917329

