# Supplementary figures and images for: Ling-gui-zhu-gan promotes adipocytes browning via targeting the miR-27b/PRDM16 pathway in 3T3-L1 cells
Source: Front Pharmacol. 2024 Aug 14;15:1386794. doi: 10.3389/fphar.2024.1386794 (PMC11349548; doi:10.3389/fphar.2024.1386794)

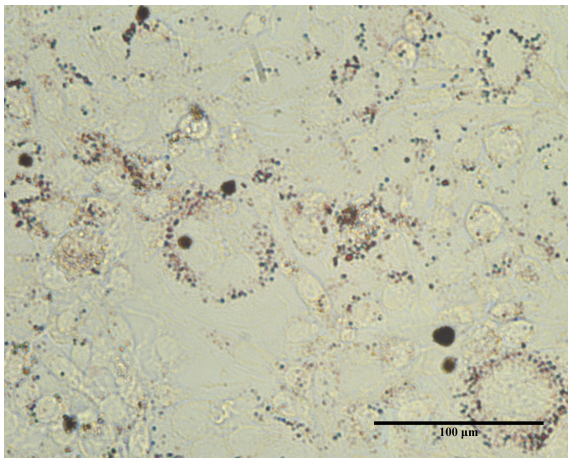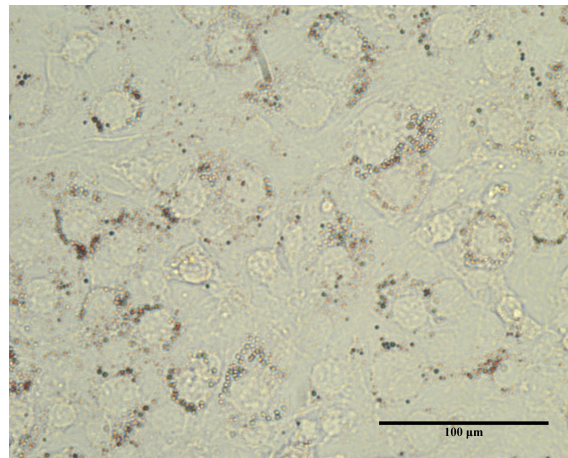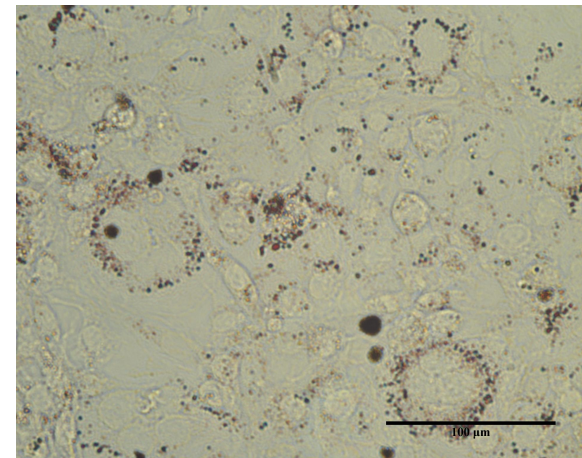

**Figure 5A——miR-NC-mimic (400 x)**

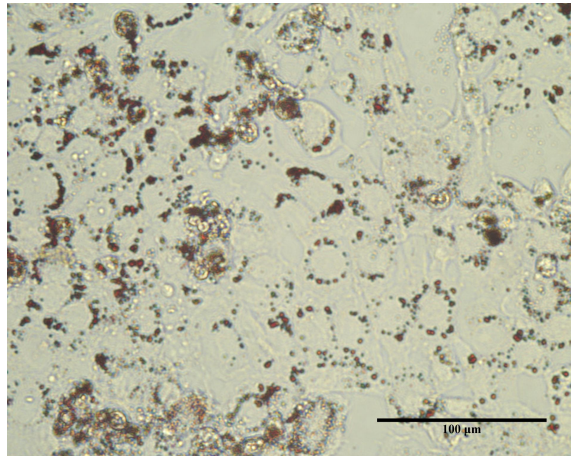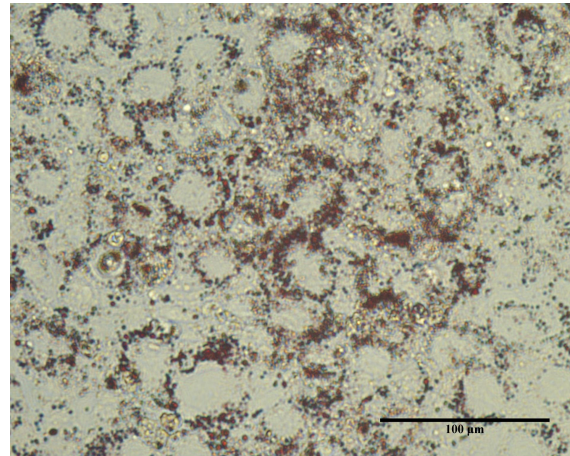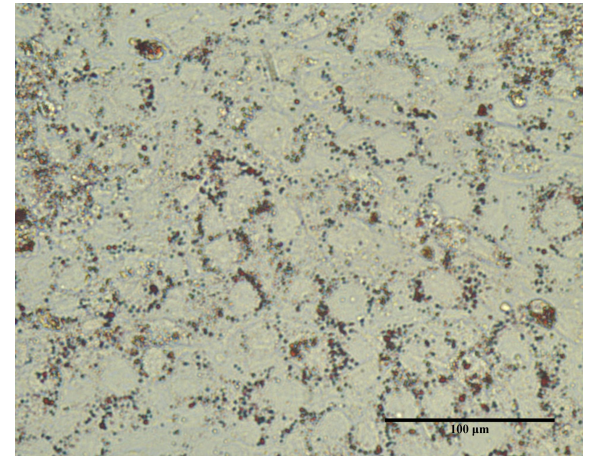

**Figure 5A——miR-27b-mimic (400 x)**

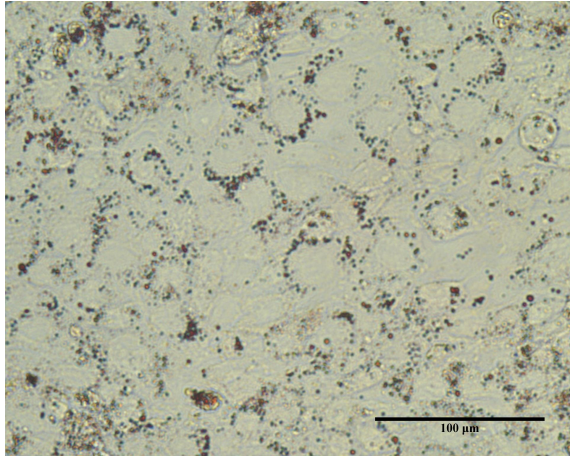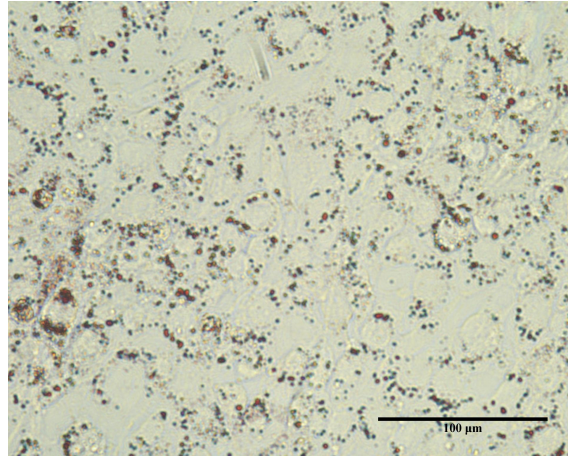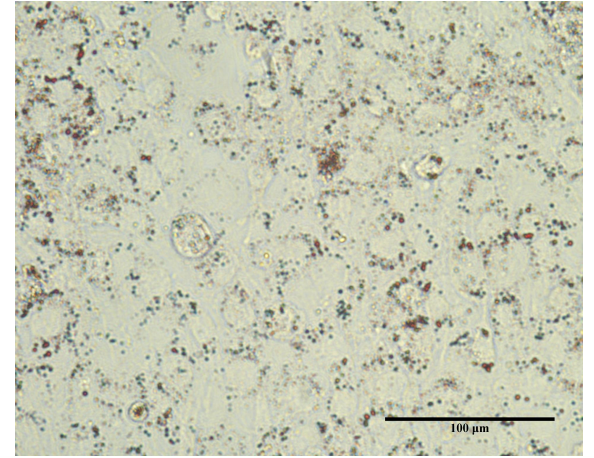

**Figure 5A——metformin (400 x)**

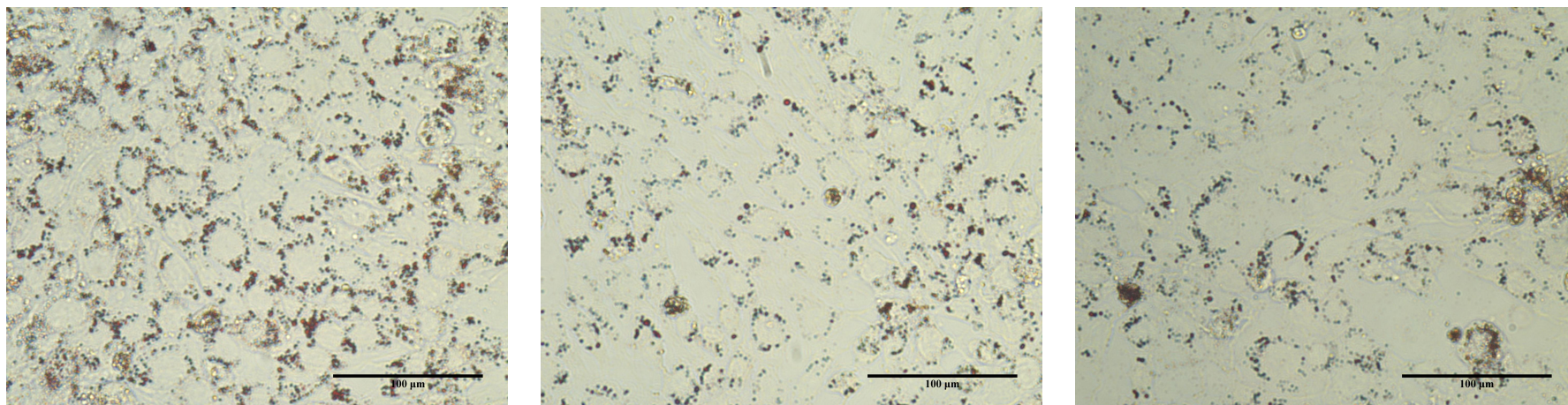

**Figure 5A——LGZG-containing serum (400 x)**

Supplement: Supplementary file 2 [file DataSheet4.PDF]

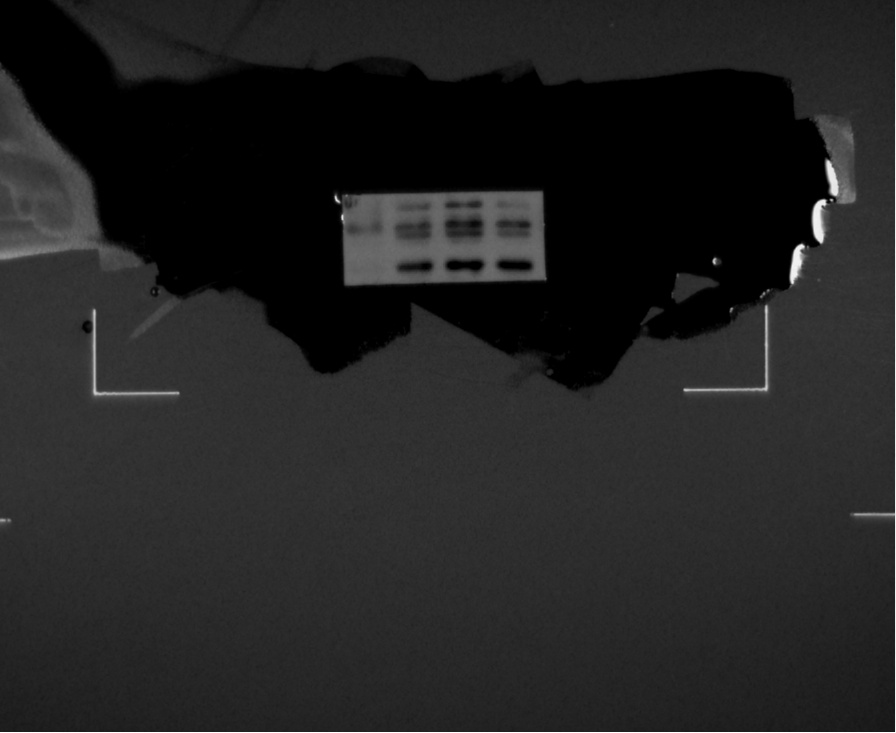

Supplement: Supplementary file 5 [file DataSheet1.ZIP › Supplementary file 2—— Original Image for Figure3(H-M):Western Blot/1.Figure3H—UCP1/Figure3H—UCP1-1.jpg]

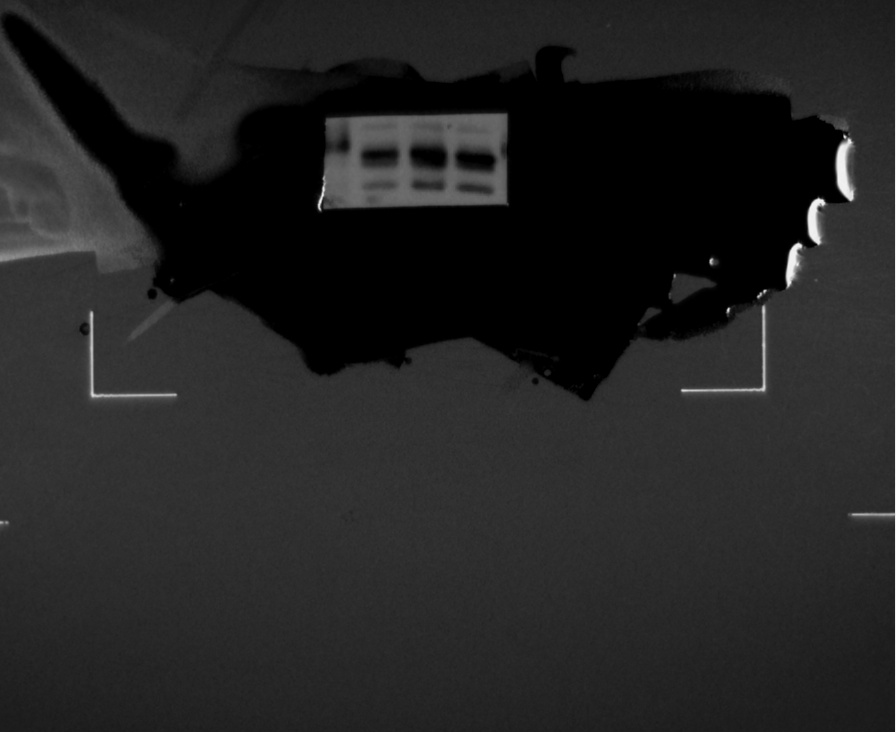

Supplement: Supplementary file 5 [file DataSheet1.ZIP › Supplementary file 2—— Original Image for Figure3(H-M):Western Blot/1.Figure3H—UCP1/Figure3H—UCP1-2.jpg]

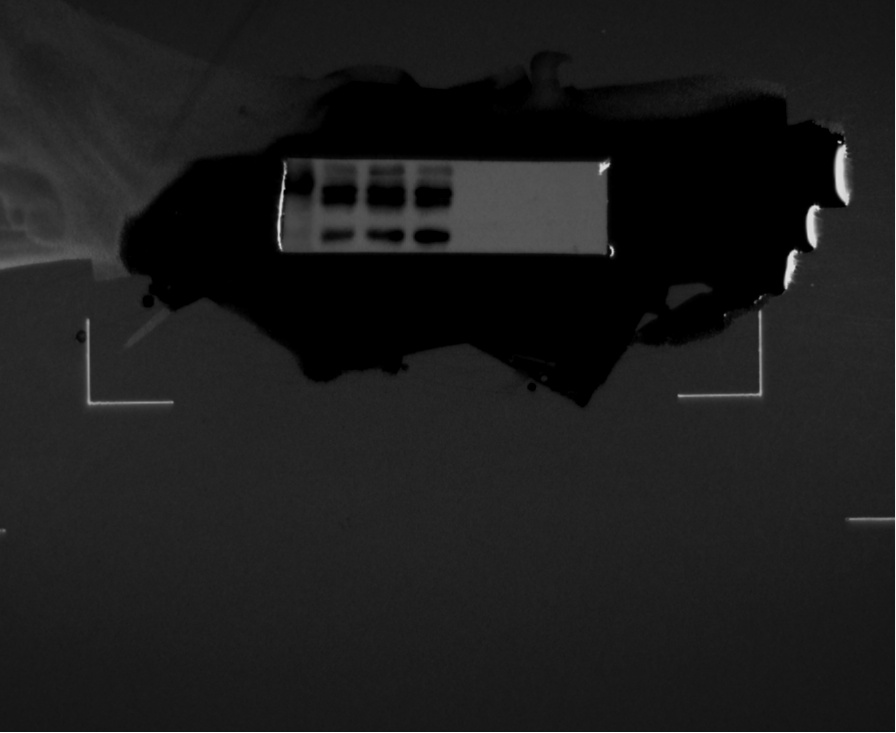

Supplement: Supplementary file 5 [file DataSheet1.ZIP › Supplementary file 2—— Original Image for Figure3(H-M):Western Blot/1.Figure3H—UCP1/Figure3H—UCP1-3.jpg]

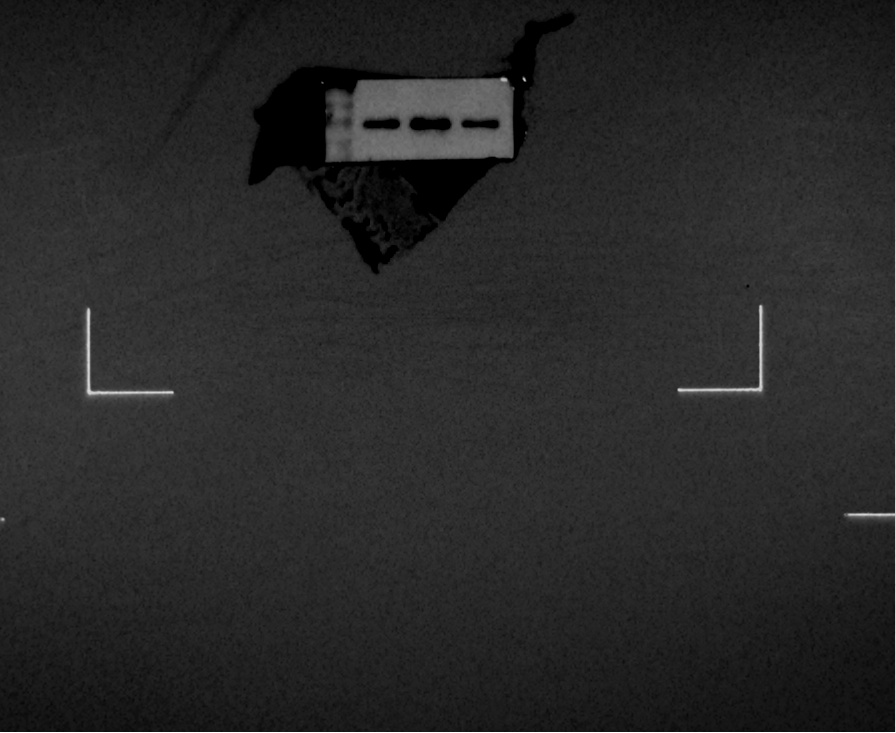

Supplement: Supplementary file 5 [file DataSheet1.ZIP › Supplementary file 2—— Original Image for Figure3(H-M):Western Blot/1.Figure3H—UCP1/Figure3H—β-Tubulin-1.jpg]

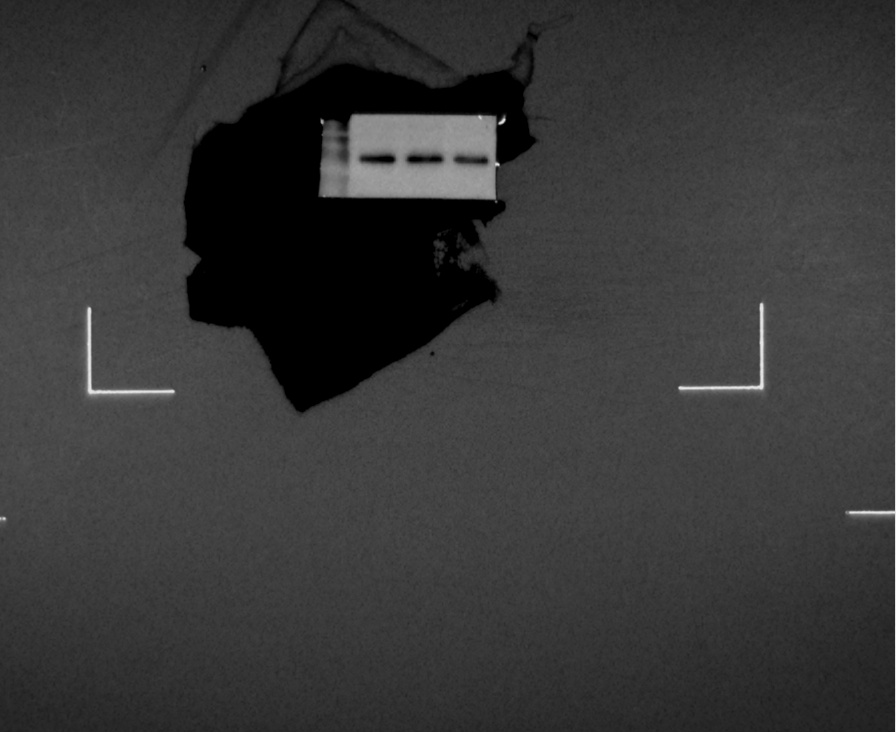

Supplement: Supplementary file 5 [file DataSheet1.ZIP › Supplementary file 2—— Original Image for Figure3(H-M):Western Blot/1.Figure3H—UCP1/Figure3H—β-Tubulin-2.jpg]

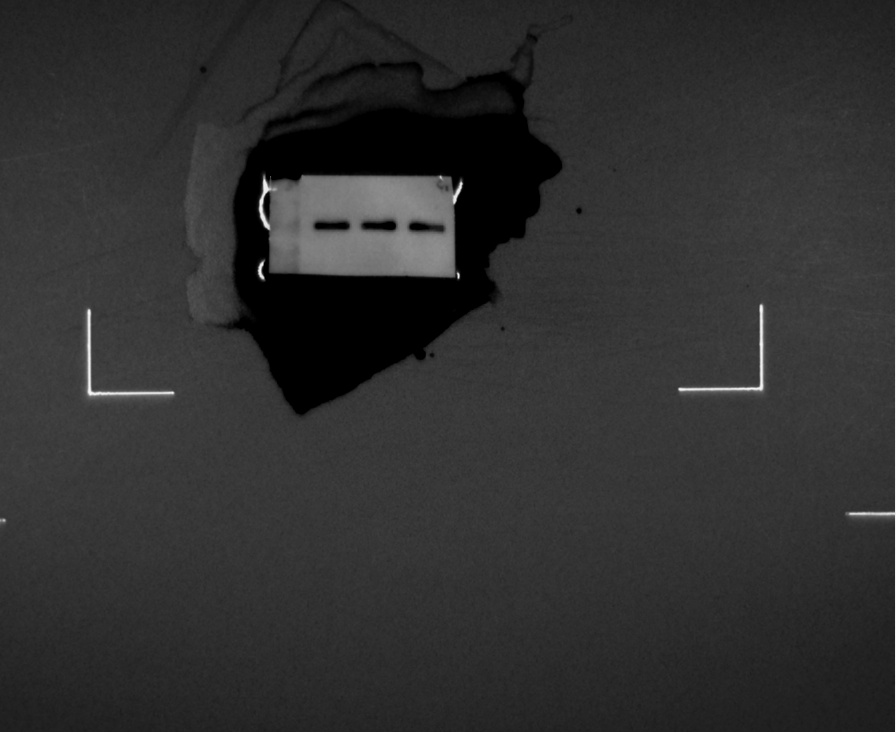

Supplement: Supplementary file 5 [file DataSheet1.ZIP › Supplementary file 2—— Original Image for Figure3(H-M):Western Blot/1.Figure3H—UCP1/Figure3H—β-Tubulin-3.jpg]

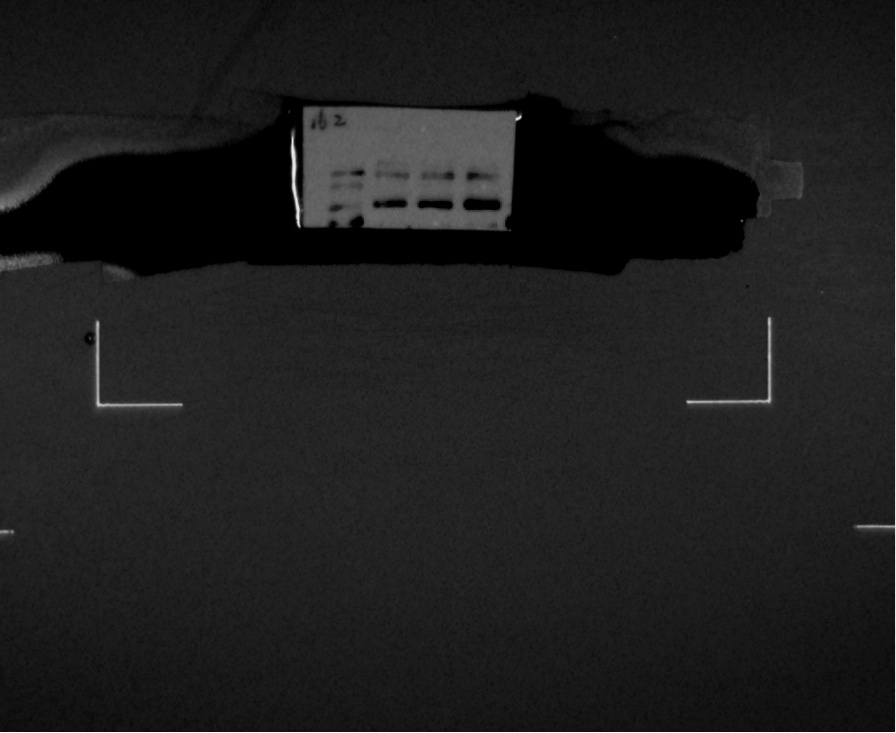

Supplement: Supplementary file 5 [file DataSheet1.ZIP › Supplementary file 2—— Original Image for Figure3(H-M):Western Blot/2.Figure3H—PRDM16/Figure3H—PRDM16-1.jpg]

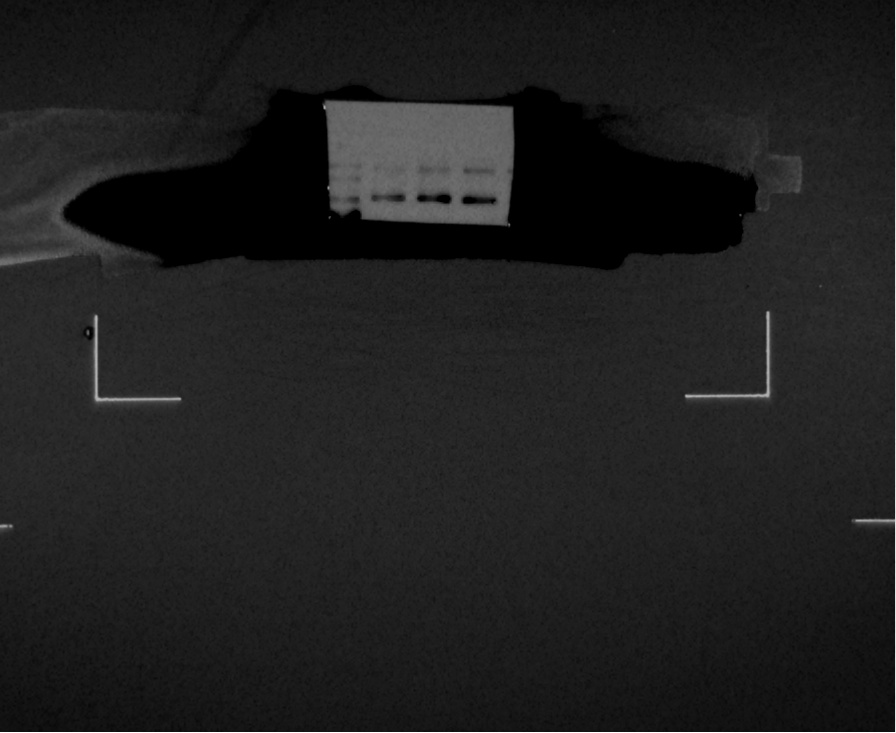

Supplement: Supplementary file 5 [file DataSheet1.ZIP › Supplementary file 2—— Original Image for Figure3(H-M):Western Blot/2.Figure3H—PRDM16/Figure3H—PRDM16-2.jpg]

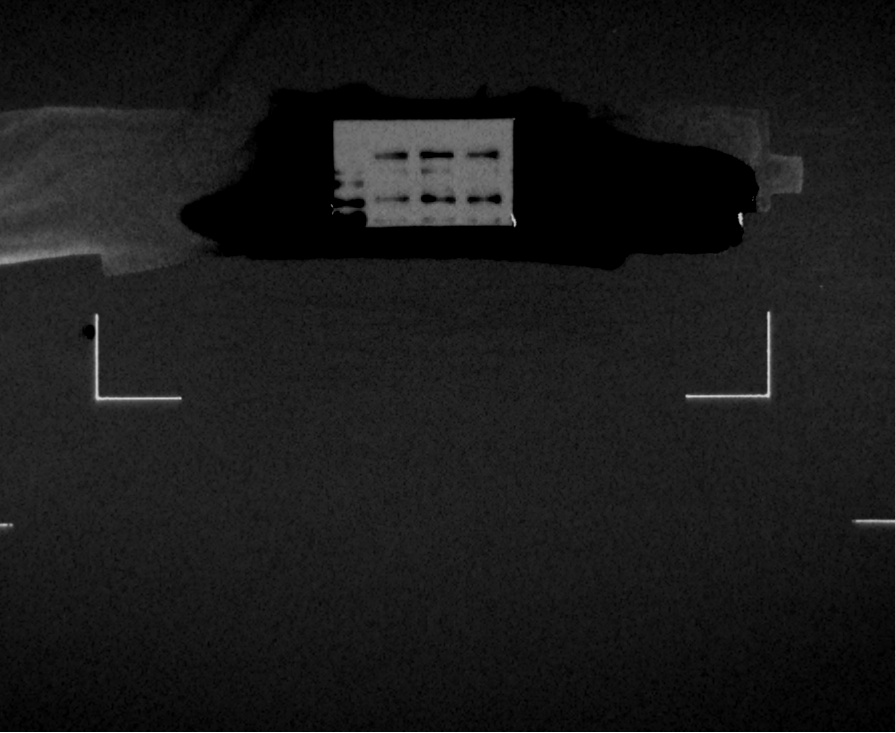

Supplement: Supplementary file 5 [file DataSheet1.ZIP › Supplementary file 2—— Original Image for Figure3(H-M):Western Blot/2.Figure3H—PRDM16/Figure3H—PRDM16-3.jpg]

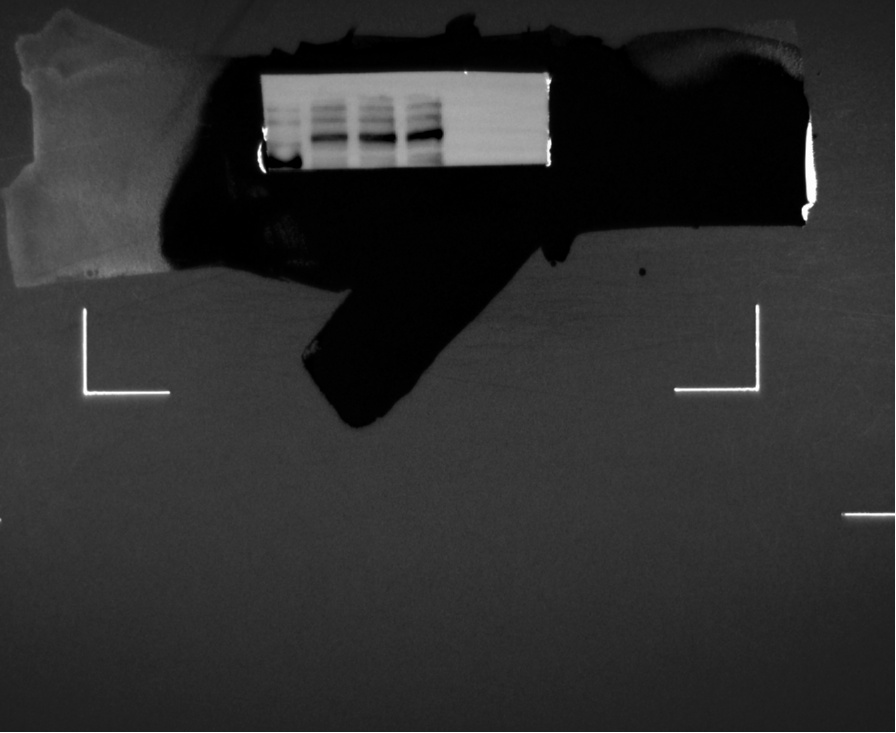

Supplement: Supplementary file 5 [file DataSheet1.ZIP › Supplementary file 2—— Original Image for Figure3(H-M):Western Blot/3.Figure3H—PGC-1α/Figure3H—PGC-1α-1.jpg]

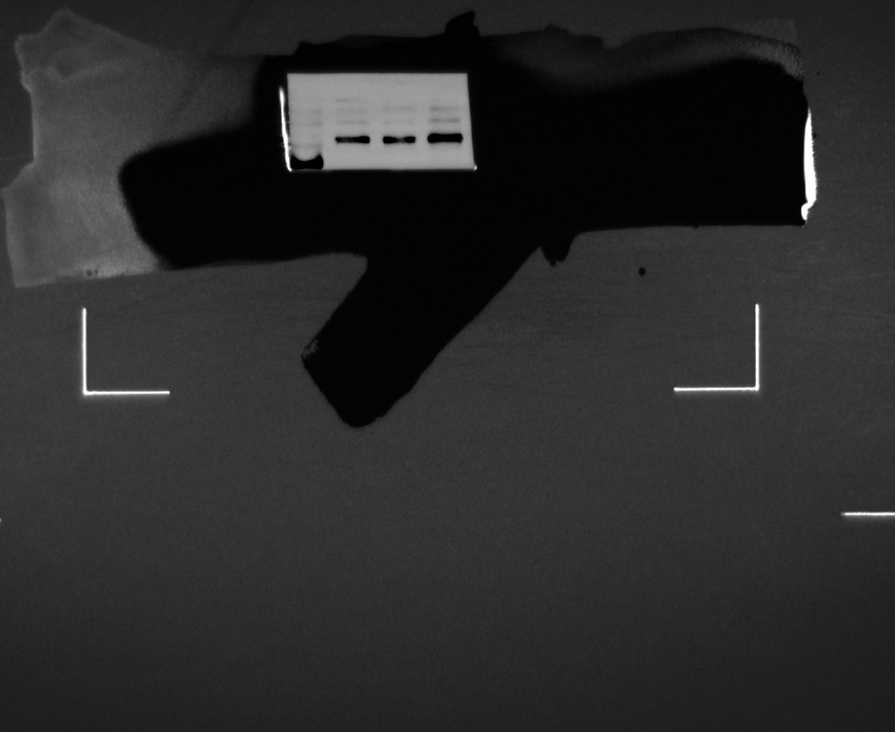

Supplement: Supplementary file 5 [file DataSheet1.ZIP › Supplementary file 2—— Original Image for Figure3(H-M):Western Blot/3.Figure3H—PGC-1α/Figure3H—PGC-1α-2.jpg]

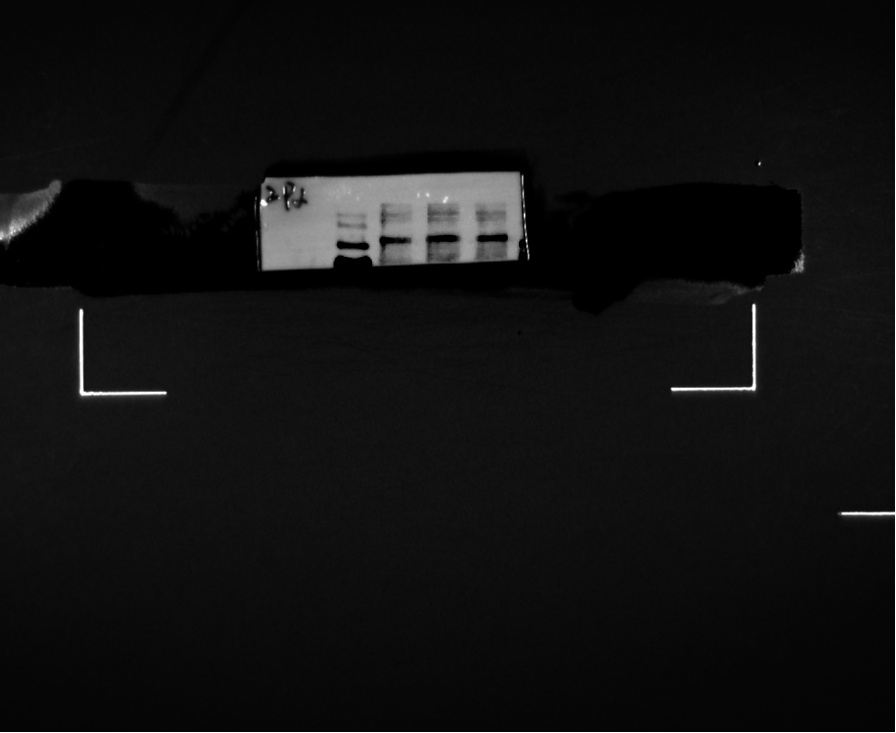

Supplement: Supplementary file 5 [file DataSheet1.ZIP › Supplementary file 2—— Original Image for Figure3(H-M):Western Blot/3.Figure3H—PGC-1α/Figure3H—PGC-1α-3.jpg]

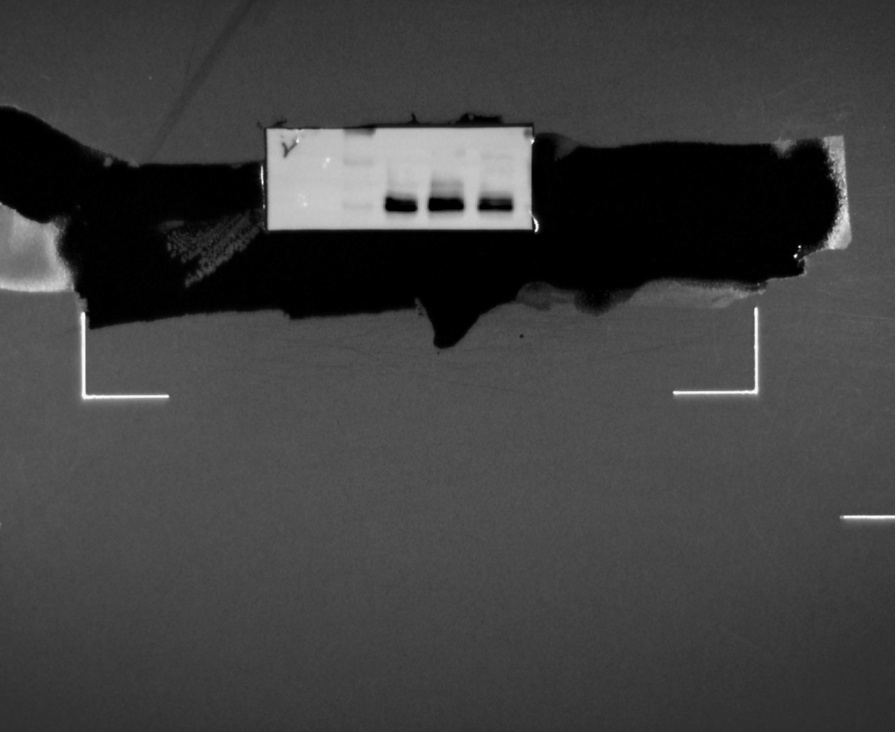

Supplement: Supplementary file 5 [file DataSheet1.ZIP › Supplementary file 2—— Original Image for Figure3(H-M):Western Blot/3.Figure3H—PGC-1α/Figure3H—β-Tubulin-1.jpg]

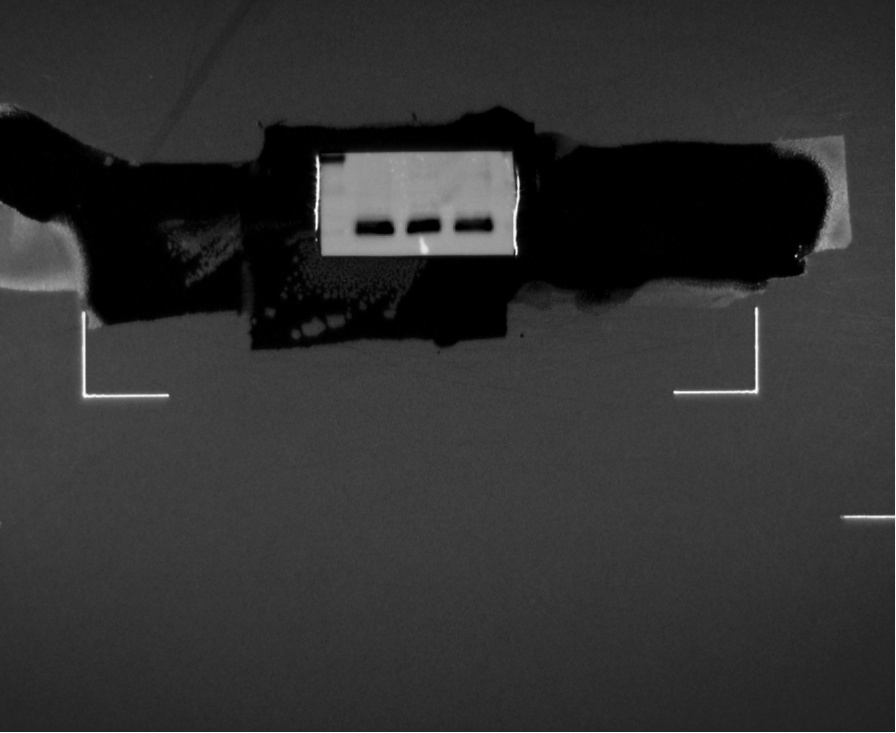

Supplement: Supplementary file 5 [file DataSheet1.ZIP › Supplementary file 2—— Original Image for Figure3(H-M):Western Blot/3.Figure3H—PGC-1α/Figure3H—β-Tubulin-2.jpg]

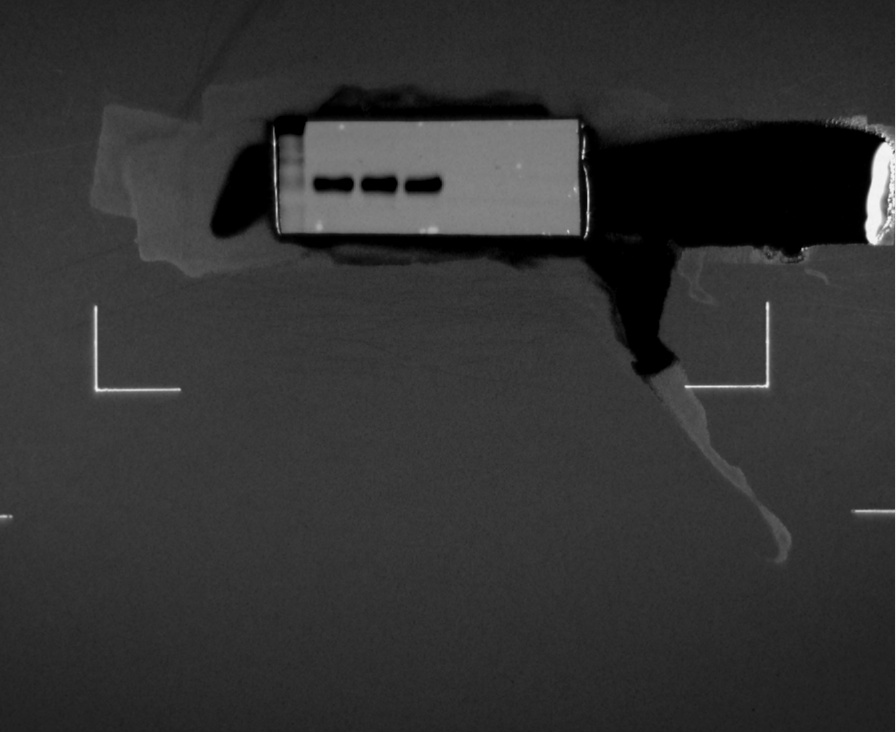

Supplement: Supplementary file 5 [file DataSheet1.ZIP › Supplementary file 2—— Original Image for Figure3(H-M):Western Blot/3.Figure3H—PGC-1α/Figure3H—β-Tubulin-3.jpg]

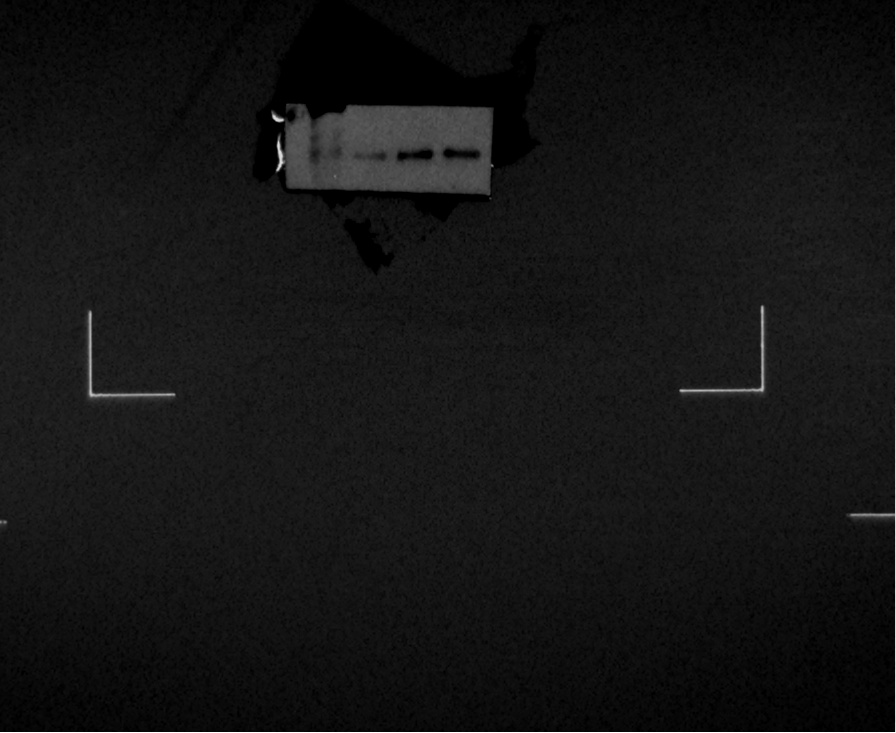

Supplement: Supplementary file 5 [file DataSheet1.ZIP › Supplementary file 2—— Original Image for Figure3(H-M):Western Blot/4.Figure3H—PPARγ/Figure3H—PPARγ-1.jpg]

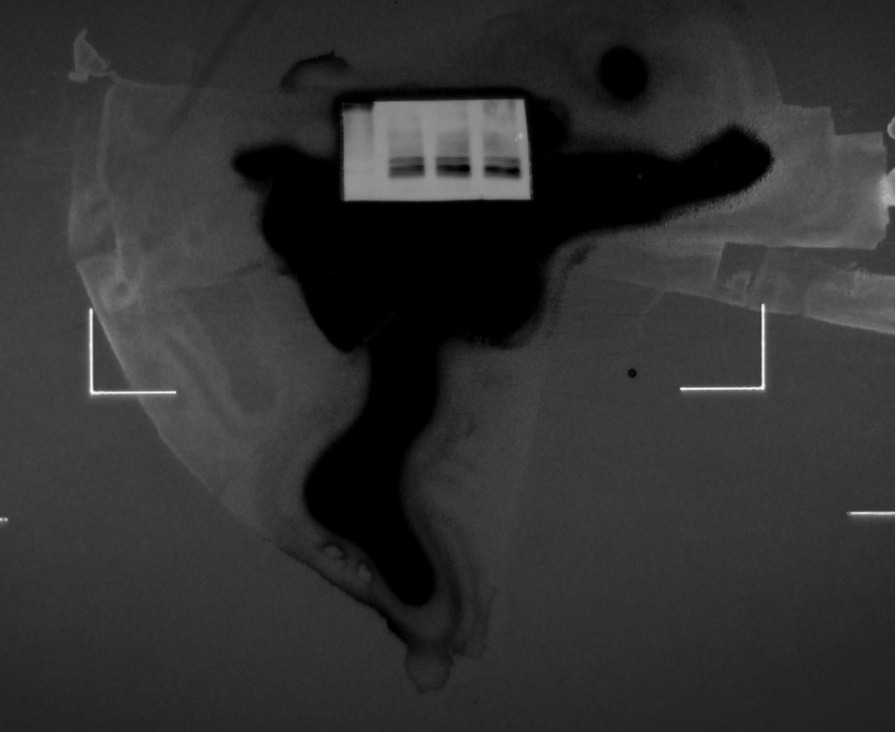

Supplement: Supplementary file 5 [file DataSheet1.ZIP › Supplementary file 2—— Original Image for Figure3(H-M):Western Blot/4.Figure3H—PPARγ/Figure3H—PPARγ-2.jpg]

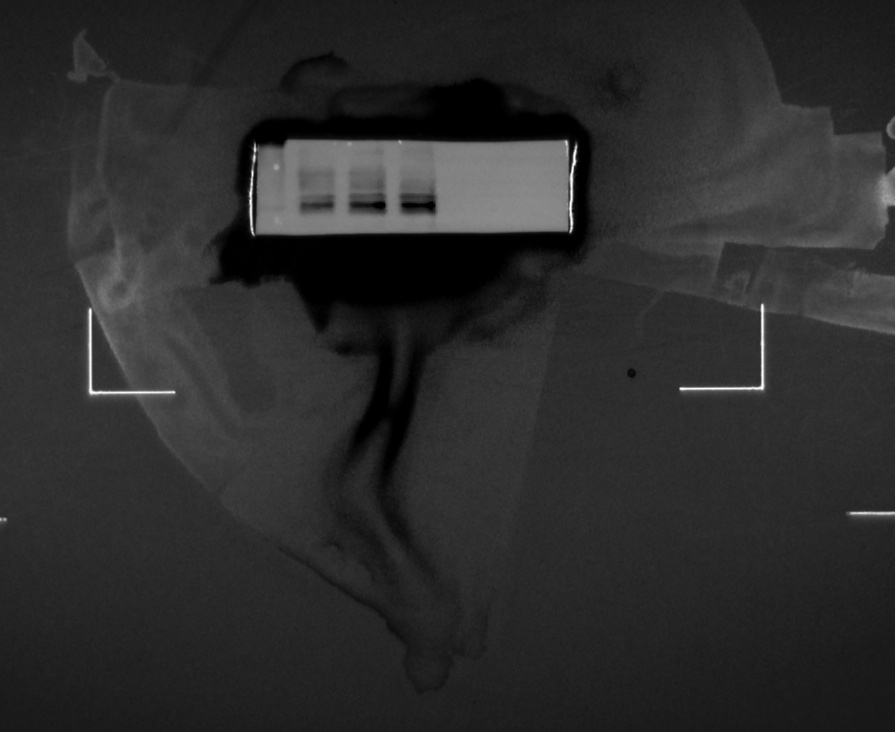

Supplement: Supplementary file 5 [file DataSheet1.ZIP › Supplementary file 2—— Original Image for Figure3(H-M):Western Blot/4.Figure3H—PPARγ/Figure3H—PPARγ-3.jpg]

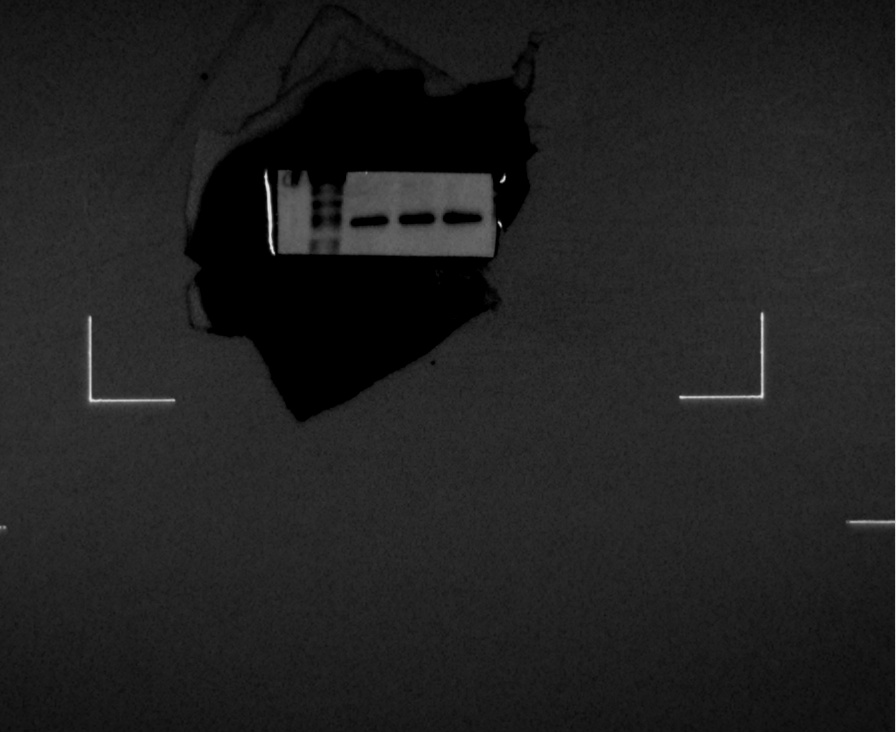

Supplement: Supplementary file 5 [file DataSheet1.ZIP › Supplementary file 2—— Original Image for Figure3(H-M):Western Blot/4.Figure3H—PPARγ/Figure3H—β-Tubulin-1.jpg]

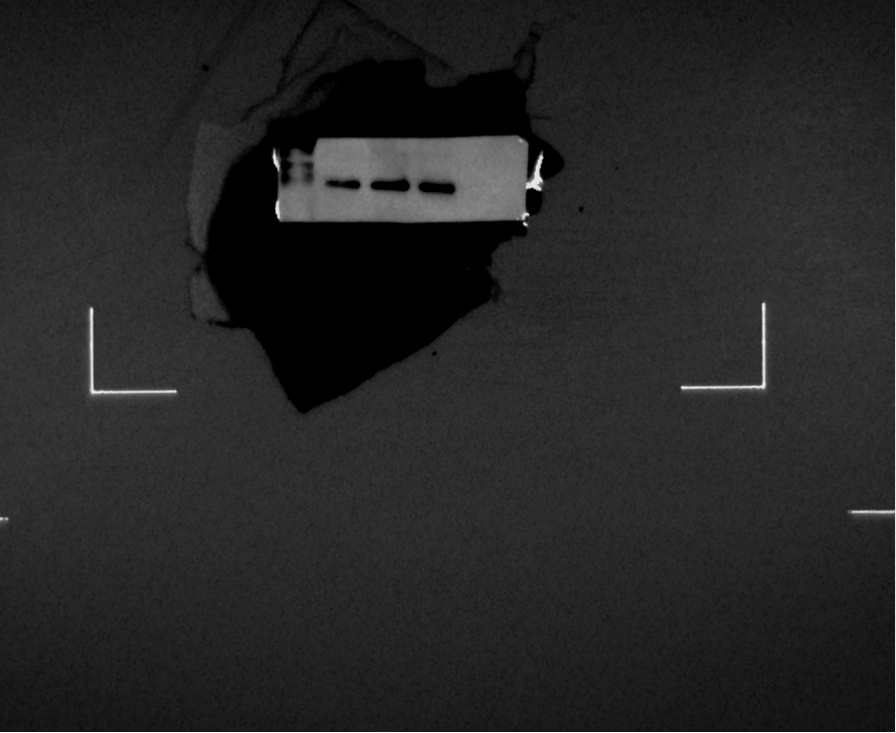

Supplement: Supplementary file 5 [file DataSheet1.ZIP › Supplementary file 2—— Original Image for Figure3(H-M):Western Blot/4.Figure3H—PPARγ/Figure3H—β-Tubulin-3.jpg]

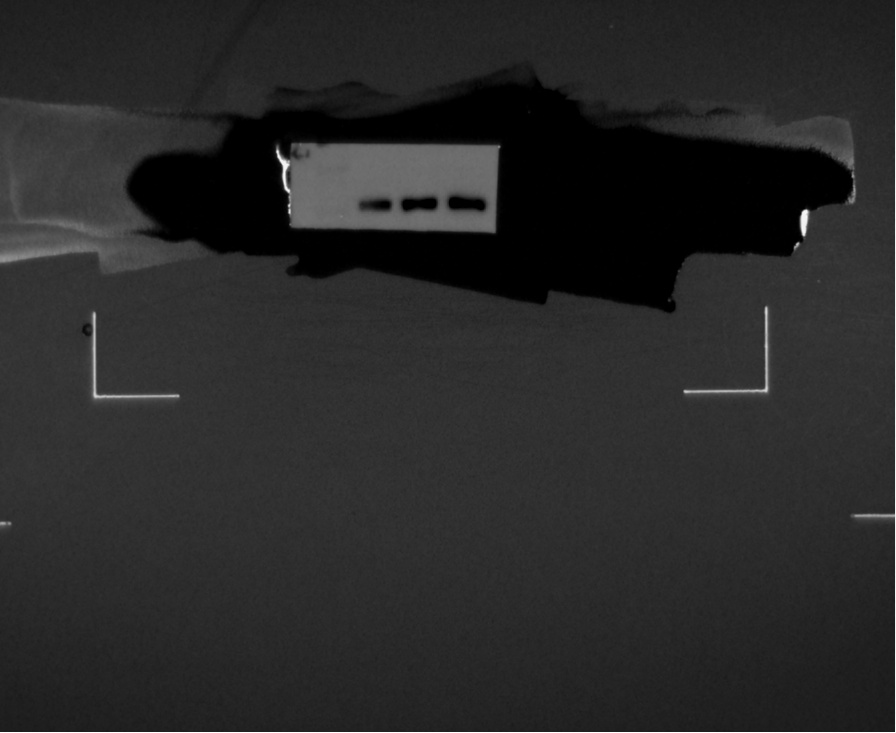

Supplement: Supplementary file 5 [file DataSheet1.ZIP › Supplementary file 2—— Original Image for Figure3(H-M):Western Blot/5.Figure3H—CTBP1/Figure3H—CTBP1-1.jpg]

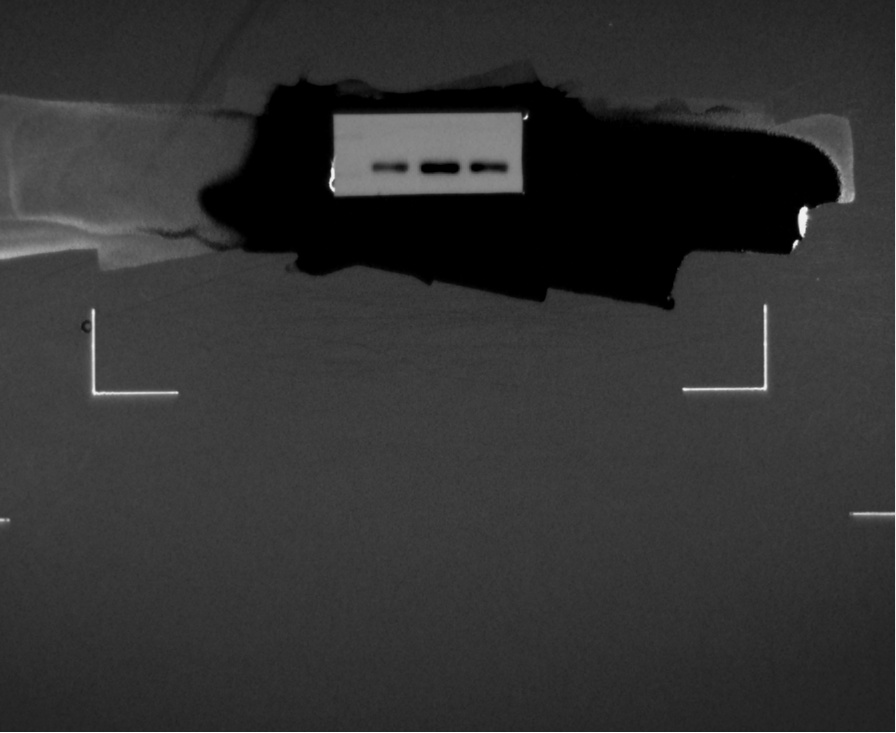

Supplement: Supplementary file 5 [file DataSheet1.ZIP › Supplementary file 2—— Original Image for Figure3(H-M):Western Blot/5.Figure3H—CTBP1/Figure3H—CTBP1-2.jpg]

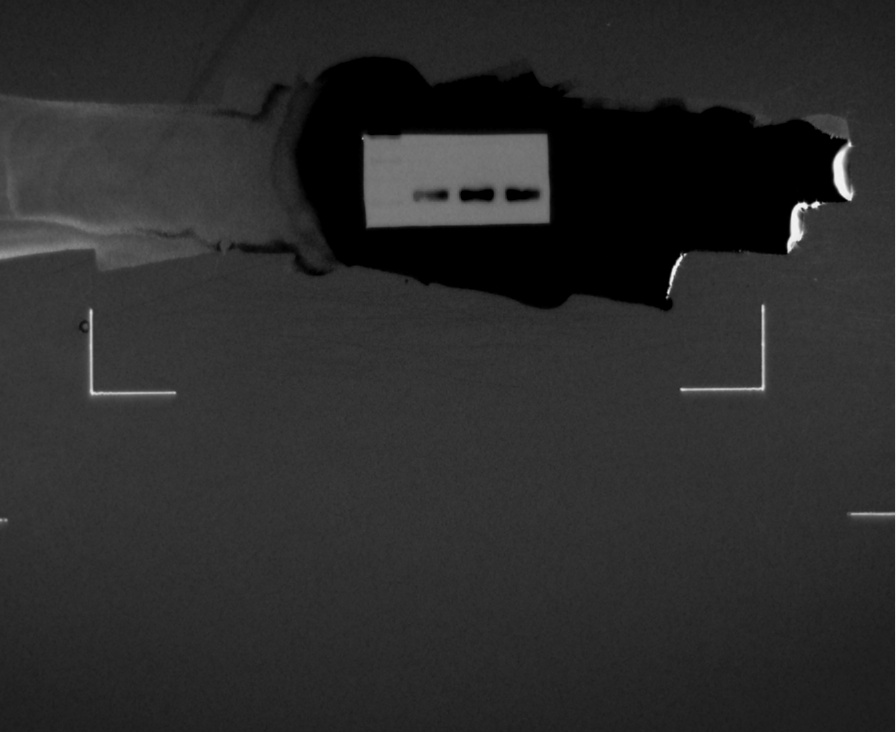

Supplement: Supplementary file 5 [file DataSheet1.ZIP › Supplementary file 2—— Original Image for Figure3(H-M):Western Blot/5.Figure3H—CTBP1/Figure3H—CTBP1-3.jpg]

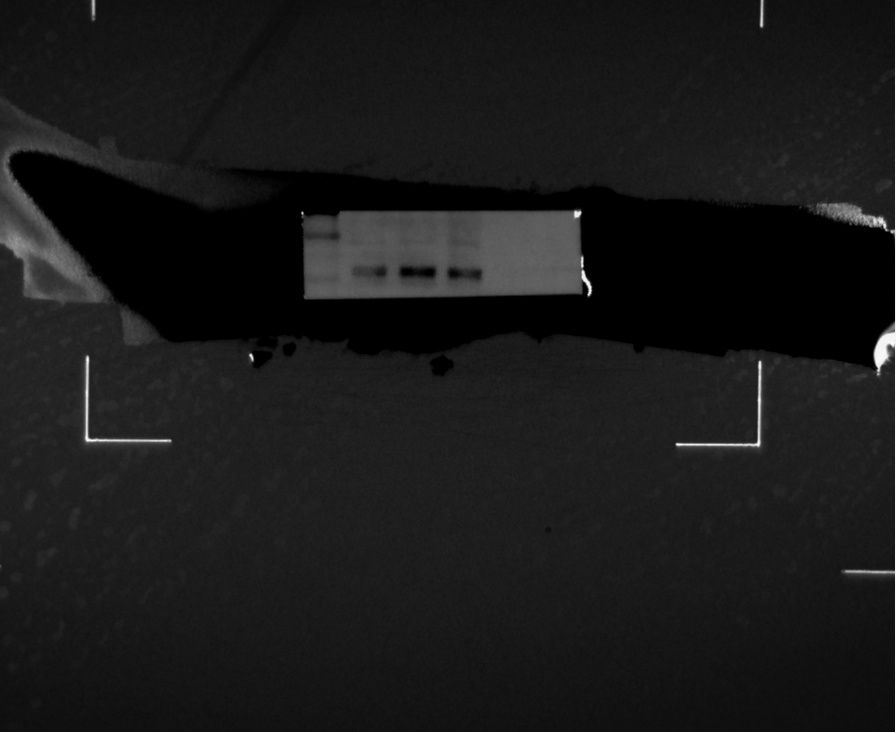

Supplement: Supplementary file 5 [file DataSheet1.ZIP › Supplementary file 2—— Original Image for Figure3(H-M):Western Blot/6.Figure3H—CTBP2/Figure3H—CTBP2-1.jpg]

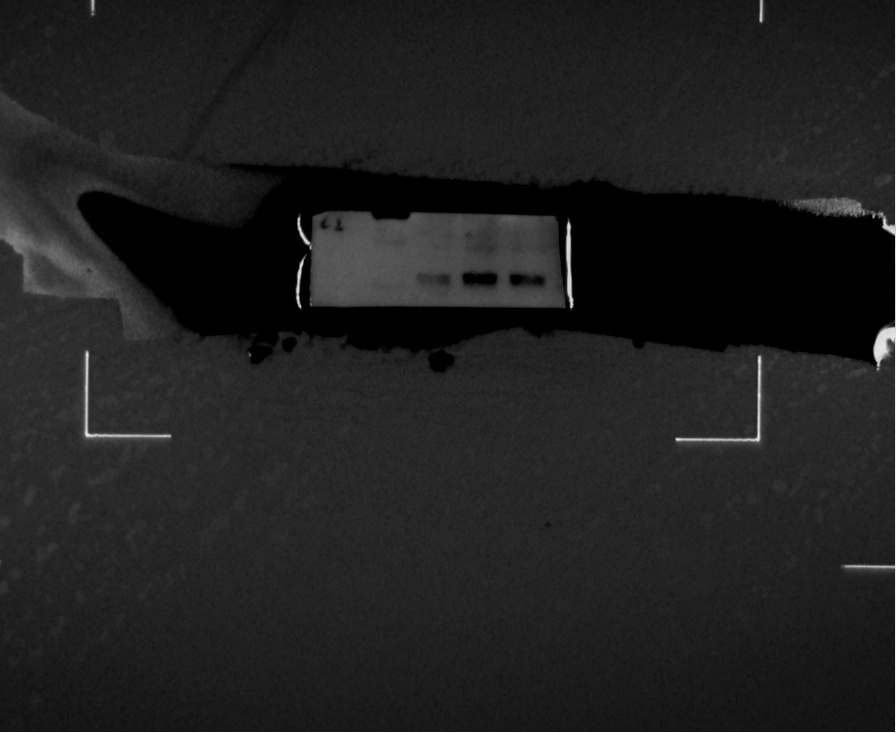

Supplement: Supplementary file 5 [file DataSheet1.ZIP › Supplementary file 2—— Original Image for Figure3(H-M):Western Blot/6.Figure3H—CTBP2/Figure3H—CTBP2-2.jpg]

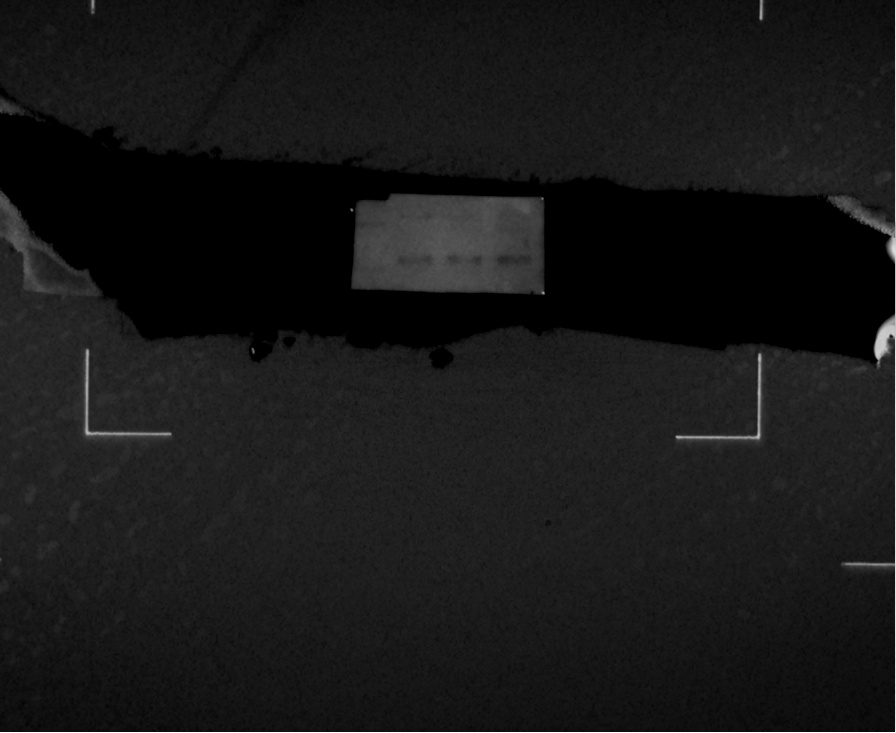

Supplement: Supplementary file 5 [file DataSheet1.ZIP › Supplementary file 2—— Original Image for Figure3(H-M):Western Blot/6.Figure3H—CTBP2/Figure3H—CTBP2-3.jpg]

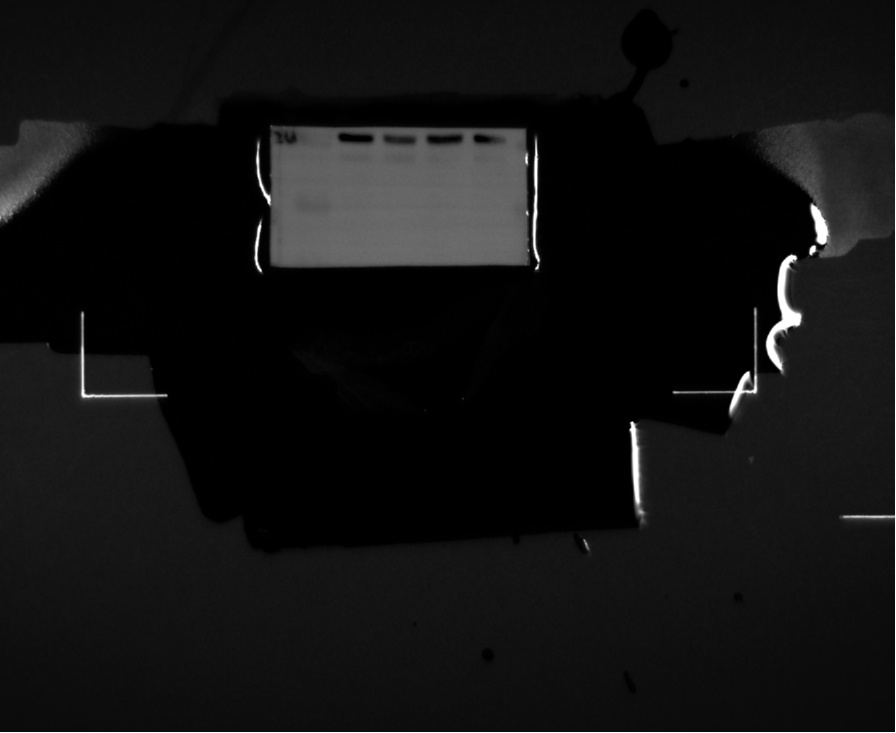

Supplement: Supplementary file 8 [file DataSheet2.ZIP › Supplementary file 3—— Original Image for Figure4(H-M):Western Blot/1.Figure4H—UCP1/Figure4H—UCP1-1.jpg]

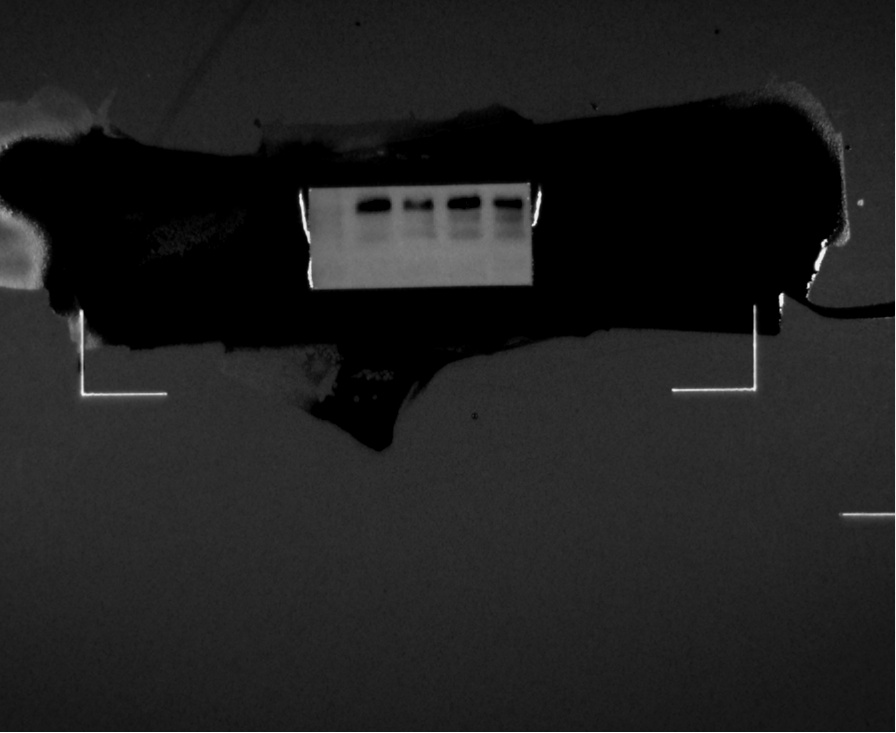

Supplement: Supplementary file 8 [file DataSheet2.ZIP › Supplementary file 3—— Original Image for Figure4(H-M):Western Blot/1.Figure4H—UCP1/Figure4H—UCP1-2.jpg]

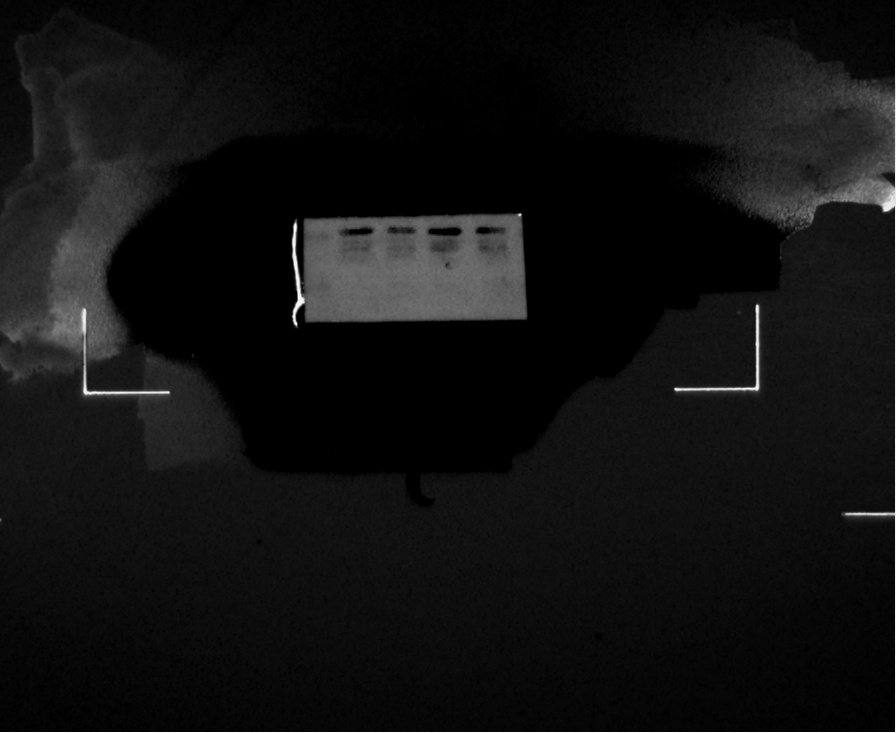

Supplement: Supplementary file 8 [file DataSheet2.ZIP › Supplementary file 3—— Original Image for Figure4(H-M):Western Blot/1.Figure4H—UCP1/Figure4H—UCP1-3.jpg]

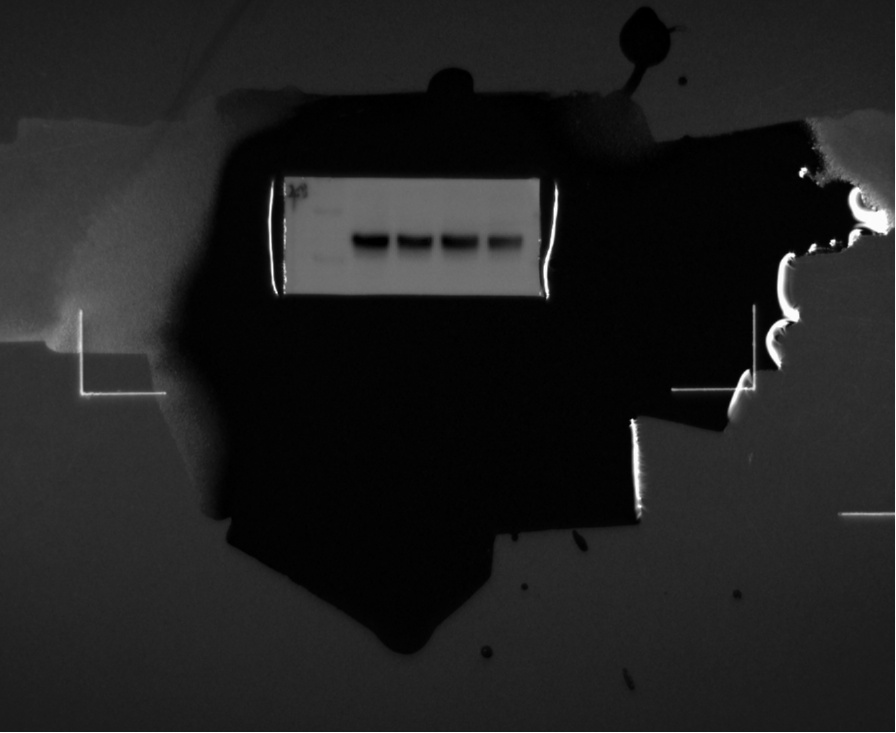

Supplement: Supplementary file 8 [file DataSheet2.ZIP › Supplementary file 3—— Original Image for Figure4(H-M):Western Blot/1.Figure4H—UCP1/Figure4H—β-Tubulin-1.jpg]

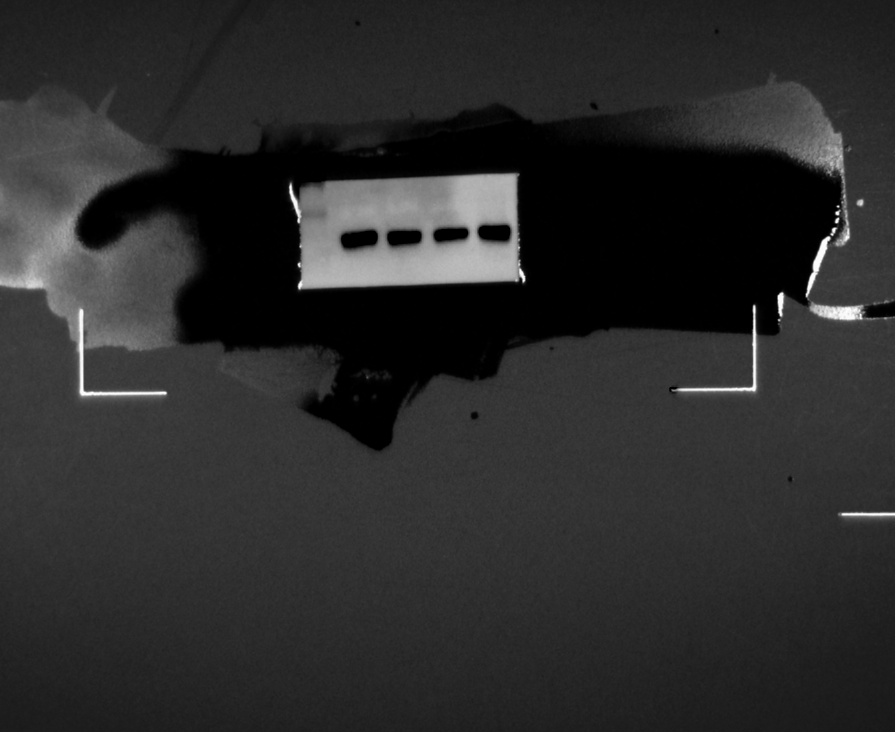

Supplement: Supplementary file 8 [file DataSheet2.ZIP › Supplementary file 3—— Original Image for Figure4(H-M):Western Blot/1.Figure4H—UCP1/Figure4H—β-Tubulin-2.jpg]

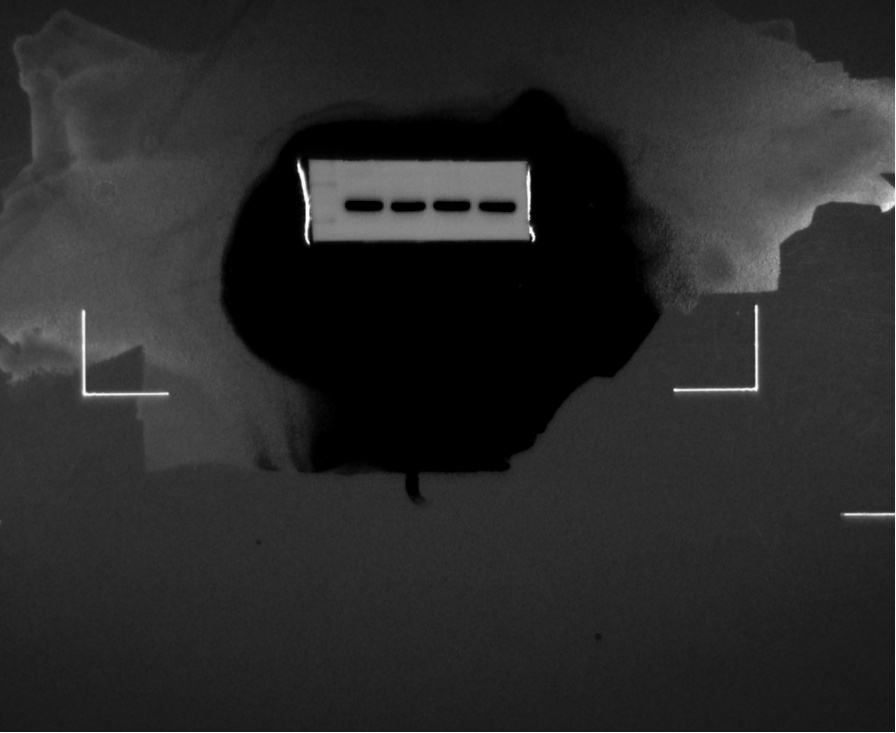

Supplement: Supplementary file 8 [file DataSheet2.ZIP › Supplementary file 3—— Original Image for Figure4(H-M):Western Blot/1.Figure4H—UCP1/Figure4H—β-Tubulin-3.jpg]

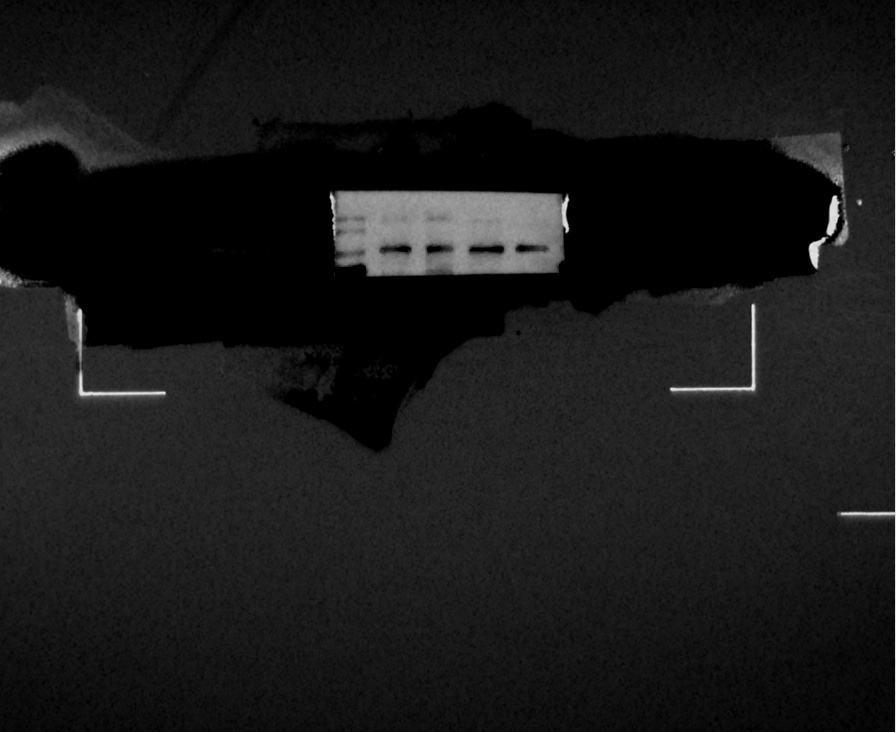

Supplement: Supplementary file 8 [file DataSheet2.ZIP › Supplementary file 3—— Original Image for Figure4(H-M):Western Blot/2.Figure4H—PRDM16/Figure4H—PRDM16-1.jpg]

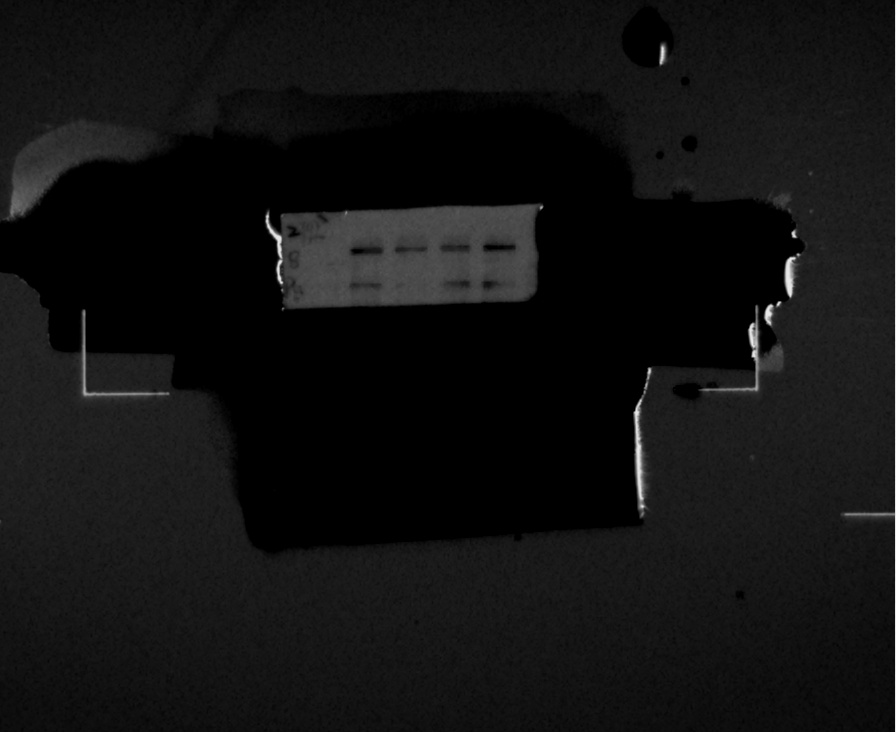

Supplement: Supplementary file 8 [file DataSheet2.ZIP › Supplementary file 3—— Original Image for Figure4(H-M):Western Blot/2.Figure4H—PRDM16/Figure4H—PRDM16-2.jpg]

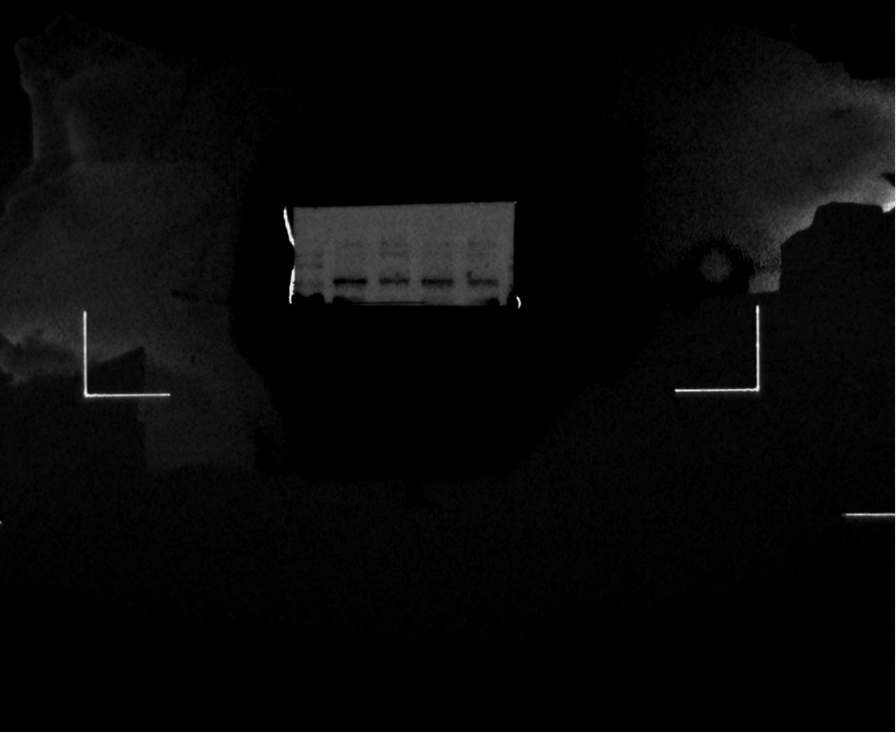

Supplement: Supplementary file 8 [file DataSheet2.ZIP › Supplementary file 3—— Original Image for Figure4(H-M):Western Blot/2.Figure4H—PRDM16/Figure4H—PRDM16-3.jpg]

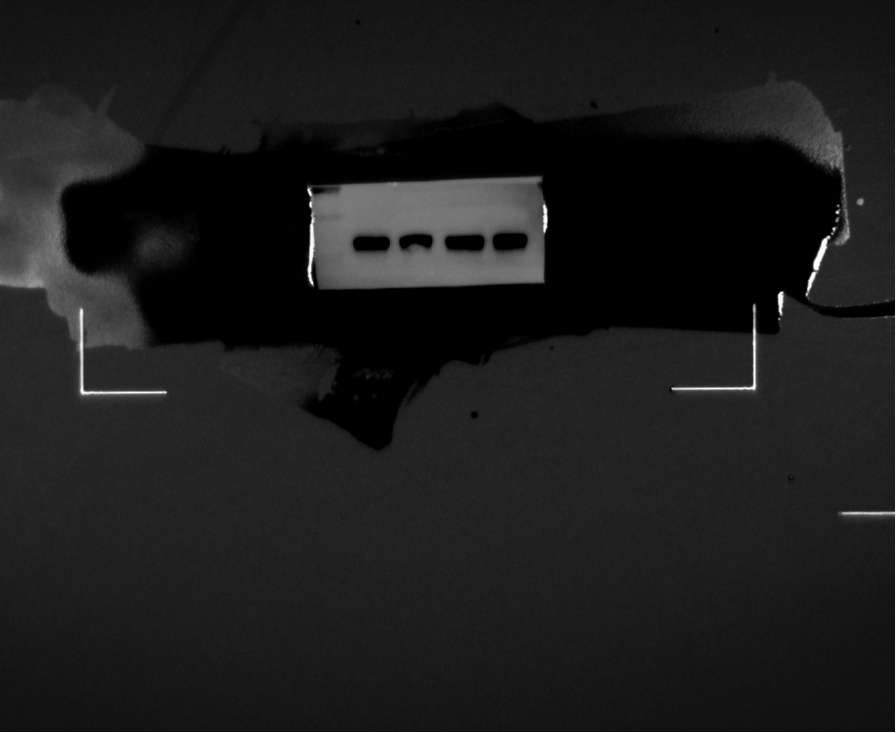

Supplement: Supplementary file 8 [file DataSheet2.ZIP › Supplementary file 3—— Original Image for Figure4(H-M):Western Blot/2.Figure4H—PRDM16/Figure4H—β-Tubulin-1.jpg]

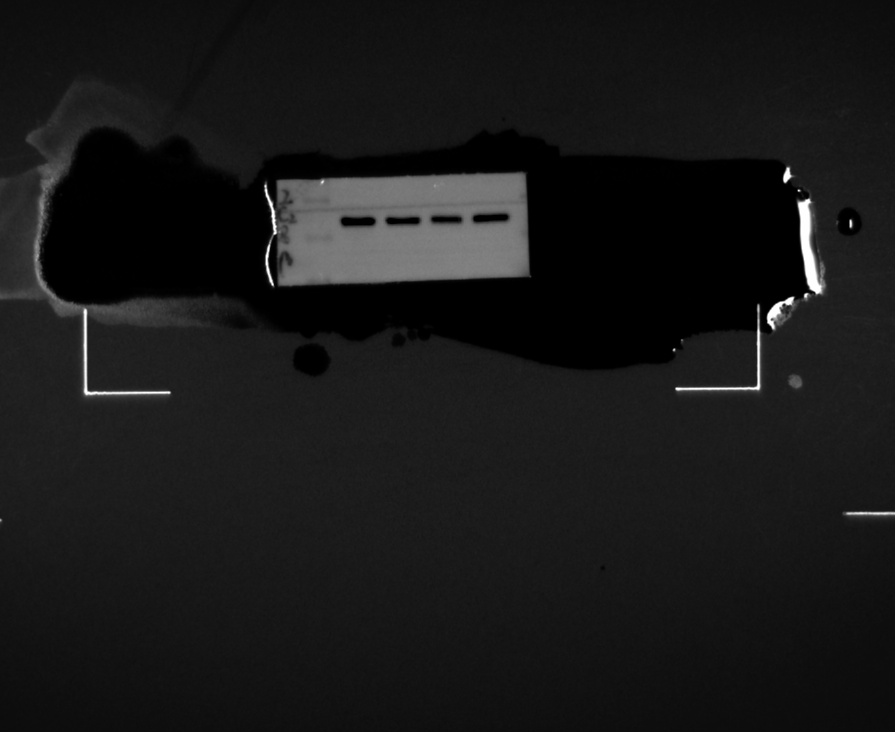

Supplement: Supplementary file 8 [file DataSheet2.ZIP › Supplementary file 3—— Original Image for Figure4(H-M):Western Blot/2.Figure4H—PRDM16/Figure4H—β-Tubulin-2.jpg]

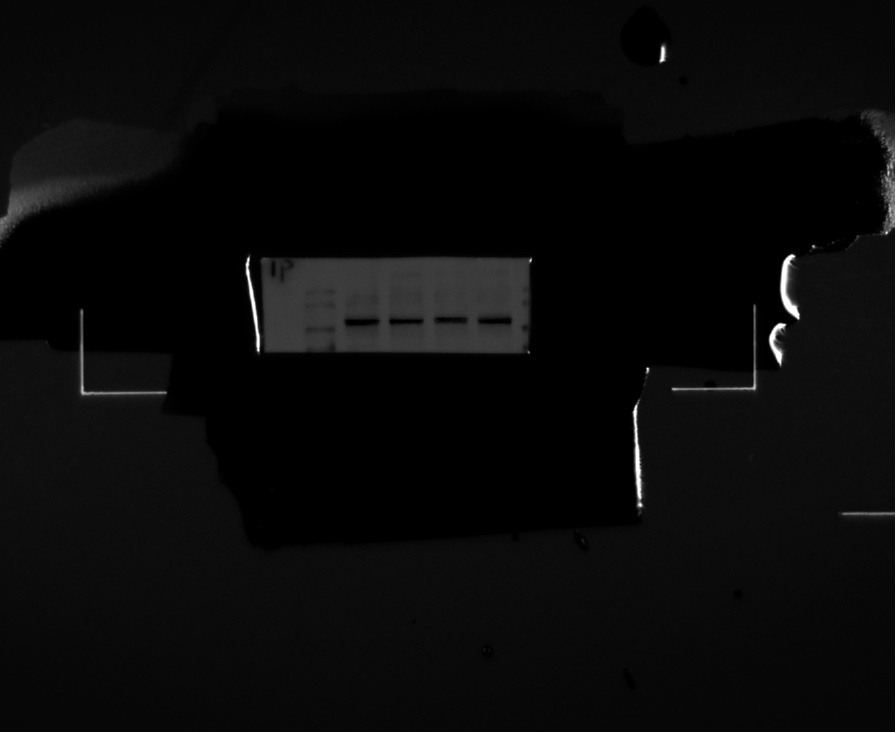

Supplement: Supplementary file 8 [file DataSheet2.ZIP › Supplementary file 3—— Original Image for Figure4(H-M):Western Blot/3.Figure4H—PGC-1α/Figure4H—PGC-1α-1.jpg]

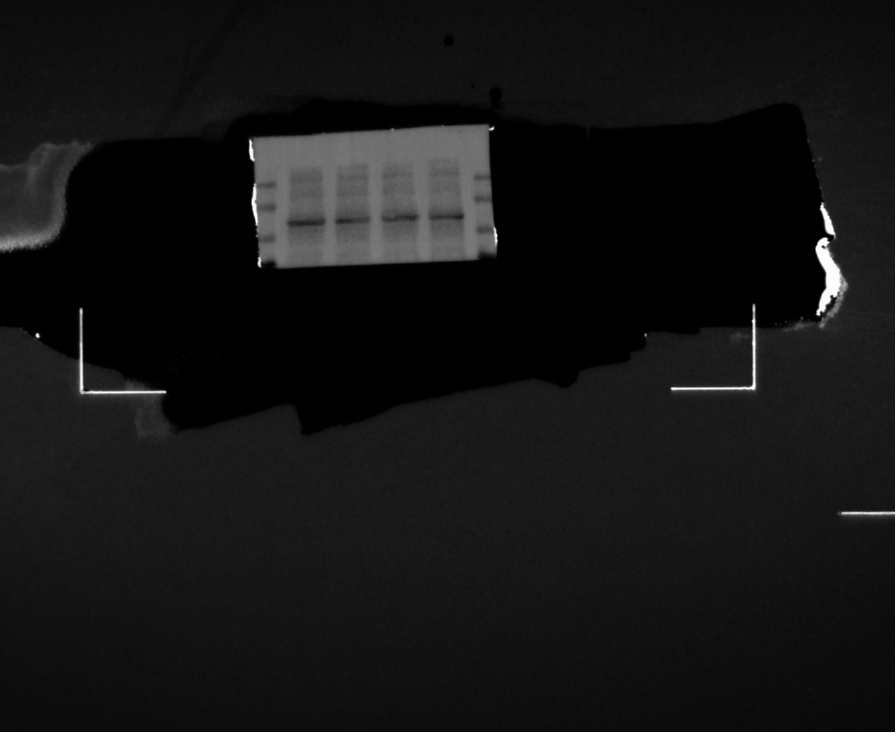

Supplement: Supplementary file 8 [file DataSheet2.ZIP › Supplementary file 3—— Original Image for Figure4(H-M):Western Blot/3.Figure4H—PGC-1α/Figure4H—PGC-1α-2.jpg]

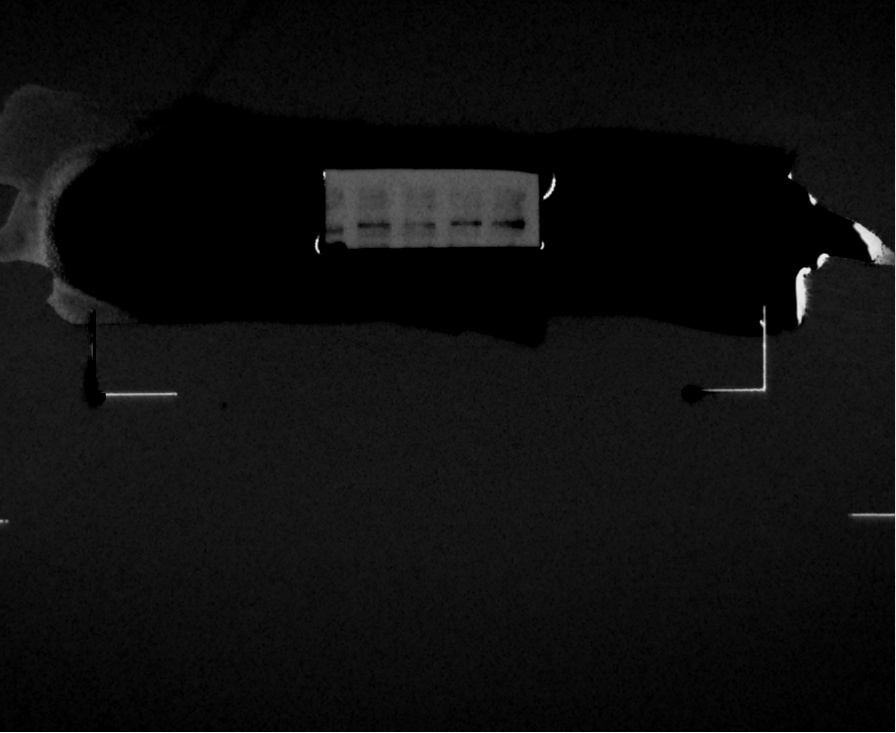

Supplement: Supplementary file 8 [file DataSheet2.ZIP › Supplementary file 3—— Original Image for Figure4(H-M):Western Blot/3.Figure4H—PGC-1α/Figure4H—PGC-1α-3.jpg]

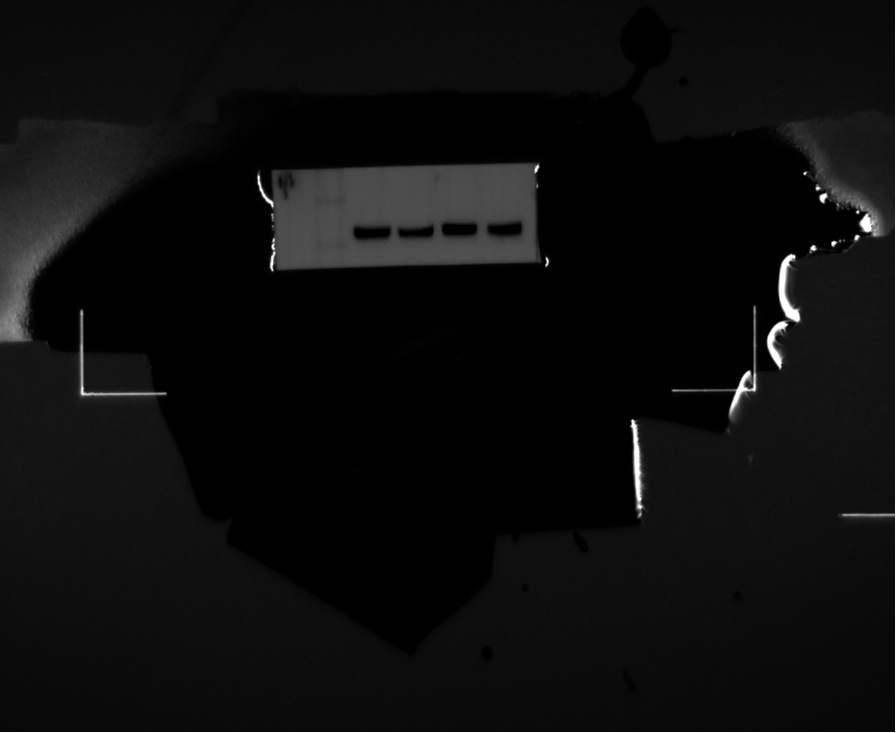

Supplement: Supplementary file 8 [file DataSheet2.ZIP › Supplementary file 3—— Original Image for Figure4(H-M):Western Blot/3.Figure4H—PGC-1α/Figure4H—β-Tubulin-1.jpg]

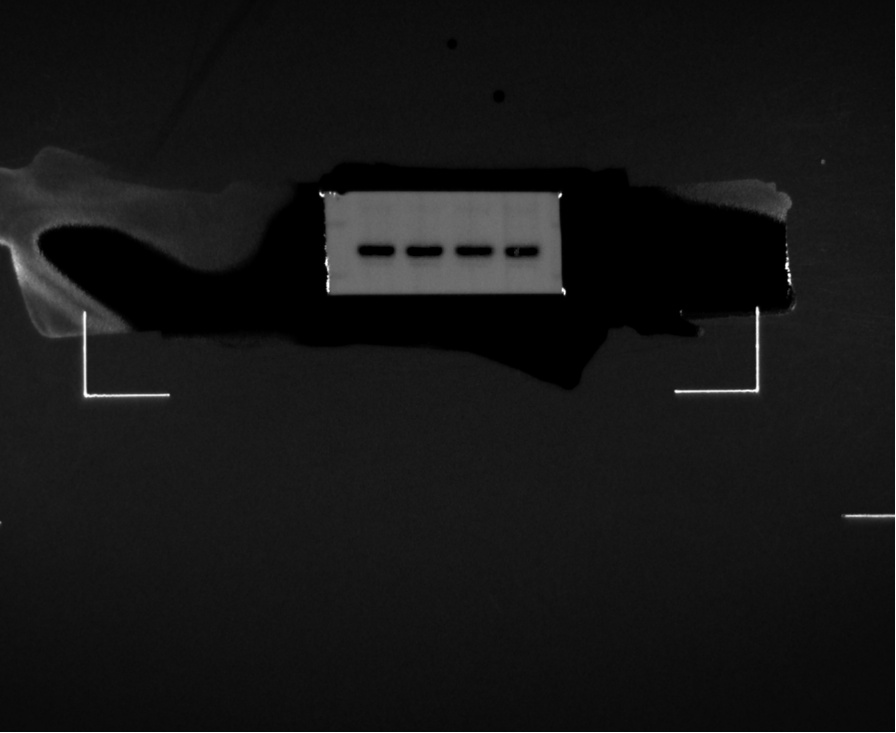

Supplement: Supplementary file 8 [file DataSheet2.ZIP › Supplementary file 3—— Original Image for Figure4(H-M):Western Blot/3.Figure4H—PGC-1α/Figure4H—β-Tubulin-2.jpg]

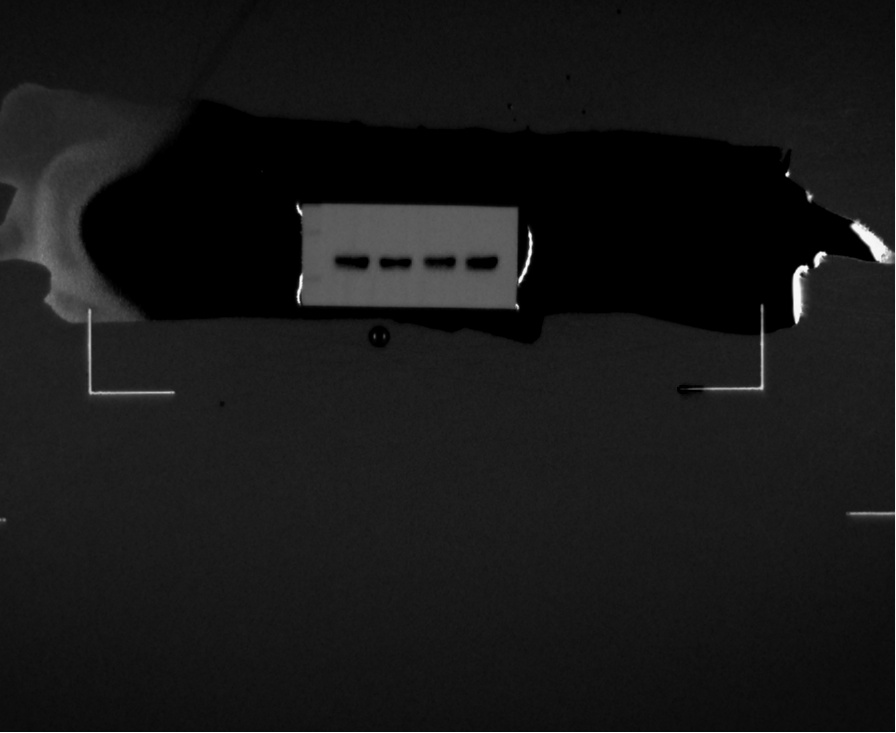

Supplement: Supplementary file 8 [file DataSheet2.ZIP › Supplementary file 3—— Original Image for Figure4(H-M):Western Blot/3.Figure4H—PGC-1α/Figure4H—β-Tubulin-3.jpg]

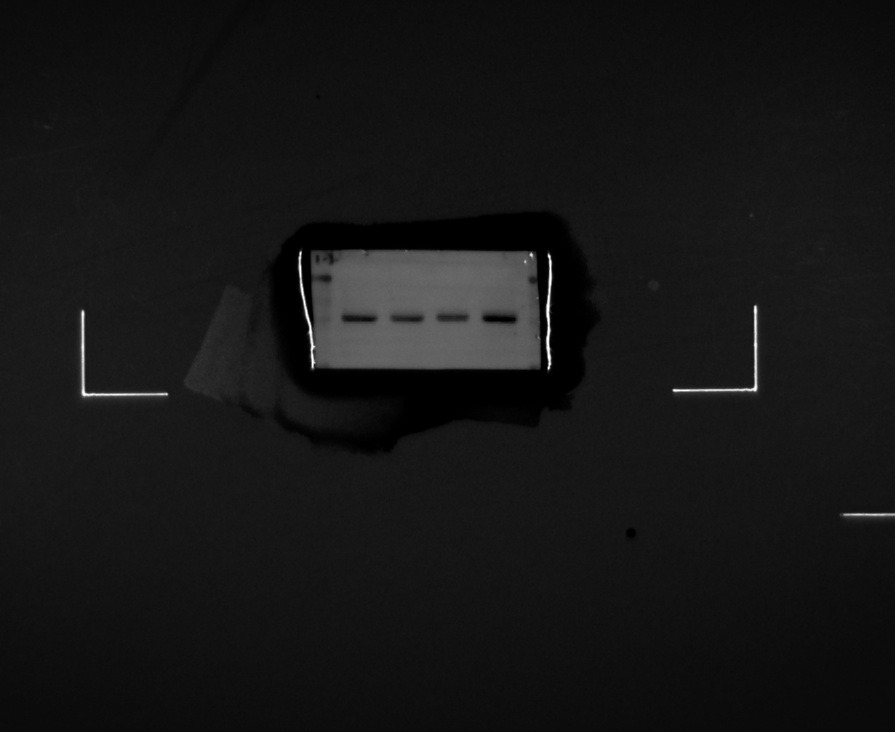

Supplement: Supplementary file 8 [file DataSheet2.ZIP › Supplementary file 3—— Original Image for Figure4(H-M):Western Blot/4.Figure4H—PPARγ/Figure4H—PPARγ-1.jpg]

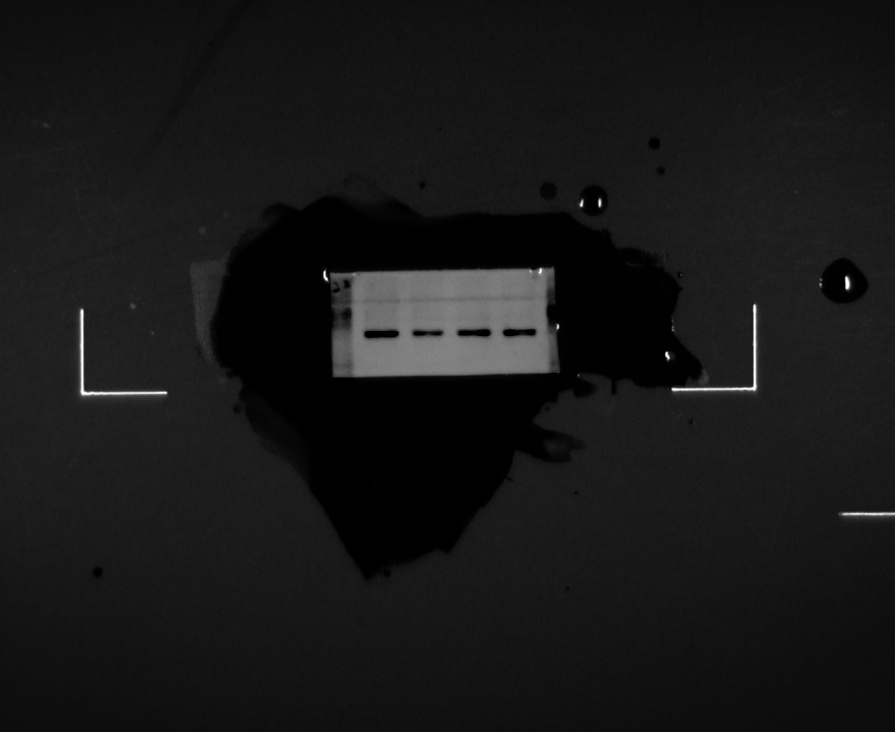

Supplement: Supplementary file 8 [file DataSheet2.ZIP › Supplementary file 3—— Original Image for Figure4(H-M):Western Blot/4.Figure4H—PPARγ/Figure4H—PPARγ-2.jpg]

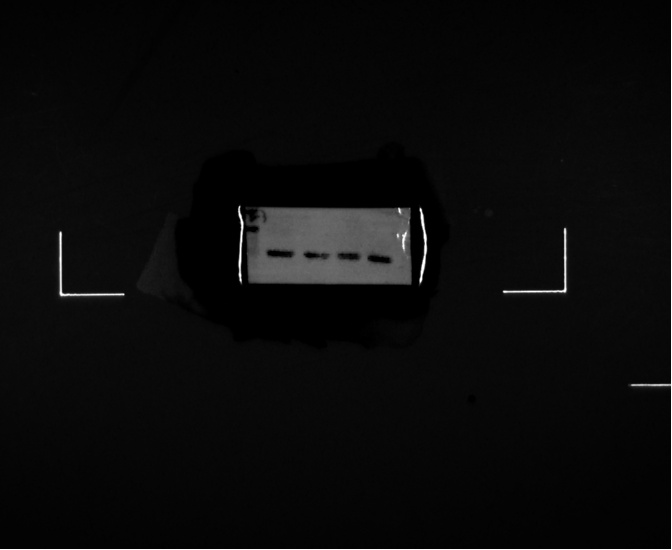

Supplement: Supplementary file 8 [file DataSheet2.ZIP › Supplementary file 3—— Original Image for Figure4(H-M):Western Blot/4.Figure4H—PPARγ/Figure4H—PPARγ-3.jpg]

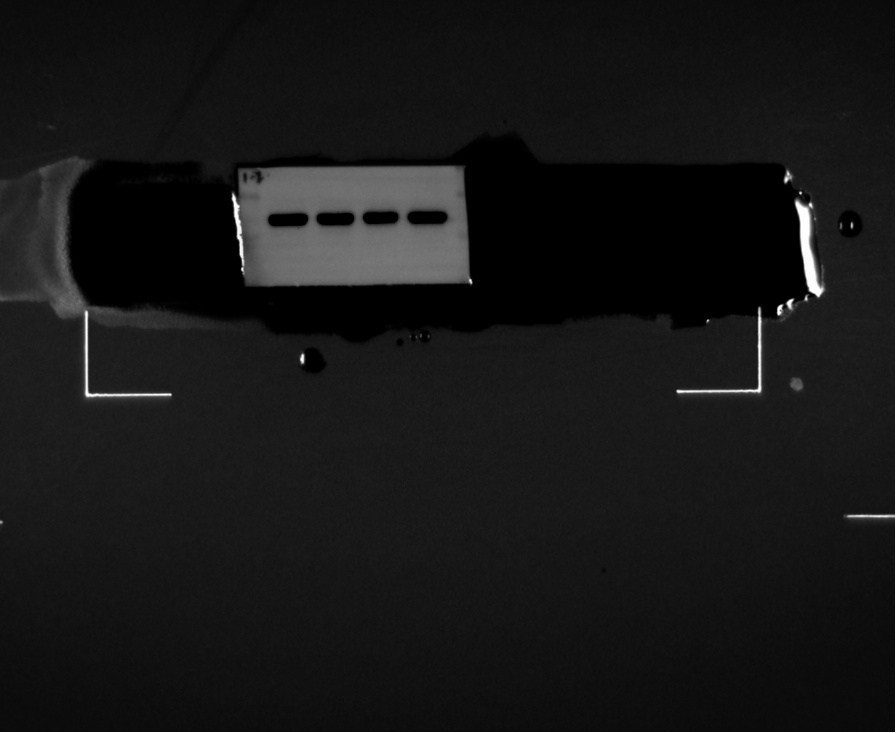

Supplement: Supplementary file 8 [file DataSheet2.ZIP › Supplementary file 3—— Original Image for Figure4(H-M):Western Blot/4.Figure4H—PPARγ/Figure4H—β-Tubulin-1.jpg]

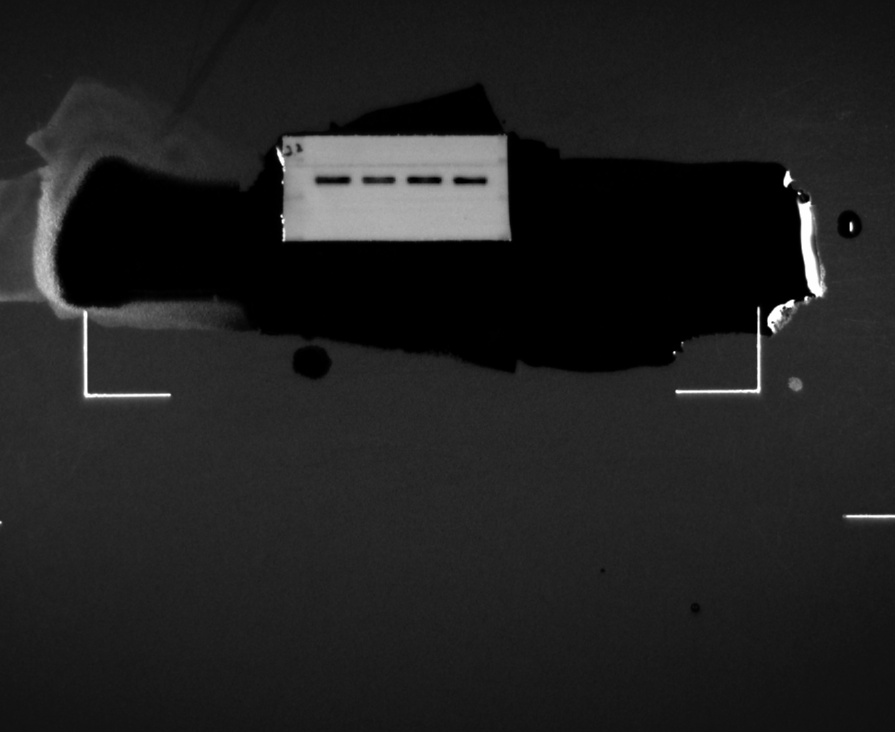

Supplement: Supplementary file 8 [file DataSheet2.ZIP › Supplementary file 3—— Original Image for Figure4(H-M):Western Blot/4.Figure4H—PPARγ/Figure4H—β-Tubulin-2.jpg]

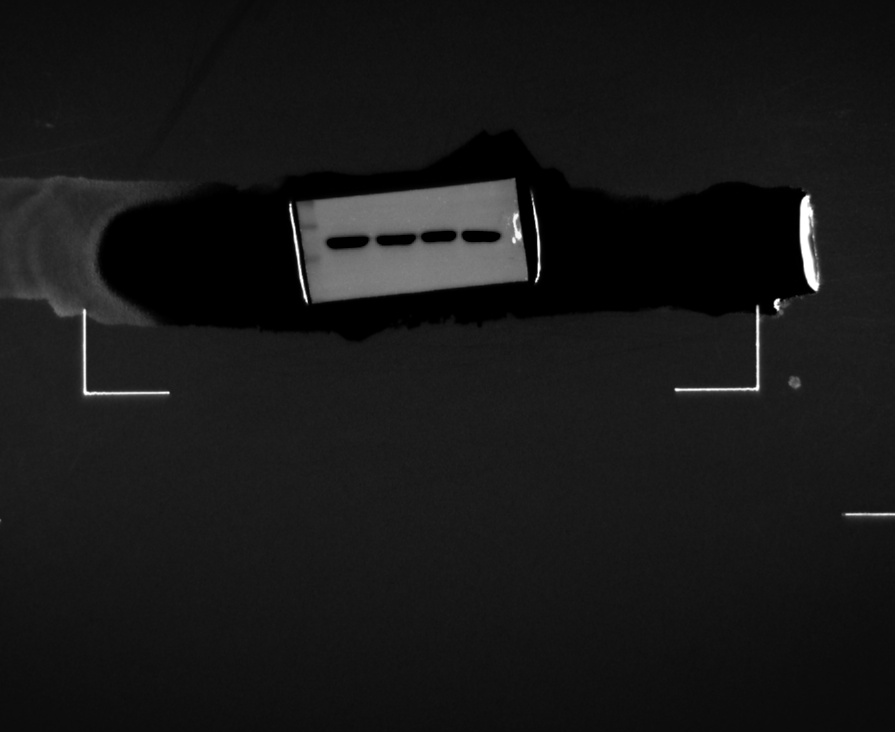

Supplement: Supplementary file 8 [file DataSheet2.ZIP › Supplementary file 3—— Original Image for Figure4(H-M):Western Blot/4.Figure4H—PPARγ/Figure4H—β-Tubulin-3.jpg]

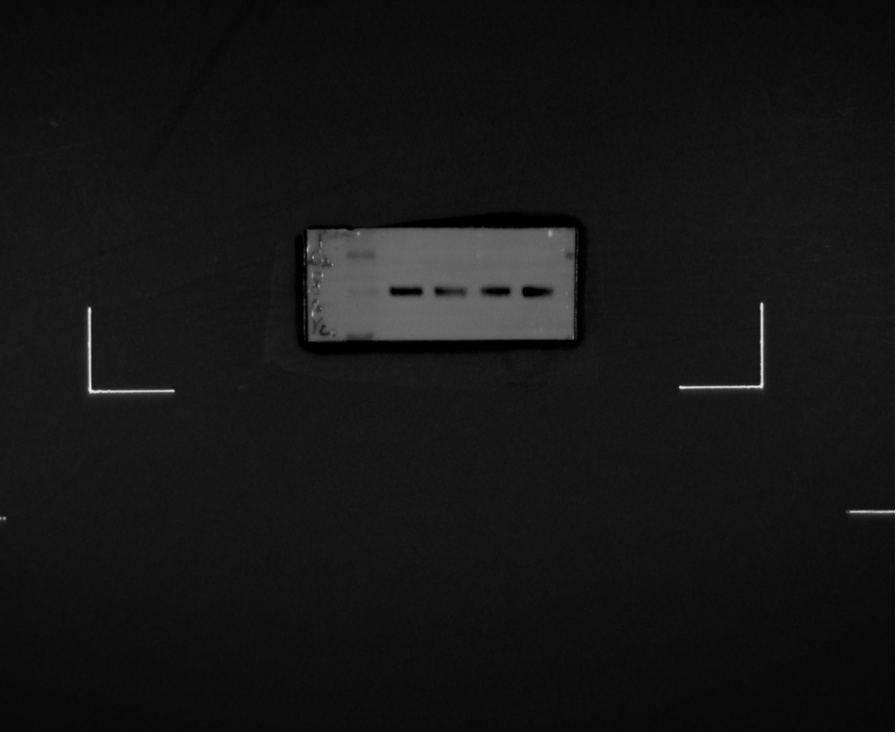

Supplement: Supplementary file 8 [file DataSheet2.ZIP › Supplementary file 3—— Original Image for Figure4(H-M):Western Blot/5.Figure4H—CTBP1/Figure4H—CTBP1-1.jpg]

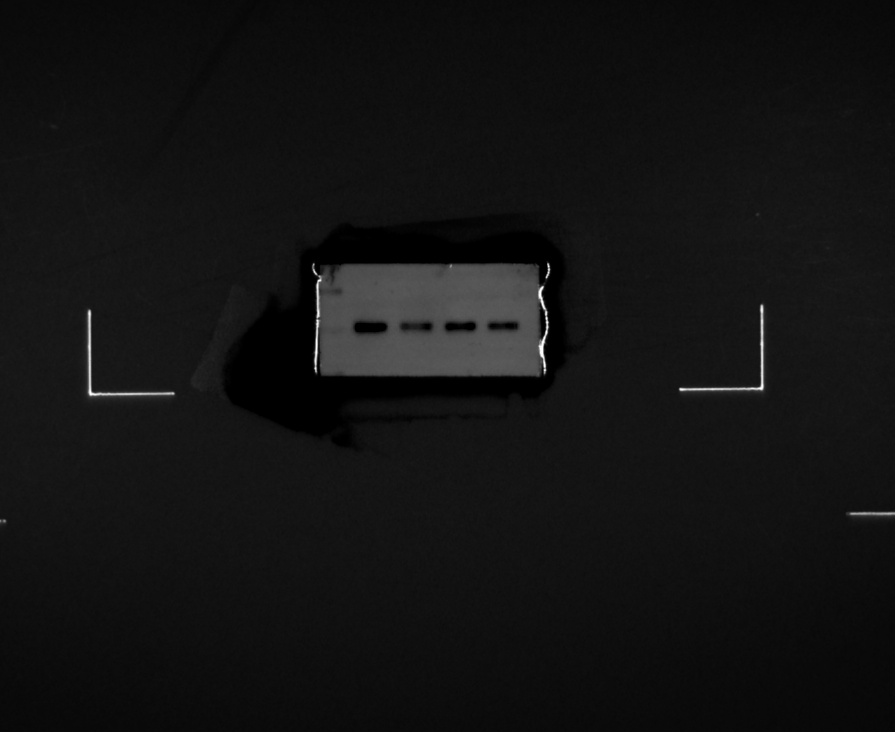

Supplement: Supplementary file 8 [file DataSheet2.ZIP › Supplementary file 3—— Original Image for Figure4(H-M):Western Blot/5.Figure4H—CTBP1/Figure4H—CTBP1-2.jpg]

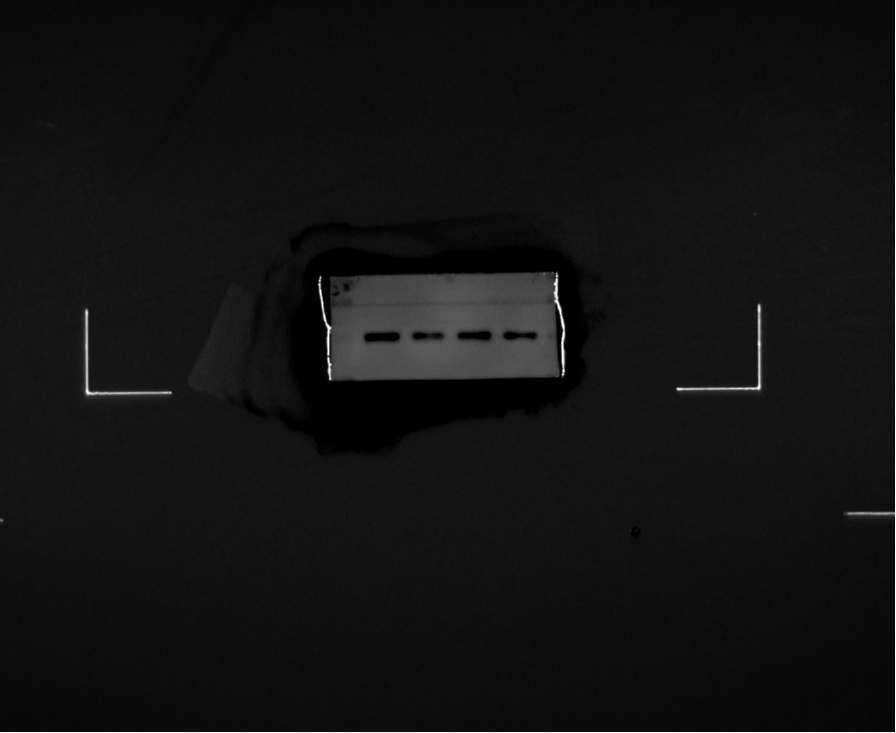

Supplement: Supplementary file 8 [file DataSheet2.ZIP › Supplementary file 3—— Original Image for Figure4(H-M):Western Blot/5.Figure4H—CTBP1/Figure4H—CTBP1-3.jpg]

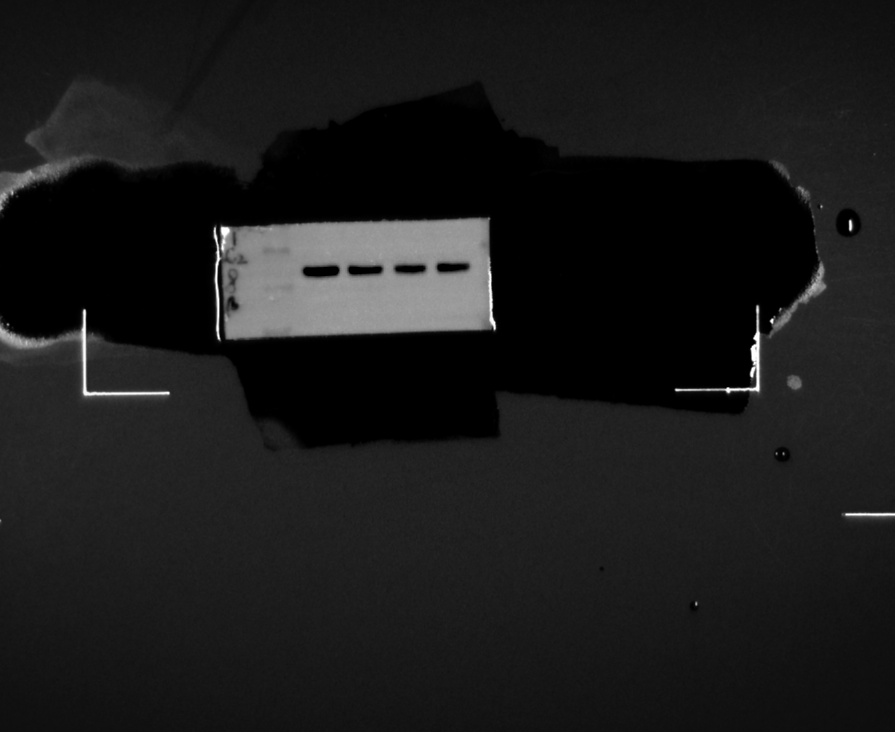

Supplement: Supplementary file 8 [file DataSheet2.ZIP › Supplementary file 3—— Original Image for Figure4(H-M):Western Blot/5.Figure4H—CTBP1/Figure4H—β-Tubulin-1.jpg]

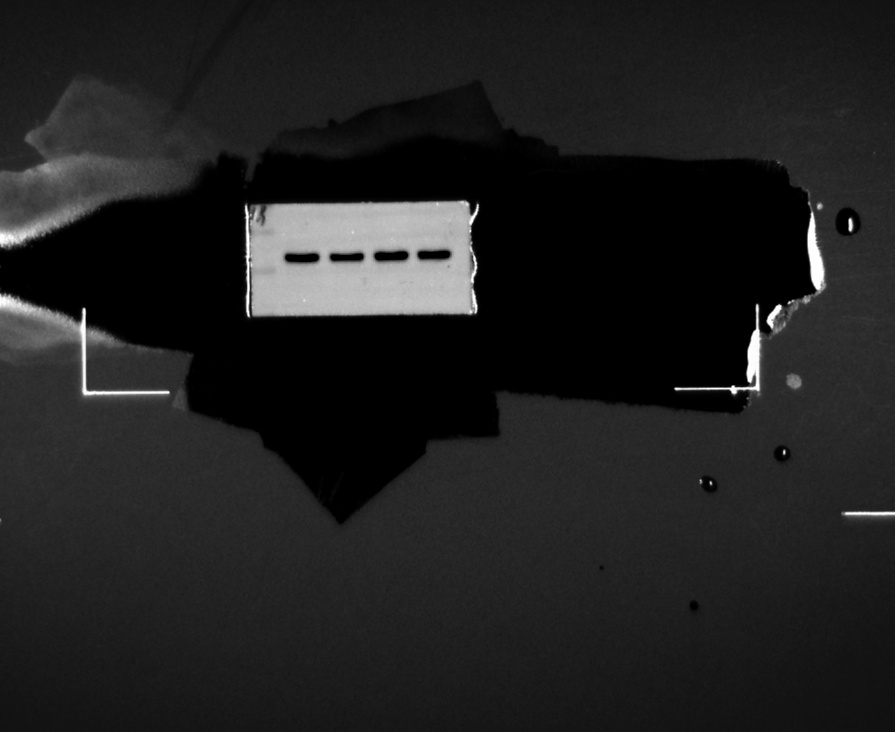

Supplement: Supplementary file 8 [file DataSheet2.ZIP › Supplementary file 3—— Original Image for Figure4(H-M):Western Blot/5.Figure4H—CTBP1/Figure4H—β-Tubulin-2.jpg]

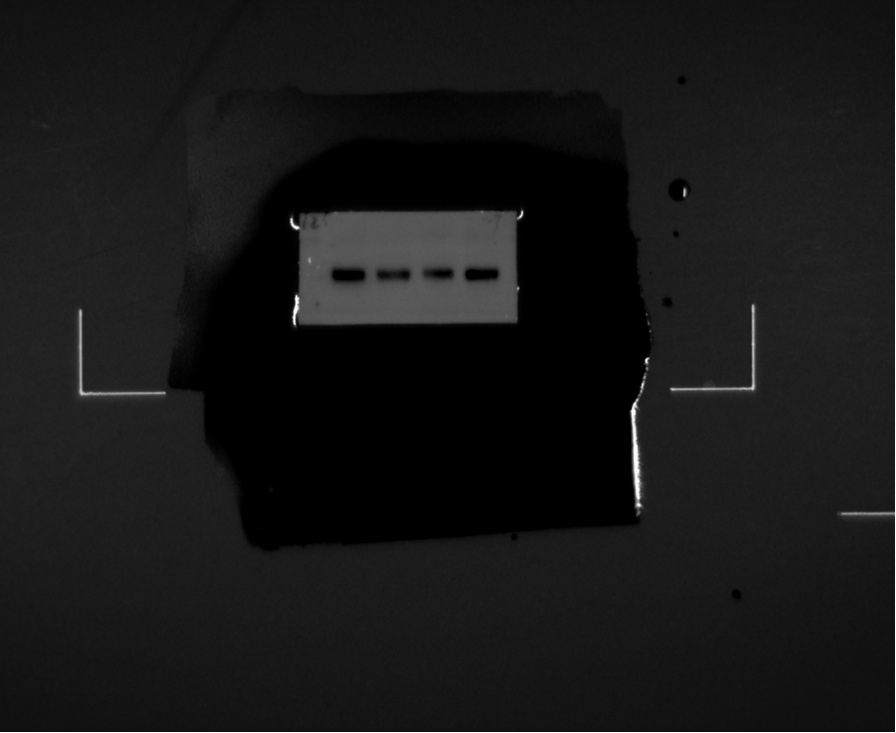

Supplement: Supplementary file 8 [file DataSheet2.ZIP › Supplementary file 3—— Original Image for Figure4(H-M):Western Blot/6.Figure4H—CTBP2/Figure4H—CTBP2-1.jpg]

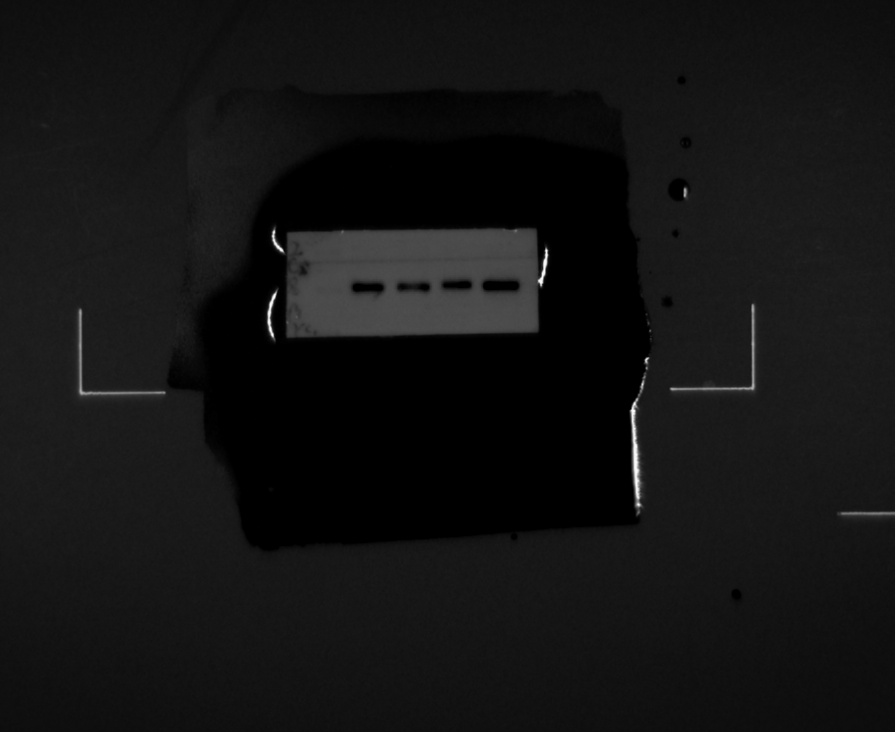

Supplement: Supplementary file 8 [file DataSheet2.ZIP › Supplementary file 3—— Original Image for Figure4(H-M):Western Blot/6.Figure4H—CTBP2/Figure4H—CTBP2-2.jpg]

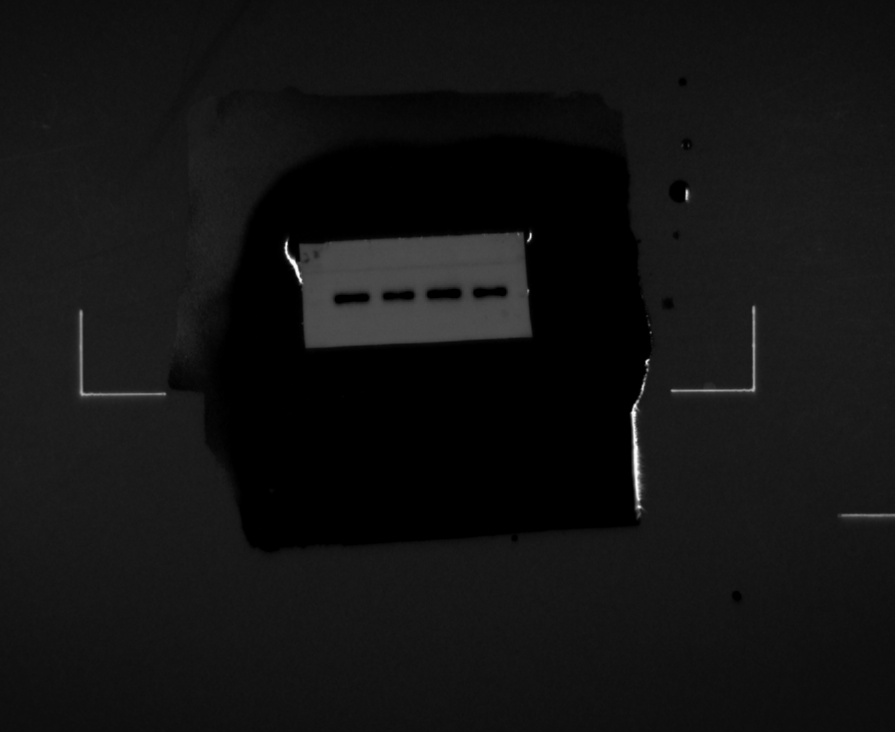

Supplement: Supplementary file 8 [file DataSheet2.ZIP › Supplementary file 3—— Original Image for Figure4(H-M):Western Blot/6.Figure4H—CTBP2/Figure4H—CTBP2-3.jpg]

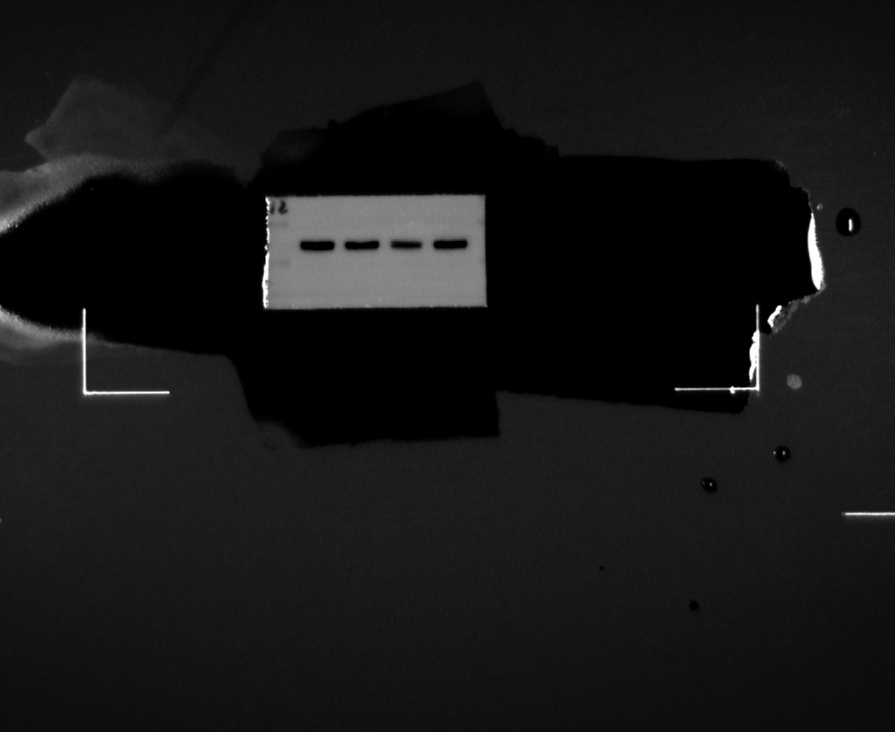

Supplement: Supplementary file 8 [file DataSheet2.ZIP › Supplementary file 3—— Original Image for Figure4(H-M):Western Blot/6.Figure4H—CTBP2/Figure4H—β-Tubulin-1.jpg]
