# Supplementary material for: Ling-gui-zhu-gan promotes adipocytes browning via targeting the miR-27b/PRDM16 pathway in 3T3-L1 cells
Source: Front Pharmacol. 2024 Aug 14;15:1386794. doi: 10.3389/fphar.2024.1386794 (PMC11349548; doi:10.3389/fphar.2024.1386794)
Supplement: Supplementary file 4 [file Presentation1.pdf]

Methods

Ultra-performance liquid chromatograph-hybrid quadrupole orbitrap high resolution mass spectrometer (UHPLC-Q-Orbitrap HRMS)

The chemical constituents of LGZG-containing serum were identified using UHPLC-Q-Orbitrap HRMS by Beijing HEXIN Technology Co., Ltd (Beijing, China). As described previously [1], the serum sample was thoroughly mixed with methanol and then centrifuged at 4 °C and 12,000 rpm for 10 min. 6 μL of supernatant was injected into an ACQUITY UPLC HSS T3 column (1.8 μm, 2.1 mm×100 mm) at 40 °C using a Vanquish Flex UHPLC chromatograph (Thermo Fisher Scientific, Inc., Waltham, MA, USA). Analytes were eluted using a gradient of mobile phase A (water with 0.1 % formic acid) and mobile phase B (methanol) at a flow rate of 0.3 mL/min. Subsequently, mass spectrometer (MS) was conducted using a Q Exactive™ quadrupole-orbitrap mass spectrometer (Thermo Fisher Scientific, Waltham, MA, USA) equipped with a HESI-II spray probe. Both positive (3.7 kV) and negative (3.5 kV) electrospray ionization modes were used. The MS data were acquired in full scan/dd-MS2 mode and processed using Progenesis QI 3.0 software (Waters Corp., MA, USA).

[1] You, L., Wang, T., Li, W., Zhang, J., Zheng, C., Zheng, Y., et al. (2024). Xiaozhi formula attenuates non-alcoholic fatty liver disease by regulating lipid metabolism via activation of AMPK and PPAR pathways. *J Ethnopharmacol* 329**,** 118165. doi: 10.1016/j.jep.2024.118165.

Results

The chemical characterization of LGZG-containing serum was performed by UHPLC-Q-Orbitrap HRMS analysis, with the main constituents presented in **Supplementary Figure S1** and **Supplementary Table S1**. A total of 49 constituents were identified from the LGZG-containing serum through data matching, consisting of 11 flavonoids, 9 isoflavonoids, 2 neoflavonoids, 17 triterpenoids, 2 bile acids, alcohols and derivatives, 1 chalcones and dihydrochalcones, 4 terpene glycosides, 2 terpene lactones, 1 naphthofurans.

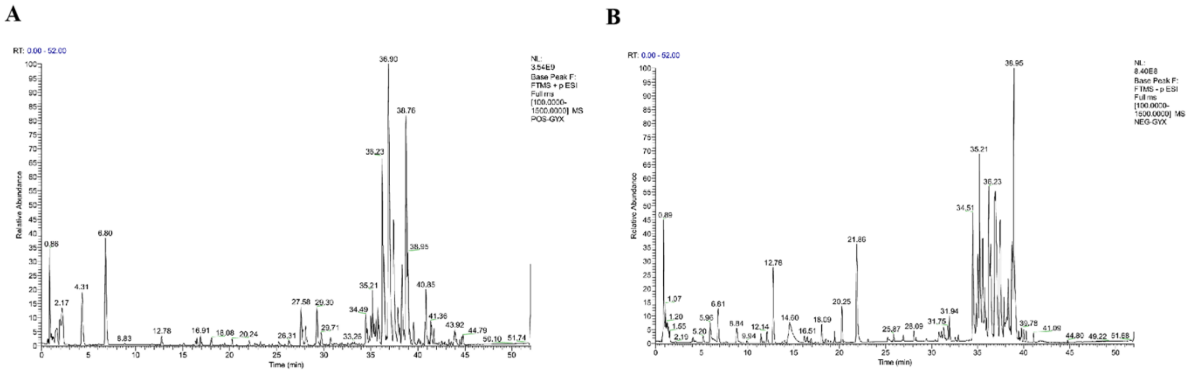

Supplementary Figure S1. The base peak ion (BPI) chromatogram of LGZG-containing serum based on UHPLC-Q-Orbitrap HRMS system in both positive (A) and negative (B) ion modes.

Supplementary Table S1 Analysis and identification of chemical constituents in LGZG-containing serum

NO.	Retention time (min)	Ion mode	Experimental (m/z)	Theoretical (m/z)	Fragment ions（m/z）	Molecular formula	Identification
1	33.85	[M+H]+	501.3561	501.3575		C31H48O5	16alpha,25 -dihydroxyeburiconic acid
2	33.56	[M+H]+	487.3403	487.3418	487.3423, 95.0864, 59.0506, 119.0859, 121.1016, 107.0861, 173.1323, 105.0707, 89.0608, 93.0707	C30H46O5	16alpha,27 -dihydroxy -dehydrotrametenoic acid
3	35.83	[M+H]+	469.3303	469.3312	-	C30H46O4	16alpha-hydroxydehydrotrametenolic acid
4	34.97	[M+H]+	547.3649	547.3629	-	C32H48O7	26-hydroxyporicoic acid dm
5	34.07	[M+H]+	529.3506	529.3524	-	C32H48O5	Poricoic acid am
6	32.47	[M+H]+	533.3494	533.3473	-	C31H46O6	Poricoic acid d
7	32.42	[M+H]+	535.3258	535.3265	-	C30H46O5	Poricoic acid g
8	15.46	[M+H]+	257.0802	257.0808	-	C15H12O4	5,7-dihydroxyflavanone
9	32.18	[M+H]+	313.1789	313.1798	-	C21H24O5	Glyasperin c

(*continue on next page*)

Supplementary Table S1 (*continued*)

NO.	Retention time (min)	Ion mode	Experimental (m/z)	Theoretical (m/z)	Fragment ions（m/z）	Molecular formula	Identification
10	19.04	[M+H]+	433.1121	433.1129	455.0945, 279.0625, 84.0819, 139.1229, 268.2013, 416.3992, 199.0213, 121.3228	C15H12O4	Isoliquiritigenin
11	34.90	[M+H]+	503.3388	503.3367	-	C30H46O3	Licoricesaponin c2 deglycosylation
12	18.64	[M+H]+	257.0801	257.0808	256.263, 257.0806, 137.0232, 147.0439, 88.0766, 57.0713, 256.1539, 102.0918	C15H12O4	Liquiritigenin
13	33.30	[M+H]+	189.1632	189.1638	84.9607, 117.0703, 91.0551, 189.1634, 131.0857, 119.086, 105.0705, 79.0556, 133.1013, 102.9707	C15H20O2	2-atractylenolide
14	27.42	[M+H]+	227.1059	227.1067	55.0193, 95.0863, 227.1066, 81.0709, 67.0555, 109.1017, 83.0865, 225.1465, 175.1483, 55.0557	C15H18O2	Atractylenolide i
15	29.14	[M-H]-	505.3545	505.3535	-	C30H48O6	16-oxoalisol a
16	35.42	[M-H]-	497.3281	497.3272	497.3313, 115.9187, 130.9419, 423.293, 100.9317	C31H46O5	29-hydroxypolyporenic acid c

(*continue on next page*)

Supplementary Table S1 (*continued*)

NO.	Retention time (min)	Ion mode	Experimental (m/z)	Theoretical (m/z)	Fragment ions（m/z）	Molecular formula	Identification
17	34.06	[M-H]-	527.3384	527.3378	467.3174, 423.3262, 527.3416, 96.9576, 78.9567, 61.9858, 59.0113, 152.9942, 386.7921, 205.676	C32H50O5	3-o-acetyl-16alpha-hydroxytrametenolic acid
18	34.67	[M-H]-	559.3650	559.3640	559.3669, 379.1818, 174.9539, 59.0113, 529.3519, 440.9189, 442.9862, 442.929, 384.4077, 78.9568	C32H48O5	3β-hydroxy-16α-acetoxy-lanosta-7,9(11), 24-trien-21-oicacid
19	31.31	[M-H]-	499.3439	499.3429	499.3448, 116.9263	C31H46O4	Polyporenic acid c
20	36.75	[M-H]-	469.3328	469.3323	469.3336, 425.3434	C30H46O4	18alpha-glycyrrhetinic acid
21	33.14	[M-H]-	485.3281	485.3272	485.3292	C30H46O5	18alpha-hydroxyglycyrrhetic acid
22	18.91	[M-H]-	505.1364	505.1352	-	C23H24O10	6"-o-acetylliquiritin

(*continue on next page*)

Supplementary Table S1 (*continued*)

NO.	Retention time (min)	Ion mode	Experimental (m/z)	Theoretical (m/z)	Fragment ions（m/z）	Molecular formula	Identification
23	29.24	[M-H]-	357.1326	357.1344	99.9235, 116.9263, 115.9185, 289.1448, 173.9607, 357.1333, 100.9313, 174.9911, 203.1068, 97.9291	C20H20O6	8-prenylated eriodictyol
24	27.80	[M-H]-	443.2059	443.2075	443.2079, 375.2186, 101.0218, 443.0107, 252.9893, 272.9966, 276.9903, 266.9879	C25H28O5	Euchrenone
25	25.51	[M-H]-	383.1142	383.1136		C21H20O6	Gancaonin b
26	20.05	[M-H]-	447.1304	447.1297	447.1292, 113.022, 271.0976, 85.027, 174.9539, 59.0113, 121.0277, 269.0458, 75.0063, 71.0113	C21H20O7	Gancaonin p -3'-methylether
27	31.15	[M-H]-	485.3281	485.3272	-	C30H44O4	Glabrolide
28	24.80	[M-H]-	359.1483	359.1500	-	C21H22O6	Glyasperin b
29	26.20	[M-H]-	363.0858	363.0874	-	C21H18O6	Glycyrol
30	22.86	[M-H]-	431.1353	431.1348	431.1352, 121.0268, 85.027, 152.9941, 78.9565, 59.0113, 113.0221, 255.1036, 99.0061, 87.0061	C21H20O6	Glycyrrhisoflavanone

(*continue on next page*)

Supplementary Table S1 (*continued*)

NO.	Retention time (min)	Ion mode	Experimental (m/z)	Theoretical (m/z)	Fragment ions（m/z）	Molecular formula	Identification
31	27.35	[M-H]-	499.1622	499.1610	-	C25H26O6	Glyurallin b
32	26.44	[M-H]-	313.1452	313.1445	-	C20H20O4	Isobavachin
33	34.46	[M-H]-	455.3170	455.3167	455.3173, 152.994, 78.9565, 112.9834, 116.9262, 393.3142, 455.2173	C30H44O4	Isoglabrolide
34	11.29	[M-H]-	593.1526	593.1512	255.0669, 593.1514, 113.0221, 119.0481, 135.0068, 85.0272, 153.0179, 59.0114, 175.0234, 99.0066	C27H30O14	Isoviolanthin
35	29.44	[M-H]-	415.1557	415.1551	-	C26H28O5	Kanzonol f
36	36.68	[M-H]-	413.2316	413.2333	-	C26H32O5	Kanzonol h
37	26.48	[M-H]-	325.1086	325.1081	325.1088, 169.0854, 304.9821, 130.085, 263.1081, 248.0852, 96.9909, 147.0427	C20H16O5	Kanzonol w
38	21.62	[M-H]-	399.1091	399.1085	-	C20H18O4	Licoflavone a
39	27.16	[M-H]-	983.4520	983.4493	-	C48H72O21	Licoricesaponin a3
40	30.18	[M-H]-	1011.4851	1011.4806	-	C50H76O21	Licoricesaponin d3

(*continue on next page*)

Supplementary Table S1 (*continued*)

NO.	Retention time (min)	Ion mode	Experimental (m/z)	Theoretical (m/z)	Fragment ions（m/z）	Molecular formula	Identification
41	29.17	[M-H]-	837.3940	837.3914	-	C42H62O17	Licorice-saponin g2
42	26.89	[M-H]-	879.4052	879.4020	-	C42H62O15	Licoricesaponine c2
43	18.64	[M-H]-	255.0664	255.0663	119.0481, 255.0665, 153.0174, 135.0066, 91.0162, 192.138, 80.9627	C15H12O4	Liquiritigenin
44	12.08	[M-H]-	433.1146	433.1140	137.0223, 313.057, 113.0225, 119.0481, 85.0271, 59.0114, 175.0224, 142.7098, 83.6986, 96.6899	C21H22O9	Liquiritin
45	34.28	[M-H]-	499.3438	499.3429	499.3449	C31H48O5	Methyl 18alpha -hydroxyglycyrrhe tate
46	26.61	[M-H]-	355.1193	355.1187	355.1189, 353.2345, 177.0543, 353.0785, 316.9856, 354.0752, 115.0375, 96.9911, 81.9506, 133.0639	C20H20O6	Sigmoidin b
47	32.86	[M-H]-	237.1493	237.1496	237.1492, 102.9545, 237.0401, 116.9266, 195.1382, 176.8822, 236.911, 164.9306, 210.4057, 116.7231	C15H20O3	Atractylenolide iii

(*continue on next page*)

Supplementary Table S1 (*continued*)

NO.	Retention time (min)	Ion mode	Experimental (m/z)	Theoretical (m/z)	Fragment ions（m/z）	Molecular formula	Identification
48	24.75	[M-H]-	267.0666	267.0663	-	C16H12O4	Dalbergin
49	14.99	[M-H]-	429.0832	429.0827	-	C15H10O4	Nordalbergin
