# Supplementary material for: Ling-gui-zhu-gan promotes adipocytes browning via targeting the miR-27b/PRDM16 pathway in 3T3-L1 cells
Source: Front Pharmacol. 2024 Aug 14;15:1386794. doi: 10.3389/fphar.2024.1386794 (PMC11349548; doi:10.3389/fphar.2024.1386794)
Supplement: Supplementary file 6 [file DataSheet3.PDF]

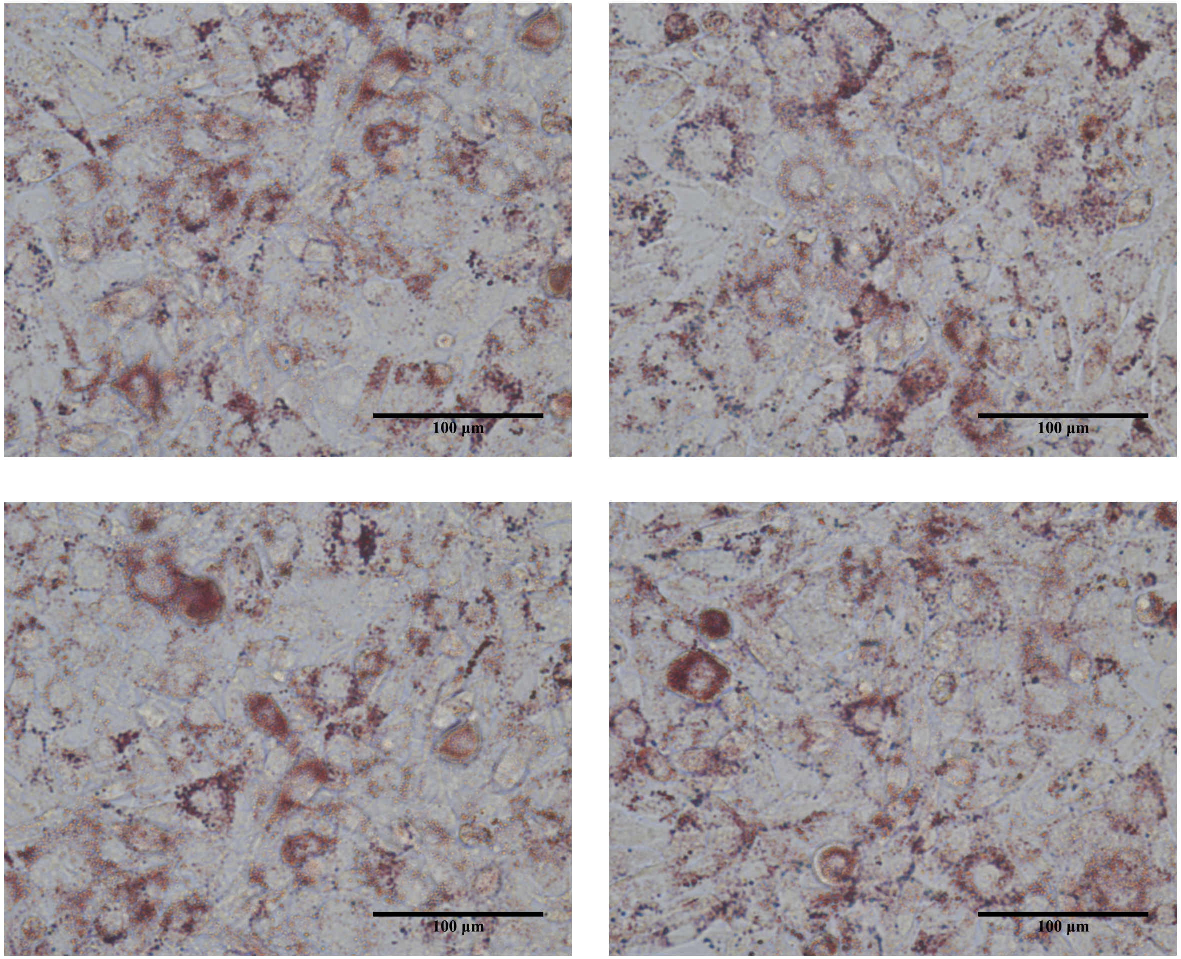

Figure 1B——no treatment (400 x)

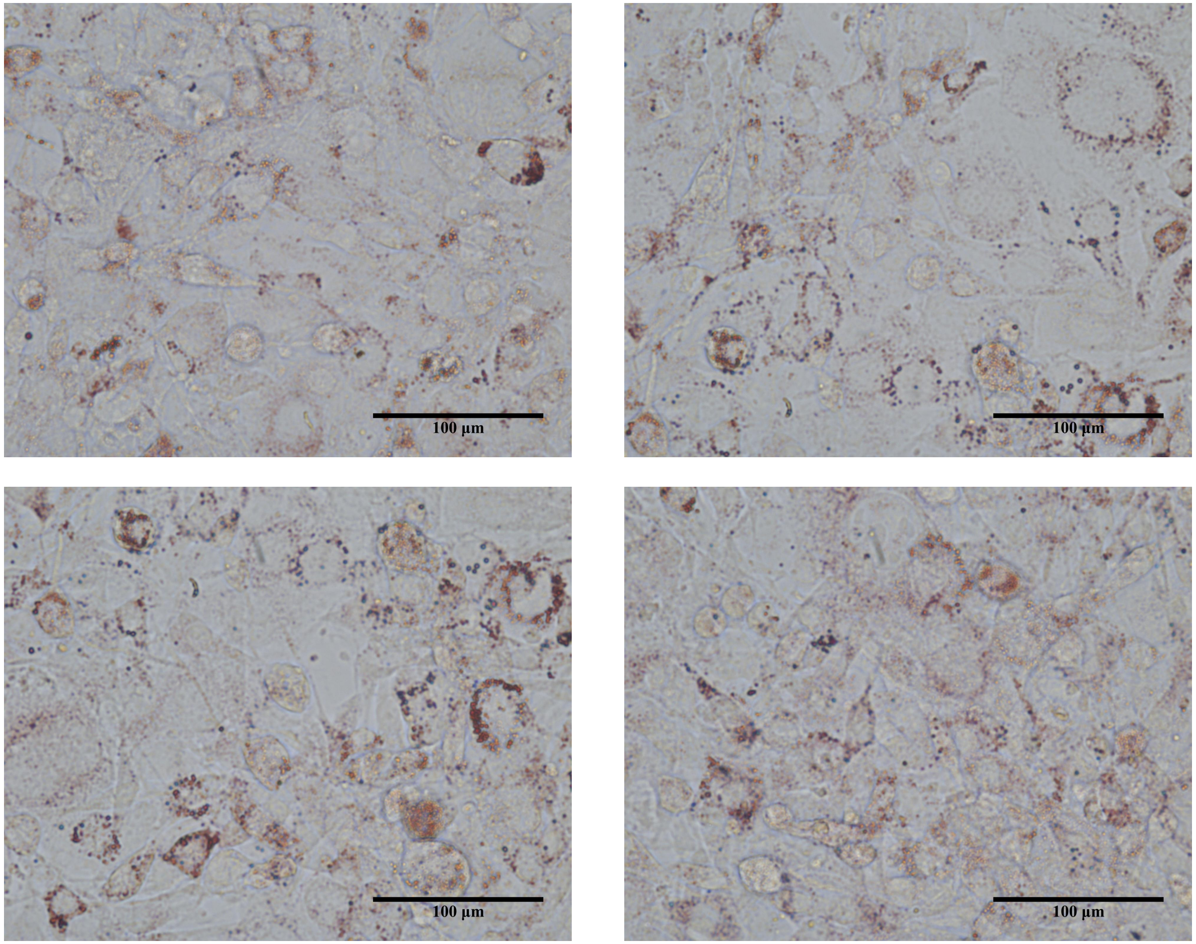

Figure 1B——metformin (400 x)

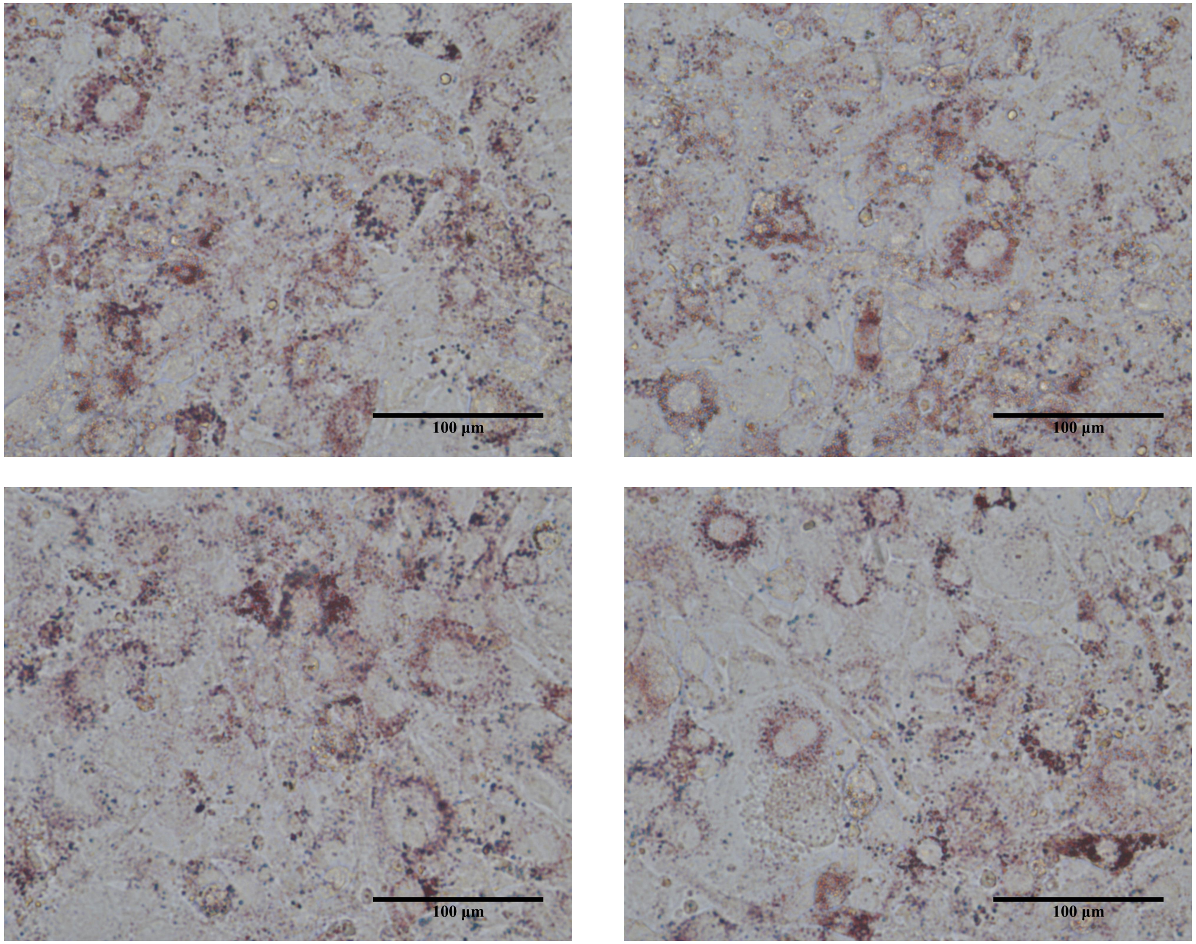

Figure 1B——1%LGZG-containing serum (400 x)

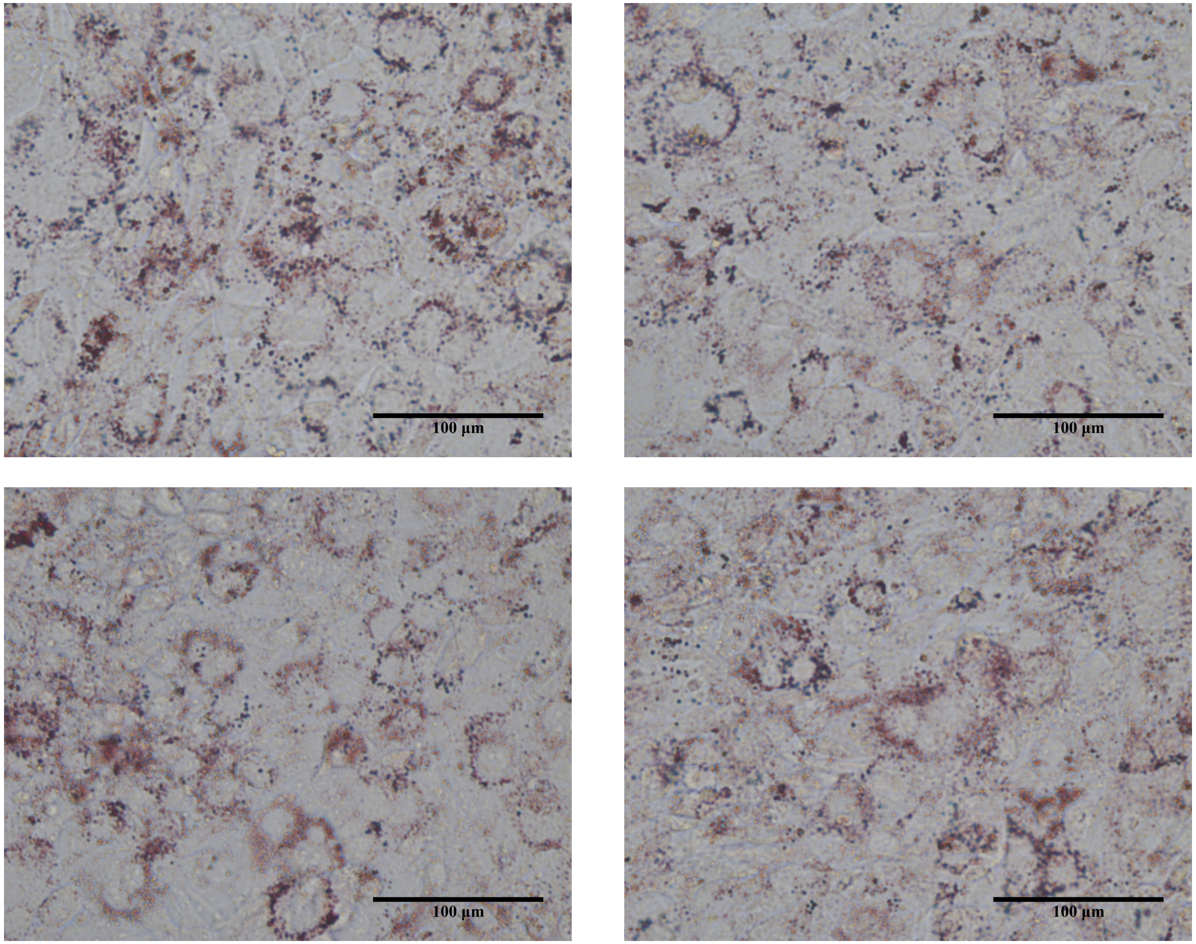

Figure 1B——5%LGZG-containing serum (400 x)

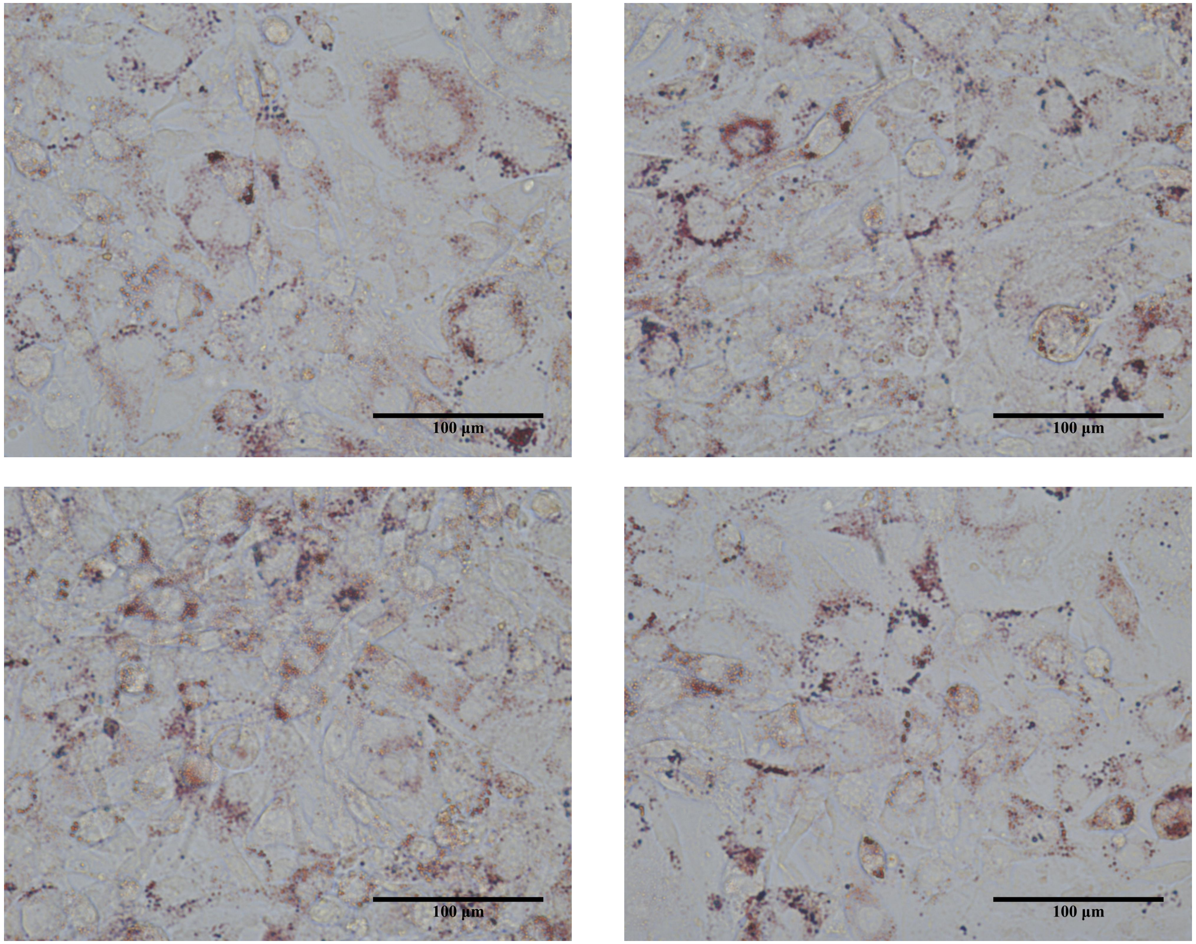

Figure 1B——10%LGZG-containing serum (400 x)
